# Translatability scoring in prospective and retrospective COVID drug development cases

**DOI:** 10.1007/s00228-023-03517-0

**Published:** 2023-06-06

**Authors:** Alexandra Wendler, Martin Wehling

**Affiliations:** Clinical Pharmacology, Faculty of Medicine, Ruprecht-Karls-University of Heidelberg, 68167 Mannheim, Germany

**Keywords:** Drug development, Translatability scoring, Drugs to treat COVID-19, Vaccines

## Abstract

**Background:**

The ongoing pandemic of severe acute respiratory syndrome coronavirus 2 has led to an enormous surge of clinical research. So far, the speed and success rate of related drug development projects, especially of vaccines, is unprecedented. For the first time, this situation allowed for the opportunistic evaluation of a translatability score, originally proposed in 2009, in a prospective manner.

**Methods:**

Several vaccines and treatments under development in clinical phase III trials were selected for translational scoring with the translatability score. Six prospective and six retrospective case studies were performed. The scores had to be determined for a fictive date before any results of the phase III trial were reported in any media. Spearman correlation analysis and a Kruskal Wallis test were performed for statistical evaluation.

**Results:**

A significant correlation between the translatability scores and the clinical outcomes in translation was found, as judged on the basis of positive/intermediate/negative endpoint studies or market approval. The Spearman correlation analysis of all cases (*r* = 0.91, *p* < 0.001), the prospective cases alone (*r* = 0.93, *p* = 0.008), and the retrospective cases alone (*r* = 0.93, *p* = 0.008) showed a strong correlation between the score and outcome; *R*^2^ demonstrated a score-derived determination of outcomes by 86%.

**Conclusions:**

The score detects strengths and weaknesses of a given project, resulting in the opportunity of selective amelioration of a project, as well as prospective portfolio risk balancing. Its substantial predictive value that has been demonstrated here for the first time could be of particular interest for biomedical industry (pharmaceutical and device manufacturers), funding agencies, venture capitalists, and researchers in the area. Future evaluations will have to address the generalizability of results obtained in an exceptional pandemic situation, and the potential adaptations of weighing factors/items to particular therapeutic areas.

**Supplementary Information:**

The online version contains supplementary material available at 10.1007/s00228-023-03517-0.

## Background

Translational science supports the successful transition of preclinical and clinical data into development and approval of new drugs. In 2009, a translatability score was proposed [1] and successfully tested later in several retrospective case studies [2]. The coronavirus disease 2019 (COVID-19) pandemic provided the opportunity to apply this score in a prospective way. Therefore, we performed an analysis of six prospective COVID-19 case studies; six retrospective cases were added to the existing ones to validate the translatability score under prospective and retrospective conditions.

The proposed score assesses the availability, quality, and predictive value of in vitro and in vivo results, clinical data, biomarkers, and personalized medicine aspects. The items are rated by a number between 0 and 5 and multiplied by a factor which reflects its translational weight. The weight factors are generally highest for items on human data, as clinical data are more indicative of successful drug development than in vitro or in in vivo animal data. Biomarkers play an important role in the score, and a separate score for the predictive value of biomarkers [3] is enclosed. This biomarker score reflects animal and human data, their proximity to the disease, accessibility, and test validity parameters such as sensitivity and specificity [3]. Recently, the translatability score was modified and applied to Alzheimer’s disease projects by others [4].

The severe acute respiratory syndrome coronavirus 2 (SARS-CoV-2) is a novel single-stranded RNA beta-coronavirus. It affects mainly the respiratory system with most severe cases presenting an acute respiratory distress syndrome [5]. The occurrence of a cytokine storm is a severe complication, which is associated with a higher risk of death, though most of the infections are mild and sometimes asymptomatic.

The urgent need of drugs to treat or vaccines to prevent this disease led to a huge number of drug/vaccine development projects; in several cases, already approved or developed drugs were tested for the use against COVID-19. This repurposing of approved drugs has the advantage that it is much faster than the development of new drugs. Knowledge on Middle East Respiratory Syndrome Coronavirus (MERS) and severe acute respiratory syndrome coronavirus (SARS CoV) was also an important factor, leading to the unprecedented speed in development of vaccines and drugs to treat COVID-19.

Nevertheless, many failures and withdrawals of approved drugs due to lack of efficacy in later phase III trials were inevitable, for example, that of hydroxychloroquine.

## Methods

Several vaccines and treatments under development in clinical phase III trials were selected. For the retrospective studies, the date of the first publication of the phase III results was defined as date of data lock. The scores had to be determined for a fictive date before any results of the phase III trial were reported in any media. For the prospective cases, the data lock was verified by colleagues who were not involved in the project, with a time stamp in the electronic mail system as potential proof of fixation of the score prior to publication of phase III study results.

Published data were retrieved by entering the name of the drug into PubMed, Web of Science, clinicaltrials.gov, medrxiv.org, biorxiv.org, and Google.

The translatability score was determined by applying the published score [1] in its original version for tocilizumab and dapagliflozin. The score was already adapted for antivirals before [6] and used for remdesivir, molnupiravir, fluvoxamine, camostat mesylate, and hydroxychloroquine. The original score was adapted for vaccines as described below.

If no data were available for a specific item of the score, no value was given. 0 was used as stopper, which renders the project untranslatable (for example, increased mortality in the treatment group). Furthermore, we defined some rules for the acknowledgment of missing data, or data which correlate with the topic but were obtained for a different but related disease. We reduced the value for a particular item by one point if no data were available for SARS-CoV-2 but for other corona viruses. If only data for unrelated viruses were available, we reduced the value by 2 or 3 points, depending on the degree of relationship.

We pre-defined three categories of outcomes, namely success, intermediate, and failed: A reduction of mortality or severe disease in phase III trials by ≥ 33% was defined as success. If the results showed beneficial effects but did not meet the 33% cut-off, an intermediate translational success was defined. If the study failed to show any benefit, the translational success was defined as failed. Approval was no prerequisite for success as, e.g., in vaccine studies, the approval criteria was a suppression of infection by 50%, regardless of mortality effects. Some approvals were rejected later and some positive results were not translated into approval due to other causes (for example, emerging variants of the SARS-CoV-2, or non-translational issues such as market competition).

For the prospective cases, we analysed the translational process of camostat mesylate, fluvoxamine, molnupiravir, and vaccines of Medicago, Curevac, and Sanofi Pasteur.

For the retrospective cases, we analysed remdesivir, chloroquine, tocilizumab, dapagliflozin, and vaccines of Biontech/Pfizer and Astra Zeneca.

### Individualization of the translatability score for vaccine development

The score had to be adapted to features of vaccine development as some do not match well with those in the cardiovascular field, for which the score was originally developed. The adaptations were made on the basis of recent literature.

For example, cultures of human bronchial epithelial cells are valuable tools for SARS-CoV-2 infection studies [7]. Further organoids and organ-on-chip can be used to assess infection with pathogens [8]. According to this valuable tools for the development of vaccines, the weight for in vitro data was increased. Additionally, potential candidates for vaccines can be tested in animal models quite reliably [9], so that the weight for animal models was also increased. To our knowledge, human genetics is not yet considered in the process of vaccine development; consequently, its weight was reduced to 1%. Model compounds and clinical trials are important for vaccine development as in the original score and have high weights as in the original score. The same is true for biomarkers and clinical endpoints; thus, their weights remain unchanged. In terms of personalized medicine aspects, the emerging variants of SARS-CoV-2 have been considered for the item “disease subclassification,” and the weight increased to 5 as these variants of concern also reflect a threat for the success of vaccination [10]. Some but not all vaccines can be adapted quickly to these variants. mRNA vaccines, for example, can be adapted quite fast, while vaccines using attenuated virus require longer development times [11].

For pharmacogenetics, the weight has been reduced to 1% as for vaccines a “one fits all vaccines strategy” is commonly used, especially in the ongoing SARS-CoV-2 pandemic, where a high vaccination rate was needed as fast as possible.

### Statistical analysis

Kruskal Wallis test with all case studies together was performed using DATAtab [12]. Spearman correlation analysis was performed for the prospective cases, for the retrospective cases, and for all cases together using DATAtab [12].

## Results

Table 1 shows the association between the score and the clinical outcome according to the criteria mentioned in the “Methods” section. For detailed scoring results of the different vaccines, see supplemental Table 1; for the drugs treating COVID-19, see supplemental Table 2. Supplemental Tables 3 and 4 present the scores for the respective leading biomarkers. In some cases, we defined more than one biomarker as leading biomarker.

**Table 1:** Association of score and translational outcome

**Compound (date of datalock)**	Translatability score*	**Clinical outcome** (success: reduction of mortality or severe disease in phase III trials by > 33%, intermediate: results showed beneficial effects but did not meet the 33% cut-off, failed: study failed to show any benefit)
**Prospective cases:**		
CVnCoV (Curevac) (28.4.2021)	4.40 Fair to good chance of success	Success: Phase III showed efficacy, (100% protection against mortality and severe disease) [17] but the emergence of new variants and problems not due to the translational process, resulted in the company’s decision not to apply for approval at the EMA/FDA
Covifenz (Medicago) (19.5.2021)	3.52 Moderate risk of failure/moderate chance of success	Success: Phase III successful (100% protection against mortality and severe disease) [30], approved in Canada
Vidprevtyn (Sanofi Pasteur) (13.10.2021)	4.21 Fair to good chance of success	Success: Phase III successful, (100% protection against mortality and severe disease) [37] marketing authorization application submitted
Camostat mesylate (7.5.2021)	1.98 Very high risk	Failed: Phase III failed, Camostat mesylate is not effective for treatment of patients with mild to moderate COVID-19 [56]
Molnupiravir (25.6.2021)	2.68 High risk	Intermediate: Phase III successful, and approved by the FDA (EUA), marketing authorisation application submitted to EMA In the analysis of all participants who had undergone randomization, the percentage of participants who were hospitalized or died through day 29 was lower in the molnupiravir group than in the placebo group (6.8% vs. 9.7%, reduction by 31%) [63]
Fluvoxamine (17.6.2021)	1.87 Very high risk	Failed: Phase III declared as successful, but the FDA has determined that the data are insufficient to conclude that fluvoxamine may be effective in the treatment of nonhospitalised patients with COVID-19 to prevent progression to severe disease and/or hospitalization. There were no significant differences between fluvoxamine and placebo for viral clearance at day 7 and hospitalisations due to COVID, all-cause hospitalisations, time to hospitalisation, number of days in hospital, mortality, time to death, number of days on mechanical ventilation, time to recovery or the PROMIS Global Physical or Mental Scale [79]. The FDA rejected the application for an emergency use authorization
**Retrospective cases:**		
Comirnaty (Biontech/Pfizer) (8.11.2020)	4.09 Fair to good chance of success	Success: Phase III successful, 100% protection against mortality and severe disease [82], approved by FDA and EMA
Vaxzveria (Astra Zeneca) (21.3.2021)	4.11 Fair to good chance of success	Success: Phase III successful [86], 100% protection against mortality and severe disease, approved by FDA and EMA
Remdesivir (28.4.2020)	2.50 High risk	Intermediate: Several phase III trials showed different results: remdesivir was superior to placebo in shortening the time to recovery in adults who were hospitalized with Covid-19 and had evidence of lower respiratory tract infection [90] 3-day course of remdesivir had an acceptable safety profile and resulted in an 87% lower risk of hospitalization or death than placebo [93] Remdesivir has no significant effect on patients with COVID-19 who are already being ventilated. Among other hospitalised patients, it has a small effect against death or progression to ventilation (or both) [143] No clinical benefit was observed from the use of remdesivir in patients who were admitted to hospital for COVID-19, were symptomatic for more than 7 days, and required oxygen support [144] approved by FDA and EMA
Hydroxychloroquine (04.5.2020)	0 (without stopper 2.15) Highest possible risk due to stopper	Failed: Phase III failed, use of chloroquine/hydroxychloroquine added to standard treatment resulted in a significant worsening of clinical status, an increased risk of renal dysfunction and an increased need for invasive mechanical ventilation [145] Hydroxychloroquine improved neither the clinical status at day 15 nor SARS-CoV-2 clearance in respiratory tract specimens [146] EUA was withdrawn by the FDA
Tocilizumab (28.7.2020)	3.61 Moderate risk of failure/moderate chance of success	Intermediate: Several Phase III trials showed different results: use of tocilizumab did not result in significantly better clinical status or lower mortality than placebo at 28 days [147], tocilizumab reduced the likelihood of progression to the composite outcome of mechanical ventilation or death, but it did not improve survival [147] Administration of IL-6 antagonists, compared with usual care or placebo, was associated with lower 28-day all-cause mortality [148] Tocilizumab may reduce the need for mechanical and noninvasive ventilation or death by day 14 but not mortality by day 28 [149] In hospitalized COVID-19 patients with hypoxia and systemic inflammation, tocilizumab improved survival and other clinical outcomes [150] In critically ill patients with COVID-19 receiving organ support in ICUs, treatment with the interleukin-6 receptor antagonists tocilizumab and sarilumab improved outcomes, including survival [151] Tocilizumab was not effective for preventing intubation or death in moderately ill hospitalized patients with COVID-19 [152] No benefit on disease progression was observed compared with standard care [153] approved by FDA and EMA
Dapagliflozin (11.4.2021)	2.06 Very high risk	Failed: Phase III failed: In patients with cardiometabolic risk factors who were hospitalised with COVID-19, treatment with dapagliflozin did not result in a statistically significant risk reduction in organ dysfunction or death, or improvement in clinical recovery [154].

*Definitions: 0–1 highest possible risk, 0 = stopper (development should be suspended); 1–2 very high risk; 2–3 high risk; 3–4 moderate risk of failure/moderate chance of success; 4–5 fair to good chance of success

The following chapters highlight the essentials of the 12 case studies (6 prospective, 6 retrospective ones).

### Prospective cases

#### CVnCoV (Curevac)

CVnCoV is an mRNA vaccine against COVID-19. The mRNA technology had already been developed for cancer vaccination and its adaptation for vaccines against COVID benefits from these data. Two vaccines, Spikevax (Moderna) and Comirnaty (Biontech/Pfizer), are based on the same principle and have already been approved. Furthermore, CV7202, a mRNA vaccine against rabies, is currently in clinical phase I and promising results have been published [13]. For cancer, data from the first clinical trials are encouraging and warrant further exploration of mRNA as a neo-antigen vaccine platform [14, 15]. The promising results for in vitro and in vivo data, the existence of successful model compounds, and the high score for the leading biomarker (virus neutralizing titre) (supplemental table 3), which is a well-established biomarker for the development of vaccines [16], lead to a high overall score of 4.4 (Table 1; Supplemental Table 1). Despite 100% protection against hospitalization and death shown in a phase III study [17], Curevac did not apply for market authorization of CVnCoV. The results of the phase III trial showed an overall efficacy of only 48.2% to prevent symptomatic COVID-19 infection [18]. This may be due to several factors. The emerging new variants of the virus (e.g., B05) reduced the efficacy. In the case of Comirnaty and Spikevax, emerging variants (e.g., B05) also caused a lower efficacy in the prevention of SARS-CoV-2 infection [19], but the vaccines still protected against severe disease and death. Furthermore, no cooperation partner was included early on, which may have resulted in a delayed start of phase III.

Despite this, we defined the outcome as success as the vaccine provided 100% protection against hospitalization and death.

#### Covifenz^®^ (Medicago)

Covifenz^®^ is a vaccine produced in tobacco plants; it is based on the spike protein of SARS-CoV-2 expressed as virus-like particles (VLPs). At the date of datalock, no in vitro data have been published for this compound, but there are in vitro and in vivo data available for a VLP vaccine against influenza [20–26]. As this virus has a low degree of relationship towards corona viruses, we graded the score results for in vitro and in vivo data down to 2, as described in “Methods.”

The VLP technology is already used for other vaccine projects, but no vaccine has been produced in plants: Gardasil against HPV is a VLP vaccine; also, Mosquirix against malaria and a vaccine against HBV (Sci-B-Vac) exist (reviewed in [27]). Elelyso, a drug for Gaucher´s disease, is produced in carrots and approved by the FDA [28]. In veterinary medicine, a vaccine against Newcastle Disease in poultry is approved and also produced in plants. Additionally, Medicago has shown in two phase III trials, that a VLP vaccine against influenza produced in tobacco plants was non-inferior than an approved vaccine [29]. Therefore, the score for model compounds and clinical trials was set on 3. The values for biomarker grading were also high, as it was the case for all analysed vaccines (supplemental table 3). This vaccine may be quickly adapted to emerging variants of the virus; this was considered in “disease sub-classification and responder concentration” (supplemental Table 1).

A phase III trial showed that the vaccine protected 100% against severe disease [30]. We therefore defined the outcome as success, which fits to the results of the translatability scoring (3.52). Covifenz^®^ was approved by Health Canada on 24th February 2022.

#### Vidprevtyn^®^ (Sanofi Pasteur)

Vidprevtyn^®^, a vaccine produced by Sanofi Pasteur and GlaxoSmithKline, is an AS03-adjuvanted soluble prefusion S protein vaccine formulation. The transmembrane domain was replaced by a T4 foldon domain and this domain assists in trimerization. In vitro and in vivo data were promising. The adjuvant AS03 was already used in several other vaccines and several other adjuvanted vaccines based on soluble protein have already been approved [31–33]. Further, another adjuvanted recombinant protein vaccine against COVID-19 showed robust immunogenicity; efficacy against COVID-19 mild, moderate, and severe illness in adults, including older adults (aged 65 years or older) was positive and safety acceptable [34, 35]. It has to be noted that this vaccine is a nanoparticle vaccine, containing the full-length spike protein, while the vaccine of Sanofi consists of the soluble form, in which the transmembrane domain is replaced. These preexisting results lead to a high score for model compounds and clinical trials (supplemental Table 1).

In the initial phase I/II trial of this vaccine, the immune response was insufficient. The authors stated that the concentration of antigen was too low, due to the fact that the monoclonal antibody used in the tests also detected host protein leading to an overestimation of antigen and an underestimation of host protein [36] (NCT04537208, registration date 3 September 2020).

Therefore, a second phase II trial was performed and interim results posted in a press release on 17th March 2021: the adjuvanted recombinant vaccine candidate triggered a strong immune response amongst adults of all age groups with 95 to 100% seroconversion rates and levels of neutralizing antibodies that were comparable to those generated by natural infection (NCT04762680, registration date February 21, 2021).

The vaccine protected against severe disease and hospitalization in a phase III trial by 100% [37]. This trial was rated successful and fits with the results of our scoring (4.21).

#### Camostat mesylate

Camostat inhibits transmembrane protease serine subtype 2 **(**TMPRSS). Originally, camostat was used as treatment for pancreatitis in Japan, but the mechanism of action seems not yet to be clearly understood [38].

TMPRSS2 is a mostly membrane-bound serine-protease that promotes metastasis of prostate cancer and increases expression of some matrix metalloproteinases. Its physiological function is unknown but it has been shown to facilitate the entry of several viruses (reviewed in [39]).

Positive in vitro data of hypothetical importance for COVID-19 treatment exist for camostat [39–46]. Since there were no in vivo data for SARS-CoV-2, but other coronaviruses like SARS-CoV and MERS [47–52], we reduced the score for in vivo data by 1 (supplemental Table 2). Since SARS-CoV-2 also uses several cellular proteases as entry activators, which are in part camostat insensitive, it has been stated that inhibitor combinations against multiple protease activators might be more effective than camostat alone [45].

Despite promising in vitro and in vivo data, no positive clinical data or successful model compounds could be detected. Twenty-seven studies were listed on clinical trials.gov on 29th of March 2021. These studies test camostat as treatment against COVID-19 alone or explore the combination of camostat with other drugs like remdesivir, bicalutamide, hydroxychloroquine, or niclosamine solution. Further aspects of these studies were disease severity, prophylaxis, and effects on coagulation (for details see [53]).

The biomarker scoring resulted in an intermediate result (supplemental Table 4), as the dynamics of viral load as biomarker has some weaknesses. For example, the viral load does not necessarily correlate with onset of disease. Further, viral RNA may be detected after recovery of the patients. Data for personalized medicine were contradictive [54]. Due to these data, the score for translatability was low (1.98).

Indeed, no positive clinical phase III trial results have been reported for camostat in context with COVID-19 yet, but there are negative data [55, 56]. Meanwhile, the SARS-CoV-2 variant omicron uses an altered cell entry pathway, favouring a TMPRSS2-independent endosomal entry pathway [57], rendering camostat ineffective.

#### Molnupiravir (Lagevrio^®^) (Merck Sharp and Dohme)

Molnupiravir (EIDD-2801/MK-4482) is a nucleoside analog like remdesvir, but the two drugs differ from each other in that remdesivir acts as a chain terminator, while molnupiravir induces mutations during viral RNA replication.

Molnupiravir has been shown be active in vitro and vivo; it prevented the transmission of SARS-CoV-2 in ferrets [58] and reduced the viral load in hamster lung tissue [59]. Therefore, high values were given for starting evidence. A study showed that molnupiravir may promote mutagenesis in the host due to incorporation of the 2′-deoxyribonucleotide form of molnupiravir in DNA; thus, the value for in vitro evidence was reduced to 3 (supplemental Table 2).

Two phase II trials, in hospitalized and in non-hospitalized patients, have been performed. Interim analysis showed a benefit only in the non-hospitalized patients [60]. Therefore, the trial with hospitalized patients was discontinued and a phase III trial with non-hospitalized patients was started. Therefore, we rated clinical trials with 3. For the use of antivirals, the timing is very critical which was underlined by the fact that in hospitalized patients, molnupiravir has no beneficial effect. In our translatability score, this point is reflected by the personalized medicine aspects (supplemental Table 2).

Although both used the same biomarker, the score for the biomarker grading was higher for molnupiravir than in camostat (supplemental Table 4). This is due to the fact that in the case of molnupiravir human data, which are directly related to this project are available [61]. Furthermore, in the meantime (datalock of molnupiravir was later than for camostat), there were studies, which show a correlation of viral load and intervention. The translatability scoring result was 2.68 (Table 1; supplemental Table 2), indicating relatively high risk of failure.

Molnupiravir was first approved in the UK [62] on 4th November 2021 for the treatment of mild to moderate COVID-19 in adults with a positive SARS-CoV-2 diagnostic test and at least one risk factor for developing severe illness. Besides promising interim results of the phase III study, the final results showed a protection against severe disease and hospitalization of 30% [63]. Therefore, the phase III results did not meet our criteria for success and the outcome for molnupiravir was defined as intermediate.

#### Fluvoxamine

Fluvoxamine is indicated to treat obsessions and compulsions in patients with obsessive compulsive disorder, or depression. It selectively blocks the reuptake of serotonin at the sodium-dependent serotonin transporter of the neuronal membrane, enhancing actions of serotonin on 5HT1A receptors [64]. The putative mechanism of action of fluvoxamine in the case of a SARS-CoV-2 infection is highly speculative. Possible mechanisms discussed include platelet aggregation, depletion of serotonin content of platelets, inhibition of blood clotting [65], mast cell degranulation, decrease in histamine release from mast cells [66], decrease of mRNA levels of protease-1 in mast cells [67], reduction in cytokine storms in COVID-19 patients because of atypical response of mast cells to SARS-CoV-2 [65], lysosomotropism [68], lysosomal trafficking to escape from infected cells [69], or functional inhibition of acid sphingomyelinase [70]. Furthermore, the sigma-1 receptor was proposed to be involved in the mechanism of action of fluvoxamine [71] by protecting against the inflammatory response and lethal septic shock [72]. Another putative mechanism is the elevation of melatonin levels by fluvoxamine, resulting in a mitigation of inflammation [73–75]. Besides the unclear mechanism of action, no animal data are available for the treatment of COVID-19 with fluvoxamine. Studies with other selective serotonin reuptake inhibitors showed an association with lower risk of intubation or death of COVID-19 patients in a multicentre observational retrospective cohort study [76], but fluvoxamine was not tested in this study. A prospective cohort study showed that the treatment with fluvoxamine 50 mg twice daily resulted in a incidence of hospitalization of 0% for the fluvoxamine group compared to 12.5% in the control group (no treatment) [77]. An outpatient trial with 152 persons showed that fluvoxamine decreased clinical progression, defined as hypoxia (< 92% oxygen saturation) coupled with either shortness of breath or hospitalization, from 8% (6 of 72) with observation alone to 0% (0 of 80) with fluvoxamine at up to 300 mg daily (*p* = .009) [78]. Both studies had several weaknesses so that the clinical evidence for fluvoxamine is quite low (supplemental Table 2). As the mechanism of action of fluvoxamine is highly speculative, the biomarker development in this project remains mainly elusive (supplemental Table 4). The endpoint strategy differs in the studies and evaluates decreases in O_2_ saturation, the presence of dyspnoea and/or hospitalization, or the rate of emergency visits. As some of the studies were performed by home monitoring of O_2_ monitoring, the reliability of these studies is questionable.

A phase III clinical trial showed that treatment with fluvoxamine among high-risk patients with early diagnosed COVID-19 reduced the need for hospitalization defined as retention in a COVID-19 emergency setting or transfer to a tertiary hospital but these parameters may have been influenced by psychological drug effects. More objective parameters would have been respiratory rate or oxygen saturation. There were no significant differences between fluvoxamine and placebo for viral clearance at day 7 (*p* = 0·090) and hospitalizations due to COVID (*p* = 0·10), all-cause hospitalizations (*p* = 0·09), time to hospitalization (*p* = 0·11), number of days in hospital (*p* = 0·06), mortality (*p* = 0·24), time to death (*p* = 0·49), number of days on mechanical ventilation (*p* = 0·90), time to recovery (*p* = 0·79) or the PROMIS Global Physical (*p* = 0·55), or Mental Scale (*p* = 0·32) [79]. Therefore, we could not state that a robust benefit has been demonstrated by this study.

This was underlined by the fact that the FDA has determined the data as insufficient to conclude that fluvoxamine may be effective in the treatment of non-hospitalized patients with COVID-19 to prevent progression to severe disease and/or hospitalization. In conclusion, the FDA rejected the application for an emergency use authorization. The results of the translatability scoring of 1.87 (Table 1; supplemental Table 2) correlated with this outcome.

### Retrospective cases

#### Comirnaty^®^ (Biontech/Pfizer)

Comirnaty^®^ contains the spike protein mRNA of the original strain of SARS-CoV-2. It was the first vaccine which was approved in this context and was the first approved anti-infective vaccine in humans based on the new mRNA technology. In vitro and in vivo data showed promising results and were complemented by several clinical trials for vaccines against other infectious diseases and cancer (all reviewed in [80]). Therefore, a broad spectrum of basic and clinical research had already been done. In the translatability score, this is reflected in high values for in vitro and in vivo data, clinical trials, and model compounds (supplementary table 1). As for all vaccines, the dynamics in viral load used as biomarker yielded a high score (supplementary Table 3), as the biomarker had already been established.

The mRNA technology can be developed and modified very quickly [81], which is an important feature in pandemic situations with newly emerging variants. In contrast, vaccines using attenuated virus need a long time to be developed [11]. We honoured this aspect in the point “Disease sub-classification and responder concentration.” Meanwhile, FDA and EMA approved an adapted vaccine as several variants have emerged, which render the original vaccine less effective.

A phase III trial showed 95% protection against COVID-19 [82]. Comirnaty^®^ was first approved in the UK on 2nd December 2020, followed by approvals by FDA and EMA. The high value in the translatability score correlates well with the success of the vaccine, which was and is an important tool to fight the COVID-19 pandemic.

#### Vaxzevria^®^ (Astra Zeneca)

Vaxzevria^®^ (ChAdOx1 nCoV-19 vaccine, AZD1222) consists of the replication-deficient simian adenovirus vector ChAdOx1, containing the full-length structural surface glycoprotein (spike protein) of SARS-CoV-2, with a tissue plasminogen activator leader sequence [83]. In vitro and in vivo data were promising, and the same system was used for a vaccine against MERS-CoV, which was shown to protect non-human primates against an infection with MERS-CoV [84, 85]. Several phase 1 and 2 studies were performed successfully leading to a high score for clinical trials (supplementary Table 1). As for the mRNA-based vaccines, this vector-based vaccine can be rapidly adapted to emerging variants of the virus.

The vaccine shows 79% vaccine efficacy at preventing symptomatic COVID-19 and 100% efficacy against severe or critical disease and hospitalization and therefore meets our criteria for success [86]. It was approved by the EMA on 29th January 2021.

#### Remdesivir (Veklury^®^) (Gilead Sciences)

Remdesivir (Veklury^®^) is an inhibitor of the viral RNA-dependent RNA polymerases and was originally developed against ebola but not approved for this indication. Its active form GS-443902 (an analog of adenosine triphosphate) selectively inhibits viral RNA polymerases and has broad-spectrum activity against coronaviruses.

In vivo, remdesivir showed therapeutic efficacy in SARS-CoV-2-infected rhesus monkeys [87]. There were no phase II results published for the treatment of COVID-19 or other coronavirus diseases with remdesivir until the date of data lock (29th of April 2020). Due to the lacking clinical COVID trials, the score was set on 2 for clinical trials. Favipiravir is another nucleoside analogue, which is in clinical trials. Some of these trials showed beneficial effects of favipiravir, but all had several limitations, so that further studies are needed [88]. Ribavirin, another nucleotide analogue, was tested against SARS and MERS and SARS CoV-2, and may have potential benefits, especially in combination with other therapies, but further research is needed [89]. Without really successful model compounds available, the value has been set on 1. In conclusion, the human evidence was very low, resulting in a low score (Table 1; supplementary table 2).

Several phase III studies were published on 29th of April 2020 [90–92], leading to an Emergency Use Authorization for remdesivir for the treatment of hospitalized patients with severe COVID-19 on May 1, 2020, by the FDA. These early studies all together did not meet our 33% criteria, and outstandingly positive results seem not to be corroborated. Therefore, we defined the outcome as intermediate. Meanwhile, a study on early use of remdesivir [93] has shown that this earlier application results in an 87% lower risk of hospitalization or death though deaths were not observed in this study. The clinical judgment was performed before this late trial was published, and the result on deaths is still unclear. The translatability scoring results in a value of 2.5, which fits well with the originally proposed intermediate outcome. It remains debatable whether this late study would qualify the remdesivir case as overall success in this context, but both assessments are considered in the statistical analysis (see Statistical analysis of results below). In general, the translatability score at fictive data lock should be calculated for the emerging phase 3 trial; future attempts for responder concentration as in the second phase 3 trial for remdesivir should trigger a re-evaluation of the score in the light of data at the beginning of the new trial.

#### Hydroxychloroquine

Hydroxychloroquine, an old antimalarial drug, has been shown to be effective in vitro against SARS-CoV-2 [94–99] and in vivo against other coronaviruses [94, 100]. In the emergency use authorization issued on 28 March 2020, the FDA acknowledged that the approval was based on “limited in-vitro and anecdotal clinical data.” Clinical data were available, but the results have been inconsistent and many studies were of low quality [101–112]. A study reported increased mortality in the treatment group [113], but the paper was later retracted due to some serious concerns about the quality of the study [114]. Another study also reported increased mortality [115]. By April 24, 2020, the FDA issued a Drug Safety Communication warning about potentially fatal prolongations of the QTc interval detectable on 12-lead electrocardiograms and risks of other serious cardiac arrhythmias. The emergency use authorization was revoked on 15th June 2020.

The translatability scoring resulted in a value of 0 for clinical trials as hints for increased mortality were detected, rendering the project untranslatable (0 as a stopper). Without the stopper, a value of 2.15 was achieved (Table 1; supplemental Table 2).

#### Tocilizumab (RoActemra^®^) (Hoffmann-LaRoche)

RoActemra^®^ is a monoclonal antibody against interleukin-6 (IL-6). IL-6 is produced in response to systemic inflammation, and, thus, is important in severe COVID-19 and respiratory failure. Tocilizumab interferes with the IL-6 soluble and membrane binding site of the receptor. Findings showed that IL-6 receptor (IL-6R) variants mimicking therapeutic inhibition of IL-6R are associated with lower risk of being hospitalized for COVID-19, a phenotype that correlates with disease severity [116].

At the time of datalock (28th July 2020), several phase II trials were ongoing and some studies showed clinical benefit [117, 118]. Furthermore, smaller studies, in part retrospective, also demonstrated clinical improvements by tocilizumab [119–135], though one smaller study was negative [136].

C-reactive protein (CRP) was used as leading biomarker as this biomarker was used in nearly all studies on tocilizumab; it reflects inflammation, and was shown to be prognostically predictive in COVID-19. Furthermore, CRP levels serve as a reliable surrogate for IL-6 bioactivity [137, 138]; therefore, the biomarker score was high (supplementary table 4). As most of the studies used death as endpoint, the related score for surrogate and endpoint strategy was also high (supplementary Table 2).

The overall score of 3.61 was defined as moderate risk of failure/moderate chance of success and therefore fits with the real outcome. There were several trials showing a low effect on mortality, not meeting our 33% criteria, but there were also beneficial effects in relation to the need of mechanical ventilation. Therefore, we defined the outcome as intermediate.

#### Dapagliflozin

Dapagliflozin is a sodium-glucose co-transporter-2 inhibitor (SGLT2-I) reducing the renal reabsorption of glucose thereby decreasing plasma glucose levels. Potential anti-inflammatory effects have been reported for SGLT2-I [139]. It was hypothesized that dapagliflozin may prevent severe COVID-19 infections by antagonizing the lowering of cytosolic pH and reducing the viral load [140]. In type II diabetes mellitus, dapagliflozin lowered CRP, IL-6 levels, and ferritin, though for CRP contradictory results exist [139].

No in vitro, in vivo or clinical data were published until the date of datalock (12th April 2021), and no model compounds were available. Therefore, no value was given for these points of the score. The disease subclassification was well defined as patients in the trial must present with one of the following risk factors for severe disease: hypertension, type 2 diabetes, atherosclerotic cardiovascular disease, heart failure (with either reduced or preserved left ventricular ejection fraction), chronic kidney disease stages 3 to 4 (estimated glomerular filtration rate between 25 to 60 mL/min/1.73 m^2^). Due to this fact, the score was medium for this point (supplementary table 2). The highest value was reached for the biomarker scoring, because CRP is an established biomarker (supplementary Table 4). Nevertheless, the lack of many important data for dapagliflozin resulted in a low score indicating a high risk for failure (1.95). Indeed, the phase III study of dapagliflozin for COVID-19 failed (NCT04350593, registration date 17 April 2020).

### Statistical analysis of results

**Fig. 1:**
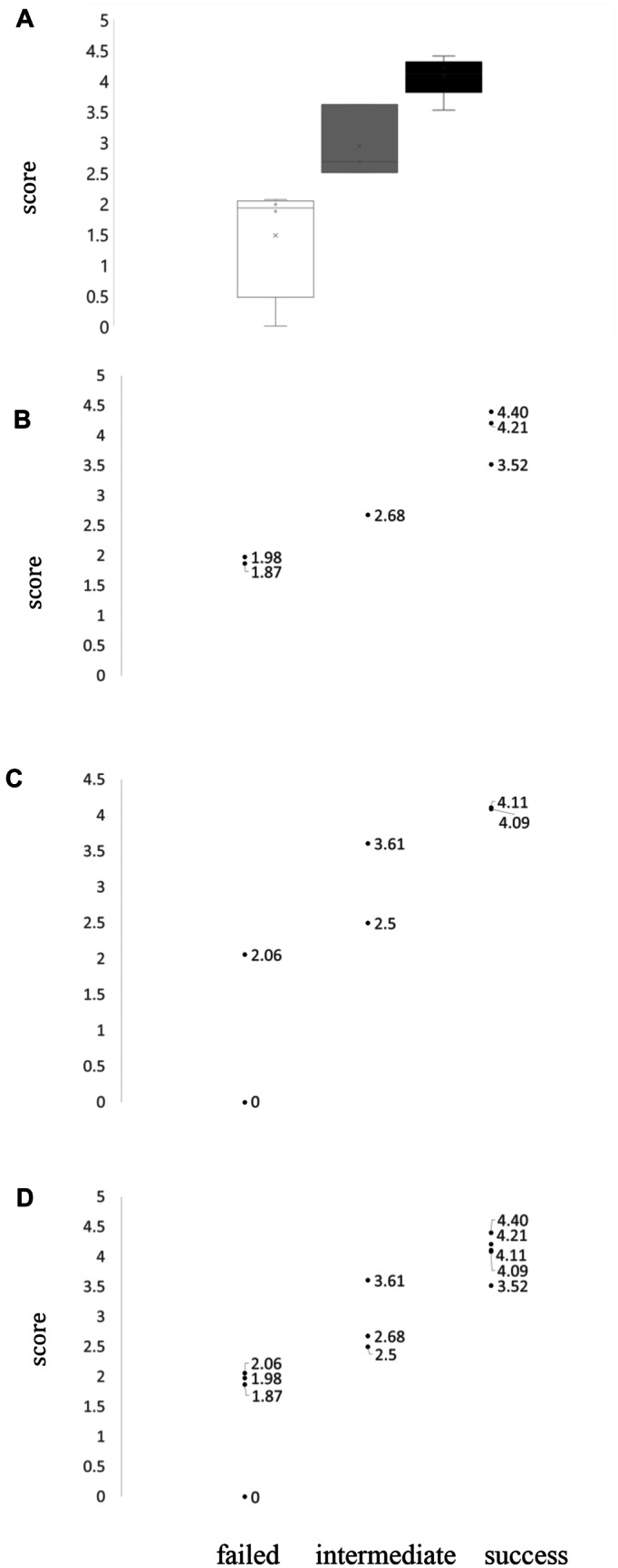
Correlation of results of the translatability scoring and clinical outcome (success: reduction of mortality or severe disease in phase III trials by > 33%; intermediate: results showed beneficial effects but did not meet the 33% cut-off; failed: study failed to show any benefit). **A** Box plot of all cases showing the distribution of the score results in the different categories. White: failed outcome, grey: intermediate outcome, black: successful outcome. **B** Scatterplot of the translatability score of prospective cases sorted by outcome (failed, intermediate, success). **C** Scatterplot of the translatability scores of retrospective cases sorted by outcome (failed, intermediate, success). **D** Scatterplot of the translatability scores of all cases sorted by outcome (failed, intermediate, success)

The Kruskal-Wallis test showed a significant difference between the three categories and the scores (two-tailed, *p* = 0.01). The subsequent Dunn-Bonferroni test demonstrated a significant difference between the group of failed and successful cases (*p* = 0.008) (Fig. 1A).

The Spearman correlation analysis (Fig. 1B–D) of all cases (*r* = 0.91, *p* < 0.001), the prospective cases alone (*r* = 0.93, *p* = 0.008), and the retrospective cases alone (*r* = 0.93, *p* = 0.008) showed strong correlations between score and outcome. *R*^2^ demonstrated a score-derived determination of outcomes by 86% for the prospective cases, the same for retrospective and 83% for all cases. ROC analysis and sensitivity/specificity analysis were not possible, due to the paucity of data and the presence of three categories.

As discussed in the case study for remdesivir, the inclusion of a secondary phase 3 study in the results could improve its clinical outcome from intermediate to success. For this outcome of remdesivir, the Spearman correlation analysis would result in the following statistical parameters: *r* = 0.82, *p* = 0.001, *R*^2^ = 0.6724 for all cases, *r* = 0.8, *p* = 0.055, *R*^2^ = 0.64 for the retrospective cases alone. The Kruskal Wallis test still showed a significant difference between the categories and the scores (*p* = 0.02). The subsequent Dunn-Bonferroni test detected a significant difference between the group of failed and successful cases (*p* = 0.016).

## Discussion

We applied the translatability score proposed by the senior author in 2009 to newly developed or repurposed drugs against COVID-19, or to newly developed COVID-19 vaccines, with 6 of the 12 case studies being prospective for the first time.

A very strong correlation of the score and the clinical success was found, meaning that the clinical outcome of the development was precisely predicted in nearly all cases. These strong correlations do not only indicate robust translatability prediction but also validate those author-based assumptions in the original proposal of the translatability score to some extent [1]. This validation of the score includes those assumptions set up for antivirals and anti-COVID drugs; it is restricted to those areas, though the former retrospective case studies [2] validated the score in other disease areas as well. However, the good correlations found in this work are not powered to optimize the individual weighing factors or scoring values. This would require many more case studies not being available at this time.

All compounds labelled by a high translatability score have been successful in phase III and except CVnCoV; all those drugs have been approved for human treatment. The failure of CVnCoV is in part due to the emergence of new variants of the virus. Even the vaccines from Biontech/Pfizer and Astra Zeneca showed significantly lower efficacy for the variants [19]. Therefore, the failure of CVnCOV seems to dependent rather on organizational than on translational issues, resulting in a delay of the process, which is problematic as new variants arrive.

All cases in which the score indicated a very high risk (1–2) have failed in phase III. For dapagliflozin, a huge gap of data existed when the phase III was started without in vitro/in vivo evidence that the compound may be useful in the case of COVID-19; thus, the start of the phase III seemed to be exclusively hypothesis-driven [140].

In the remdesivir case, the translatability scoring was performed at a fictive data lock prior to the first phase 3 trial which showed intermediate clinical outcomes according to our criteria. A later, secondary phase 3 trial representing a responder concentration attempt (earlier drug application) was successful. The scoring does not always cover such secondary modifications and should be refined before each pivotal trial, but as the development pace in COVID is too rapid to clearly separate those approaches, we recalculated the correlations with remdesivir rated as success. The Kruskal-Wallis test and the Spearman correlation remained significant for all cases. The correlation for retrospective cases was no longer significant at a borderline *p* = 0.055.

Translatability scoring may be relevant to leaders, funders, or groups involved in biomedical development projects, but the diversity of projects and therapeutic areas requires customized approaches. The translatability score used here was modified by Cummings (2020), who developed a translational scoring system that was specific to both the Alzheimer’s disease area and the translational stage: translational scoring 1 was for compounds being considered for Phase 1, translational scoring 2 for candidate drugs being considered for Phase 2, translational scoring 3 for candidate drugs being considered for Phase 3, and translational scoring 4 for candidate drugs being submitted for regulatory review and approval [4, 141].

This example shows that depending on the medical area, the score may need adaptation in terms of both items and weighing factors as already proposed for the score used here and 5 therapeutic areas [6].

Our current experience indicates that the thorough analysis of a case is laborious and — estimated from this study — will require about 30–40 working hours of one skilled person. This, however, is a small investment given the huge clinical and monetary potential of strengthening weaker projects or redirecting funds into better developed ones. Ideally, the translatability assessment should be performed by researchers skilled in both preclinical and clinical capabilities, in particular in clinical trials. Regarding the professional background, clinical pharmacologists (for drug projects) with experiences in pharmaceutical industry appear to be most suitable. Most drug companies have addressed the challenges of translation by establishing specialized departments, and access to professional specialization in and teaching of translational science in medicine has become easier in recent years [142]. However, as some of the COVID-projects or other recently failed projects show, the translational crisis is not yet over and structured prospective scoring may help to further improve the outcomes.

### Limitations

Future evaluations have to address the issue of generalizability of these results obtained in an exceptional pandemic situation, and the potential adaptations of weighing factors/items to particular therapeutic areas. In order to start validation of the score in other disease areas, further prospective case studies are needed.

We are aware of the fact that the number of prospective case studies is low, though the retrospective ones were not essentially different in their predictive precision. Due to a high work load for every single case and the limitation of COVID-projects, more cases could not be studied at present.

It has to be acknowledged that a publication bias may exist, as not all data were published at the day of data lock and negative date are often published later or may not be published at all.

Public sources of information were carefully and frequently screened, but some information may have still been missed, and private, unpublished information was not requested from potential authors/owners.

As the score is not meant as a fixed system, adaptations may be performed by everyone who uses the score before starting the evaluation of projects. In order to keep the risk of bias as small as possible, it is important that the adaptations are made before the evaluation of projects. They should not be adapted during the process of evaluation as this may introduce inappropriate bias. Yet, the adaptation carries a risk of bias, and depends on the expertise of the persons, who undertakes the adaptations.

We are further aware that the involvement of two or more raters for every case would have been more accurate, but due to limited resources and the considerable work load for each case, this was not possible.

The scoring system has some weaknesses that may require future adjustments: the application of the score showed for some projects that the weight of biomarkers might be too high as highly predictive biomarkers strongly contribute to the translatability score though the project may not have any translational foundation; a well-developed biomarker may, thus, overrun deficiencies in other areas. Yet, the development and use of biomarkers is highly important in the translational process, but cases may exist in which the weighing of biomarkers should be downgraded.

## Conclusions

The translatability score detects strengths and weaknesses of a given project, resulting in the opportunity of selective amelioration of a project, as well as prospective portfolio risk balancing. Its substantial predictive value that has been demonstrated here for the first time could be of particular interest for biomedical industry (pharmaceutical and device manufacturers), funding agencies, venture capitalists, and researchers in the area. Future evaluations will have to address the generalizability of results obtained in an exceptional pandemic situation, and the potential adaptations of weighing factors/items to particular therapeutic areas.

## Supplementary Information

Below is the link to the electronic supplementary material.

Supplementary file1 (DOCX 272 kb)

Supplementary file2 (DOCX 338 kb)

Supplementary file3 (DOCX 152 kb)

Supplementary file4 (DOCX 126 kb)

## Data Availability

All data generated or analysed during this study are included in this published article and its supplementary information files.

## Abbreviations

COVID-19: Coronavirus disease 2019
SARS-CoV-2: Severe acute respiratory syndrome coronavirus 2
MERS: Middle East Respiratory Syndrome Coronavirus
SARS CoV: Severe acute respiratory syndrome coronavirus
VLPs: Virus-like particles
TMPRSS: Transmembrane protease serine subtype 2
FDA: US Food and drug administration
EMA: European medicine agency
CRP: C-reactive protein
IL-6: Interleukin-6
IL-6R: Interleukin-6 receptor
SGLT2-I: Sodium-glucose co-transporter-2 inhibitor
