# Supplementary material for: Translatability scoring in prospective and retrospective COVID drug development cases
Source: Eur J Clin Pharmacol. 2023 Jun 6;79(8):1051–71. doi: 10.1007/s00228-023-03517-0 (PMC10243273; doi:10.1007/s00228-023-03517-0)
Supplement: Supplementary file 1 — Supplementary file1 (DOCX 272 kb) [file 228_2023_3517_MOESM1_ESM.docx]

**Supplementary table 1: translatability scores for vaccines**

|  |  | **prospective** | | | **retrospective** | |
| --- | --- | --- | --- | --- | --- | --- |
| **Data Lock** |  | **28.4.2021** | **19.5.2021** | **13.10.2021** | **21.3.2021** | **8.11.2020** |
| **Compound** |  | **CVnCoV**  **(Curevac)** | **Covifenz**  **(Medicago)** | **Vidprevtyn**  **(Sanofi Pasteur)** | **Vaxzevria**  **(Astra Zeneca)** | **Comirnaty**  **(Biontech)** |
|  | Weight (%) |  |  |  | Score x weight/100 |  |
| ***Aspect*** |  |  |  |  |  |  |
| **Starting evidence** |  |  |  |  |  |  |
| *In vitro* data including animal/viral genetics | 4 | 0.20 [1, 2] | 0.08 [3-9] | 0.20 [10] | 0.20 [11, 12] | 0.20 [13] |
| *In vivo* data including animal genetics | 5 | 0.25 [14-17] | 0.10 [9, 18-20] | 0.25 [10] | 0.25 [11, 12] | 0.25 [21] |
| Animal disease models | 5 | 0.15 [22, 23] | 0.10 [22, 23] | 0.15 [22, 23] | 0.15 [22] | 0.15 [22] |
| Data from multiple species | 3 | 0.15 [14-16] | 0.03 [18-20, 24] | 0.09 [10] | 0.09 [11, 12] | 0.15 [21] |
| **Human evidence** |  |  |  |  |  |  |
| Genetics | 1 | 0.01 [25] | 0.01 [25] | 0.01 [25] | 0.01 [25] | 0.01 [25] |
| Model compounds | 13 | 0.65 [26-32] | 0.39 [33-40] | 0.52 [39-46] | 0.52 [47] | 0.52 [48-52] |
| Clinical trials | 13 | 0.65 [29] [26, 28, 30-32] | 0.39 [53, 54] | 0.52 [41-43, 55, 56] | 0.65 [57-60] | 0.52 [26, 48, 50-52, 61-63] |
| **Biomarkers for efficacy and safety prediction** |  |  |  |  |  |  |
| Biomarker grading | 24 | 1.2 | 1.2 | 1.2 | 1.2 | 1.2 |
| Biomarker development | 13 | 0.39 [64] [16] | 0.52 [65, 66] | 0.52 [10, 55, 67, 68] | 0.39 [57-60] | 0.39 [13, 26, 61, 62] |
| **Proof-of-mechanism, proof-of-principle**  **and proof of concept testing** |  |  |  |  |  |  |
| Biomarker strategy | 5 | 0.25 [14, 15] [16] | 0.20 [53] | 0.25 [10, 55] | 0.20 [57-60] | 0.20 [21, 26, 61] |
| Surrogate or endpoint strategy | 8 | 0.24 NCT04652102 and NCT04674189) [69-71] | 0.24 NCT04450004, NCT04636697[72, 73] | 0.24 NCT04904549, NCT04762680  [56] | 0.24 [57-60] NCT04516746 NCT04973449 | 0.24 NCT04380701 NCT04368728  [61] |
| **Personalized medicine aspects** |  |  |  |  |  |  |
| Disease sub-classification and responder concentration | 5 | 0.25 [74, 75] | 0.25 [65, 66, 74, 75] | 0.25 [74, 75] | 0.20 [76] | 0.25 [76] |
| Pharmacogenetics | 1 | 0.01 [77] | 0.01 [77] | 0.01 [77] | 0.01 [77] | 0.01 [77] |
| **Sum** | **100** | **4.40** | **3.52** | **4.21** | **4.11** | **4.09** |

**References:**

1 Kumar S, Sarma P, Kaur H, Prajapat M, Bhattacharyya A, Avti P, Sehkhar N, Kaur H, Bansal S, Mahendiratta S, Mahalmani VM, Singh H, Prakash A, Kuhad A, Medhi B (2021) Clinically relevant cell culture models and their significance in isolation, pathogenesis, vaccine development, repurposing and screening of new drugs for SARS-CoV-2: a systematic review. Tissue Cell 70: 101497 DOI 10.1016/j.tice.2021.101497

2 Rahimi A, Mirzazadeh A, Tavakolpour S (2021) Genetics and genomics of SARS-CoV-2: A review of the literature with the special focus on genetic diversity and SARS-CoV-2 genome detection. Genomics 113 (1 Pt 2): 1221-1232 DOI 10.1016/j.ygeno.2020.09.059

3 Hendin HE, Pillet S, Lara AN, Wu CY, Charland N, Landry N, Ward BJ (2017) Plant-made virus-like particle vaccines bearing the hemagglutinin of either seasonal (H1) or avian (H5) influenza have distinct patterns of interaction with human immune cells in vitro. Vaccine 35 (19): 2592-2599 DOI 10.1016/j.vaccine.2017.03.058

4 Makarkov AI, Chierzi S, Pillet S, Murai KK, Landry N, Ward BJ (2017) Plant-made virus-like particles bearing influenza hemagglutinin (HA) recapitulate early interactions of native influenza virions with human monocytes/macrophages. Vaccine 35 (35 Pt B): 4629-4636 DOI 10.1016/j.vaccine.2017.07.012

5 Makarkov AI, Golizeh M, Ruiz-Lancheros E, Gopal AA, Costas-Cancelas IN, Chierzi S, Pillet S, Charland N, Landry N, Rouiller I, Wiseman PW, Ndao M, Ward BJ (2019) Plant-derived virus-like particle vaccines drive cross-presentation of influenza A hemagglutinin peptides by human monocyte-derived macrophages. NPJ Vaccines 4: 17 DOI 10.1038/s41541-019-0111-y

6 Liu J, Dai S, Wang M, Hu Z, Wang H, Deng F (2016) Virus like particle-based vaccines against emerging infectious disease viruses. Virol Sin 31 (4): 279-287 DOI 10.1007/s12250-016-3756-y

7 Moffat JM, Cheong WS, Villadangos JA, Mintern JD, Netter HJ (2013) Hepatitis B virus-like particles access major histocompatibility class I and II antigen presentation pathways in primary dendritic cells. Vaccine 31 (18): 2310-2316 DOI 10.1016/j.vaccine.2013.02.042

8 Lindsay BJ, Bonar MM, Costas-Cancelas IN, Hunt K, Makarkov AI, Chierzi S, Krawczyk CM, Landry N, Ward BJ, Rouiller I (2018) Morphological characterization of a plant-made virus-like particle vaccine bearing influenza virus hemagglutinins by electron microscopy. Vaccine 36 (16): 2147-2154 DOI 10.1016/j.vaccine.2018.02.106

9 Won SY, Hunt K, Guak H, Hasaj B, Charland N, Landry N, Ward BJ, Krawczyk CM (2018) Characterization of the innate stimulatory capacity of plant-derived virus-like particles bearing influenza hemagglutinin. Vaccine 36 (52): 8028-8038 DOI 10.1016/j.vaccine.2018.10.099

10 Francica JR, Flynn BJ, Foulds KE, Noe AT, Werner AP, Moore IN, Gagne M, Johnston TS, Tucker C, Davis RL, Flach B, O’Connell S, Andrew SF, Lamb E, Flebbe DR, Nurmukhambetova ST, Donaldson MM, Todd J-PM, Zhu AL, Atyeo C, Fischinger S, Gorman MJ, Shin S, Edara VV, Floyd K, Lai L, Tylor A, McCarthy E, Lecouturier V, Ruiz S, Berry C, Tibbitts T, Andersen H, Cook A, Dodson A, Pessaint L, Ry AV, Koutsoukos M, Gutzeit C, Teng I-T, Zhou T, Li D, Haynes BF, Kwong PD, McDermott A, Lewis MG, Fu TM, Chicz R, van der Most R, Corbett KS, Suthar MS, Alter G, Roederer M, Sullivan NJ, Douek DC, Graham BS, Casimiro D, Seder RA (2021) Vaccination with SARS-CoV-2 Spike Protein and AS03 Adjuvant Induces Rapid Anamnestic Antibodies in the Lung and Protects Against Virus Challenge in Nonhuman Primates. bioRxiv: 2021.2003.2002.433390 DOI 10.1101/2021.03.02.433390

11 Graham SP, McLean RK, Spencer AJ, Belij-Rammerstorfer S, Wright D, Ulaszewska M, Edwards JC, Hayes JWP, Martini V, Thakur N, Conceicao C, Dietrich I, Shelton H, Waters R, Ludi A, Wilsden G, Browning C, Bialy D, Bhat S, Stevenson-Leggett P, Hollinghurst P, Gilbride C, Pulido D, Moffat K, Sharpe H, Allen E, Mioulet V, Chiu C, Newman J, Asfor AS, Burman A, Crossley S, Huo J, Owens RJ, Carroll M, Hammond JA, Tchilian E, Bailey D, Charleston B, Gilbert SC, Tuthill TJ, Lambe T (2020) Evaluation of the immunogenicity of prime-boost vaccination with the replication-deficient viral vectored COVID-19 vaccine candidate ChAdOx1 nCoV-19. bioRxiv: 2020.2006.2020.159715 DOI 10.1101/2020.06.20.159715

12 van Doremalen N, Lambe T, Spencer A, Belij-Rammerstorfer S, Purushotham JN, Port JR, Avanzato VA, Bushmaker T, Flaxman A, Ulaszewska M, Feldmann F, Allen ER, Sharpe H, Schulz J, Holbrook M, Okumura A, Meade-White K, Pérez-Pérez L, Edwards NJ, Wright D, Bissett C, Gilbride C, Williamson BN, Rosenke R, Long D, Ishwarbhai A, Kailath R, Rose L, Morris S, Powers C, Lovaglio J, Hanley PW, Scott D, Saturday G, de Wit E, Gilbert SC, Munster VJ (2020) ChAdOx1 nCoV-19 vaccine prevents SARS-CoV-2 pneumonia in rhesus macaques. Nature 586 (7830): 578-582 DOI 10.1038/s41586-020-2608-y

13 Vogel AB, Kanevsky I, Che Y, Swanson KA, Muik A, Vormehr M, Kranz LM, Walzer KC, Hein S, Güler A, Loschko J, Maddur MS, Ota-Setlik A, Tompkins K, Cole J, Lui BG, Ziegenhals T, Plaschke A, Eisel D, Dany SC, Fesser S, Erbar S, Bates F, Schneider D, Jesionek B, Sänger B, Wallisch A-K, Feuchter Y, Junginger H, Krumm SA, Heinen AP, Adams-Quack P, Schlereth J, Schille S, Kröner C, de la Caridad Güimil Garcia R, Hiller T, Fischer L, Sellers RS, Choudhary S, Gonzalez O, Vascotto F, Gutman MR, Fontenot JA, Hall-Ursone S, Brasky K, Griffor MC, Han S, Su AAH, Lees JA, Nedoma NL, Mashalidis EH, Sahasrabudhe PV, Tan CY, Pavliakova D, Singh G, Fontes-Garfias C, Pride M, Scully IL, Ciolino T, Obregon J, Gazi M, Carrion R, Alfson KJ, Kalina WV, Kaushal D, Shi P-Y, Klamp T, Rosenbaum C, Kuhn AN, Türeci Ö, Dormitzer PR, Jansen KU, Sahin U (2021) BNT162b vaccines protect rhesus macaques from SARS-CoV-2. Nature DOI 10.1038/s41586-021-03275-y

14 Rauch S, Gooch K, Hall Y, Salguero FJ, Dennis MJ, Gleeson FV, Harris D, Ho C, Humphries HE, Longet S, Ngabo D, Paterson J, Rayner EL, Ryan KA, Sharpe S, Watson RJ, Mueller SO, Petsch B, Carroll MW (2020) mRNA vaccine CVnCoV protects non-human primates from SARS-CoV-2 challenge infection. bioRxiv: 2020.2012.2023.424138 DOI 10.1101/2020.12.23.424138

15 Rauch S, Roth N, Schwendt K, Fotin-Mleczek M, Mueller SO, Petsch B (2021) mRNA based SARS-CoV-2 vaccine candidate CVnCoV induces high levels of virus neutralizing antibodies and mediates protection in rodents. bioRxiv: 2020.2010.2023.351775 DOI 10.1101/2020.10.23.351775

16 Kremsner P, Mann P, Bosch J, Fendel R, Gabor JJ, Kreidenweiss A, Kroidl A, Leroux-Roels I, Leroux-Roels G, Schindler C, Schunk M, Velavan TP, Fotin-Mleczek M, Müller S, Quintini G, Schönborn-Kellenberger O, Vahrenhorst D, Verstraeten T, Walz L, Wolz O-O, Oostvogels L (2020) Phase 1 Assessment of the Safety and Immunogenicity of an mRNA- Lipid Nanoparticle Vaccine Candidate Against SARS-CoV-2 in Human Volunteers. medRxiv: 2020.2011.2009.20228551 DOI 10.1101/2020.11.09.20228551

17 van Els C, Mjaaland S, Naess L, Sarkadi J, Gonczol E, Korsholm KS, Hansen J, de Jonge J, Kersten G, Warner J, Semper A, Kruiswijk C, Oftung F (2014) Fast vaccine design and development based on correlates of protection (COPs). Hum Vaccin Immunother 10 (7): 1935-1948 DOI 10.4161/hv.28639

18 Hodgins B, Pillet S, Landry N, Ward BJ (2019) Prime-pull vaccination with a plant-derived virus-like particle influenza vaccine elicits a broad immune response and protects aged mice from death and frailty after challenge. Immun Ageing 16: 27 DOI 10.1186/s12979-019-0167-6

19 Hodgins B, Pillet S, Landry N, Ward BJ (2019) A plant-derived VLP influenza vaccine elicits a balanced immune response even in very old mice with co-morbidities. PLoS One 14 (1): e0210009 DOI 10.1371/journal.pone.0210009

20 Hodgins B, Yam KK, Winter K, Pillet S, Landry N, Ward BJ (2017) A Single Intramuscular Dose of a Plant-Made Virus-Like Particle Vaccine Elicits a Balanced Humoral and Cellular Response and Protects Young and Aged Mice from Influenza H1N1 Virus Challenge despite a Modest/Absent Humoral Response. Clin Vaccine Immunol 24 (12) DOI 10.1128/CVI.00273-17

21 Vogel AB, Kanevsky I, Che Y, Swanson KA, Muik A, Vormehr M, Kranz LM, Walzer KC, Hein S, Güler A, Loschko J, Maddur MS, Ota-Setlik A, Tompkins K, Cole J, Lui BG, Ziegenhals T, Plaschke A, Eisel D, Dany SC, Fesser S, Erbar S, Bates F, Schneider D, Jesionek B, Sänger B, Wallisch AK, Feuchter Y, Junginger H, Krumm SA, Heinen AP, Adams-Quack P, Schlereth J, Schille S, Kröner C, de la Caridad Güimil Garcia R, Hiller T, Fischer L, Sellers RS, Choudhary S, Gonzalez O, Vascotto F, Gutman MR, Fontenot JA, Hall-Ursone S, Brasky K, Griffor MC, Han S, Su AAH, Lees JA, Nedoma NL, Mashalidis EH, Sahasrabudhe PV, Tan CY, Pavliakova D, Singh G, Fontes-Garfias C, Pride M, Scully IL, Ciolino T, Obregon J, Gazi M, Carrion R, Jr., Alfson KJ, Kalina WV, Kaushal D, Shi PY, Klamp T, Rosenbaum C, Kuhn AN, Türeci Ö, Dormitzer PR, Jansen KU, Sahin U (2021) BNT162b vaccines protect rhesus macaques from SARS-CoV-2. Nature 592 (7853): 283-289 DOI 10.1038/s41586-021-03275-y

22 Muñoz-Fontela C, Dowling WE, Funnell SGP, Gsell P-S, Riveros-Balta AX, Albrecht RA, Andersen H, Baric RS, Carroll MW, Cavaleri M, Qin C, Crozier I, Dallmeier K, de Waal L, de Wit E, Delang L, Dohm E, Duprex WP, Falzarano D, Finch CL, Frieman MB, Graham BS, Gralinski LE, Guilfoyle K, Haagmans BL, Hamilton GA, Hartman AL, Herfst S, Kaptein SJF, Klimstra WB, Knezevic I, Krause PR, Kuhn JH, Le Grand R, Lewis MG, Liu W-C, Maisonnasse P, McElroy AK, Munster V, Oreshkova N, Rasmussen AL, Rocha-Pereira J, Rockx B, Rodríguez E, Rogers TF, Salguero FJ, Schotsaert M, Stittelaar KJ, Thibaut HJ, Tseng C-T, Vergara-Alert J, Beer M, Brasel T, Chan JFW, García-Sastre A, Neyts J, Perlman S, Reed DS, Richt JA, Roy CJ, Segalés J, Vasan SS, Henao-Restrepo AM, Barouch DH (2020) Animal models for COVID-19. Nature 586 (7830): 509-515 DOI 10.1038/s41586-020-2787-6

23 Pandamooz S, Jurek B, Meinung CP, Baharvand Z, Shahem-Abadi AS, Haerteis S, Miyan JA, Downing J, Dianatpour M, Borhani-Haghighi A, Salehi MS (2021) Experimental Models of SARS-CoV-2 Infection: Possible Platforms to Study COVID-19 Pathogenesis and Potential Treatments. Annu Rev Pharmacol Toxicol DOI 10.1146/annurev-pharmtox-121120-012309

24 Pillet S, Racine T, Nfon C, Di Lenardo TZ, Babiuk S, Ward BJ, Kobinger GP, Landry N (2015) Plant-derived H7 VLP vaccine elicits protective immune response against H7N9 influenza virus in mice and ferrets. Vaccine 33 (46): 6282-6289 DOI 10.1016/j.vaccine.2015.09.065

25 Severe Covid GG, Ellinghaus D, Degenhardt F, Bujanda L, Buti M, Albillos A, Invernizzi P, Fernandez J, Prati D, Baselli G, Asselta R, Grimsrud MM, Milani C, Aziz F, Kassens J, May S, Wendorff M, Wienbrandt L, Uellendahl-Werth F, Zheng T, Yi X, de Pablo R, Chercoles AG, Palom A, Garcia-Fernandez AE, Rodriguez-Frias F, Zanella A, Bandera A, Protti A, Aghemo A, Lleo A, Biondi A, Caballero-Garralda A, Gori A, Tanck A, Carreras Nolla A, Latiano A, Fracanzani AL, Peschuck A, Julia A, Pesenti A, Voza A, Jimenez D, Mateos B, Nafria Jimenez B, Quereda C, Paccapelo C, Gassner C, Angelini C, Cea C, Solier A, Pestana D, Muniz-Diaz E, Sandoval E, Paraboschi EM, Navas E, Garcia Sanchez F, Ceriotti F, Martinelli-Boneschi F, Peyvandi F, Blasi F, Tellez L, Blanco-Grau A, Hemmrich-Stanisak G, Grasselli G, Costantino G, Cardamone G, Foti G, Aneli S, Kurihara H, ElAbd H, My I, Galvan-Femenia I, Martin J, Erdmann J, Ferrusquia-Acosta J, Garcia-Etxebarria K, Izquierdo-Sanchez L, Bettini LR, Sumoy L, Terranova L, Moreira L, Santoro L, Scudeller L, Mesonero F, Roade L, Ruhlemann MC, Schaefer M, Carrabba M, Riveiro-Barciela M, Figuera Basso ME, Valsecchi MG, Hernandez-Tejero M, Acosta-Herrera M, D'Angio M, Baldini M, Cazzaniga M, Schulzky M, Cecconi M, Wittig M, Ciccarelli M, Rodriguez-Gandia M, Bocciolone M, Miozzo M, Montano N, Braun N, Sacchi N, Martinez N, Ozer O, Palmieri O, Faverio P, Preatoni P, Bonfanti P, Omodei P, Tentorio P, Castro P, Rodrigues PM, Blandino Ortiz A, de Cid R, Ferrer R, Gualtierotti R, Nieto R, Goerg S, Badalamenti S, Marsal S, Matullo G, Pelusi S, Juzenas S, Aliberti S, Monzani V, Moreno V, Wesse T, Lenz TL, Pumarola T, Rimoldi V, Bosari S, Albrecht W, Peter W, Romero-Gomez M, D'Amato M, Duga S, Banales JM, Hov JR, Folseraas T, Valenti L, Franke A, Karlsen TH (2020) Genomewide Association Study of Severe Covid-19 with Respiratory Failure. N Engl J Med 383 (16): 1522-1534 DOI 10.1056/NEJMoa2020283

26 Walsh EE, Frenck RW, Jr., Falsey AR, Kitchin N, Absalon J, Gurtman A, Lockhart S, Neuzil K, Mulligan MJ, Bailey R, Swanson KA, Li P, Koury K, Kalina W, Cooper D, Fontes-Garfias C, Shi PY, Tureci O, Tompkins KR, Lyke KE, Raabe V, Dormitzer PR, Jansen KU, Sahin U, Gruber WC (2020) Safety and Immunogenicity of Two RNA-Based Covid-19 Vaccine Candidates. N Engl J Med 383 (25): 2439-2450 DOI 10.1056/NEJMoa2027906

27 Lamb YN (2021) BNT162b2 mRNA COVID-19 Vaccine: First Approval. Drugs DOI 10.1007/s40265-021-01480-7

28 Amit S, Regev-Yochay G, Afek A, Kreiss Y, Leshem E (2021) Early rate reductions of SARS-CoV-2 infection and COVID-19 in BNT162b2 vaccine recipients. Lancet 397 (10277): 875-877 DOI 10.1016/S0140-6736(21)00448-7

29 Polack FP, Thomas SJ, Kitchin N, Absalon J, Gurtman A, Lockhart S, Perez JL, Perez Marc G, Moreira ED, Zerbini C, Bailey R, Swanson KA, Roychoudhury S, Koury K, Li P, Kalina WV, Cooper D, Frenck RW, Jr., Hammitt LL, Tureci O, Nell H, Schaefer A, Unal S, Tresnan DB, Mather S, Dormitzer PR, Sahin U, Jansen KU, Gruber WC, Group CCT (2020) Safety and Efficacy of the BNT162b2 mRNA Covid-19 Vaccine. N Engl J Med 383 (27): 2603-2615 DOI 10.1056/NEJMoa2034577

30 Baden LR, El Sahly HM, Essink B, Kotloff K, Frey S, Novak R, Diemert D, Spector SA, Rouphael N, Creech CB, McGettigan J, Khetan S, Segall N, Solis J, Brosz A, Fierro C, Schwartz H, Neuzil K, Corey L, Gilbert P, Janes H, Follmann D, Marovich M, Mascola J, Polakowski L, Ledgerwood J, Graham BS, Bennett H, Pajon R, Knightly C, Leav B, Deng W, Zhou H, Han S, Ivarsson M, Miller J, Zaks T, Group CS (2021) Efficacy and Safety of the mRNA-1273 SARS-CoV-2 Vaccine. N Engl J Med 384 (5): 403-416 DOI 10.1056/NEJMoa2035389

31 Aldrich C, Leroux-Roels I, Huang KB, Bica MA, Loeliger E, Schoenborn-Kellenberger O, Walz L, Leroux-Roels G, von Sonnenburg F, Oostvogels L (2021) Proof-of-concept of a low-dose unmodified mRNA-based rabies vaccine formulated with lipid nanoparticles in human volunteers: A phase 1 trial. Vaccine 39 (8): 1310-1318 DOI 10.1016/j.vaccine.2020.12.070

32 Grabbe S, Haas H, Diken M, Kranz LM, Langguth P, Sahin U (2016) Translating nanoparticulate-personalized cancer vaccines into clinical applications: case study with RNA-lipoplexes for the treatment of melanoma. Nanomedicine (Lond) 11 (20): 2723-2734 DOI 10.2217/nnm-2016-0275

33 Mohsen MO, Zha L, Cabral-Miranda G, Bachmann MF (2017) Major findings and recent advances in virus-like particle (VLP)-based vaccines. Semin Immunol 34: 123-132 DOI 10.1016/j.smim.2017.08.014

34 Mor TS (2015) Molecular pharming's foot in the FDA's door: Protalix's trailblazing story. Biotechnol Lett 37 (11): 2147-2150 DOI 10.1007/s10529-015-1908-z

35 Ward BJ, Makarkov A, Seguin A, Pillet S, Trepanier S, Dhaliwall J, Libman MD, Vesikari T, Landry N (2020) Efficacy, immunogenicity, and safety of a plant-derived, quadrivalent, virus-like particle influenza vaccine in adults (18-64 years) and older adults (>/=65 years): two multicentre, randomised phase 3 trials. Lancet 396 (10261): 1491-1503 DOI 10.1016/S0140-6736(20)32014-6

36 Ward BJ, Seguin A, Couillard J, Trepanier S, Landry N (2021) Phase III: Randomized observer-blind trial to evaluate lot-to-lot consistency of a new plant-derived quadrivalent virus like particle influenza vaccine in adults 18-49 years of age. Vaccine 39 (10): 1528-1533 DOI 10.1016/j.vaccine.2021.01.004

37 Pillet S, Aubin E, Trepanier S, Bussiere D, Dargis M, Poulin JF, Yassine-Diab B, Ward BJ, Landry N (2016) A plant-derived quadrivalent virus like particle influenza vaccine induces cross-reactive antibody and T cell response in healthy adults. Clin Immunol 168: 72-87 DOI 10.1016/j.clim.2016.03.008

38 Pillet S, Couillard J, Trepanier S, Poulin JF, Yassine-Diab B, Guy B, Ward BJ, Landry N (2019) Immunogenicity and safety of a quadrivalent plant-derived virus like particle influenza vaccine candidate-Two randomized Phase II clinical trials in 18 to 49 and >/=50 years old adults. PLoS One 14 (6): e0216533 DOI 10.1371/journal.pone.0216533

39 Heath PT, Galiza EP, Baxter DN, Boffito M, Browne D, Burns F, Chadwick DR, Clark R, Cosgrove C, Galloway J, Goodman AL, Heer A, Higham A, Iyengar S, Jamal A, Jeanes C, Kalra PA, Kyriakidou C, McAuley DF, Meyrick A, Minassian AM, Minton J, Moore P, Munsoor I, Nicholls H, Osanlou O, Packham J, Pretswell CH, Francisco Ramos AS, Saralaya D, Sheridan RP, Smith R, Soiza RL, Swift PA, Thomson EC, Turner J, Viljoen ME, Albert G, Cho I, Dubovsky F, Glenn G, Rivers J, Robertson A, Smith K, Toback S (2021) Efficacy of the NVX-CoV2373 Covid-19 Vaccine Against the B.1.1.7 Variant. medRxiv: 2021.2005.2013.21256639 DOI 10.1101/2021.05.13.21256639

40 Keech C, Albert G, Cho I, Robertson A, Reed P, Neal S, Plested JS, Zhu M, Cloney-Clark S, Zhou H, Smith G, Patel N, Frieman MB, Haupt RE, Logue J, McGrath M, Weston S, Piedra PA, Desai C, Callahan K, Lewis M, Price-Abbott P, Formica N, Shinde V, Fries L, Lickliter JD, Griffin P, Wilkinson B, Glenn GM (2020) Phase 1-2 Trial of a SARS-CoV-2 Recombinant Spike Protein Nanoparticle Vaccine. N Engl J Med 383 (24): 2320-2332 DOI 10.1056/NEJMoa2026920

41 Dunkle LM, Izikson R, Patriarca P, Goldenthal KL, Muse D, Callahan J, Cox MMJ, Team PSCS (2017) Efficacy of Recombinant Influenza Vaccine in Adults 50 Years of Age or Older. N Engl J Med 376 (25): 2427-2436 DOI 10.1056/NEJMoa1608862

42 Dunkle LM, Izikson R, Patriarca PA, Goldenthal KL, Muse D, Cox MMJ (2017) Randomized Comparison of Immunogenicity and Safety of Quadrivalent Recombinant Versus Inactivated Influenza Vaccine in Healthy Adults 18-49 Years of Age. J Infect Dis 216 (10): 1219-1226 DOI 10.1093/infdis/jix478

43 Dunkle LM, Izikson R, Patriarca PA, Goldenthal KL, Cox M, Treanor JJ (2018) Safety and Immunogenicity of a Recombinant Influenza Vaccine: A Randomized Trial. Pediatrics 141 (5) DOI 10.1542/peds.2017-3021

44 Cunningham AL, Lal H, Kovac M, Chlibek R, Hwang SJ, Diez-Domingo J, Godeaux O, Levin MJ, McElhaney JE, Puig-Barbera J, Vanden Abeele C, Vesikari T, Watanabe D, Zahaf T, Ahonen A, Athan E, Barba-Gomez JF, Campora L, de Looze F, Downey HJ, Ghesquiere W, Gorfinkel I, Korhonen T, Leung E, McNeil SA, Oostvogels L, Rombo L, Smetana J, Weckx L, Yeo W, Heineman TC, Group ZOES (2016) Efficacy of the Herpes Zoster Subunit Vaccine in Adults 70 Years of Age or Older. N Engl J Med 375 (11): 1019-1032 DOI 10.1056/NEJMoa1603800

45 Chen DS (2009) Hepatitis B vaccination: The key towards elimination and eradication of hepatitis B. J Hepatol 50 (4): 805-816 DOI 10.1016/j.jhep.2009.01.002

46 Wheeler CM, Skinner SR, Del Rosario-Raymundo MR, Garland SM, Chatterjee A, Lazcano-Ponce E, Salmeron J, McNeil S, Stapleton JT, Bouchard C, Martens MG, Money DM, Quek SC, Romanowski B, Vallejos CS, Ter Harmsel B, Prilepskaya V, Fong KL, Kitchener H, Minkina G, Lim YKT, Stoney T, Chakhtoura N, Cruickshank ME, Savicheva A, da Silva DP, Ferguson M, Molijn AC, Quint WGV, Hardt K, Descamps D, Suryakiran PV, Karkada N, Geeraerts B, Dubin G, Struyf F, Group VS (2016) Efficacy, safety, and immunogenicity of the human papillomavirus 16/18 AS04-adjuvanted vaccine in women older than 25 years: 7-year follow-up of the phase 3, double-blind, randomised controlled VIVIANE study. Lancet Infect Dis 16 (10): 1154-1168 DOI 10.1016/S1473-3099(16)30120-7

47 van Doremalen N, Haddock E, Feldmann F, Meade-White K, Bushmaker T, Fischer RJ, Okumura A, Hanley PW, Saturday G, Edwards NJ, Clark MHA, Lambe T, Gilbert SC, Munster VJ (2020) A single dose of ChAdOx1 MERS provides protective immunity in rhesus macaques. Sci Adv 6 (24): eaba8399 DOI 10.1126/sciadv.aba8399

48 Alberer M, Gnad-Vogt U, Hong HS, Mehr KT, Backert L, Finak G, Gottardo R, Bica MA, Garofano A, Koch SD, Fotin-Mleczek M, Hoerr I, Clemens R, von Sonnenburg F (2017) Safety and immunogenicity of a mRNA rabies vaccine in healthy adults: an open-label, non-randomised, prospective, first-in-human phase 1 clinical trial. Lancet 390 (10101): 1511-1520 DOI 10.1016/s0140-6736(17)31665-3

49 Feldman RA, Fuhr R, Smolenov I, Mick Ribeiro A, Panther L, Watson M, Senn JJ, Smith M, Almarsson Ӧ, Pujar HS, Laska ME, Thompson J, Zaks T, Ciaramella G (2019) mRNA vaccines against H10N8 and H7N9 influenza viruses of pandemic potential are immunogenic and well tolerated in healthy adults in phase 1 randomized clinical trials. Vaccine 37 (25): 3326-3334 DOI 10.1016/j.vaccine.2019.04.074

50 Kranz LM, Diken M, Haas H, Kreiter S, Loquai C, Reuter KC, Meng M, Fritz D, Vascotto F, Hefesha H, Grunwitz C, Vormehr M, Hüsemann Y, Selmi A, Kuhn AN, Buck J, Derhovanessian E, Rae R, Attig S, Diekmann J, Jabulowsky RA, Heesch S, Hassel J, Langguth P, Grabbe S, Huber C, Türeci Ö, Sahin U (2016) Systemic RNA delivery to dendritic cells exploits antiviral defence for cancer immunotherapy. Nature 534 (7607): 396-401 DOI 10.1038/nature18300

51 Sahin U, Derhovanessian E, Miller M, Kloke BP, Simon P, Löwer M, Bukur V, Tadmor AD, Luxemburger U, Schrörs B, Omokoko T, Vormehr M, Albrecht C, Paruzynski A, Kuhn AN, Buck J, Heesch S, Schreeb KH, Müller F, Ortseifer I, Vogler I, Godehardt E, Attig S, Rae R, Breitkreuz A, Tolliver C, Suchan M, Martic G, Hohberger A, Sorn P, Diekmann J, Ciesla J, Waksmann O, Brück AK, Witt M, Zillgen M, Rothermel A, Kasemann B, Langer D, Bolte S, Diken M, Kreiter S, Nemecek R, Gebhardt C, Grabbe S, Höller C, Utikal J, Huber C, Loquai C, Türeci Ö (2017) Personalized RNA mutanome vaccines mobilize poly-specific therapeutic immunity against cancer. Nature 547 (7662): 222-226 DOI 10.1038/nature23003

52 Jackson LA, Anderson EJ, Rouphael NG, Roberts PC, Makhene M, Coler RN, McCullough MP, Chappell JD, Denison MR, Stevens LJ, Pruijssers AJ, McDermott A, Flach B, Doria-Rose NA, Corbett KS, Morabito KM, O'Dell S, Schmidt SD, Swanson PA, 2nd, Padilla M, Mascola JR, Neuzil KM, Bennett H, Sun W, Peters E, Makowski M, Albert J, Cross K, Buchanan W, Pikaart-Tautges R, Ledgerwood JE, Graham BS, Beigel JH, m RNASG (2020) An mRNA Vaccine against SARS-CoV-2 - Preliminary Report. N Engl J Med 383 (20): 1920-1931 DOI 10.1056/NEJMoa2022483

53 Ward BJ, Gobeil P, Séguin A, Atkins J, Boulay I, Charbonneau P-Y, Couture M, D’Aoust M-A, Dhaliwall J, Finkle C, Hager K, Mahmood A, Makarkov A, Cheng M, Pillet S, Schimke P, St-Martin S, Trépanier S, Landry N (2020) Phase 1 trial of a Candidate Recombinant Virus-Like Particle Vaccine for Covid-19 Disease Produced in Plants. medRxiv: 2020.2011.2004.20226282 DOI 10.1101/2020.11.04.20226282

54 Gobeil P, Pillet S, Séguin A, Boulay I, Mahmood A, Vinh DC, Charland N, Boutet P, Roman FP, Van Der Most R, de los Angeles Ceregido Perez M, Ward BJ, Landry N (2021) Interim Report of a Phase 2 Randomized Trial of a Plant-Produced Virus-Like Particle Vaccine for Covid-19 in Healthy Adults Aged 18-64 and Older Adults Aged 65 and Older. medRxiv: 2021.2005.2014.21257248 DOI 10.1101/2021.05.14.21257248

55 Goepfert PA, Fu B, Chabanon AL, Bonaparte MI, Davis MG, Essink BJ, Frank I, Haney O, Janosczyk H, Keefer MC, Koutsoukos M, Kimmel MA, Masotti R, Savarino SJ, Schuerman L, Schwartz H, Sher LD, Smith J, Tavares-Da-Silva F, Gurunathan S, DiazGranados CA, de Bruyn G (2021) Safety and immunogenicity of SARS-CoV-2 recombinant protein vaccine formulations in healthy adults: interim results of a randomised, placebo-controlled, phase 1-2, dose-ranging study. Lancet Infect Dis DOI 10.1016/S1473-3099(21)00147-X

56 Sridhar S, Arnel J, Bonaparte MI, Bueso A, Chabanon A-L, Chen A, Chicz RM, Diemert D, Essink BJ, Fu B, Grunenberg NA, Janosczyk H, Keefer MC, Rivera M DM, Meng Y, Michael NL, Munsiff SS, Ogbuagu O, Raabe VN, Severance R, Rivas E, Romanyak N, Rouphael NG, Schuerman L, Sher LD, Walsh SR, White J, von Barbier D, de Bruyn G, Canter R, Grillet M-H, Keshtkar-Jahromi M, Koutsoukos M, Lopez D, Masotti R, Mendoza S, Moreau C, Ceregido MA, Ramirez S, Said A, Tavares-Da-Silva F, Shi J, Tong T, Treanor J, Diazgranados CA, Savarino S (2021) Safety and immunogenicity of a SARS-CoV-2 recombinant protein vaccine with AS03 adjuvant in healthy adults: interim findings from a phase 2, randomised, dose-finding, multi-centre study. medRxiv: 2021.2010.2008.21264302 DOI 10.1101/2021.10.08.21264302

57 Folegatti PM, Ewer KJ, Aley PK, Angus B, Becker S, Belij-Rammerstorfer S, Bellamy D, Bibi S, Bittaye M, Clutterbuck EA, Dold C, Faust SN, Finn A, Flaxman AL, Hallis B, Heath P, Jenkin D, Lazarus R, Makinson R, Minassian AM, Pollock KM, Ramasamy M, Robinson H, Snape M, Tarrant R, Voysey M, Green C, Douglas AD, Hill AVS, Lambe T, Gilbert SC, Pollard AJ (2020) Safety and immunogenicity of the ChAdOx1 nCoV-19 vaccine against SARS-CoV-2: a preliminary report of a phase 1/2, single-blind, randomised controlled trial. Lancet 396 (10249): 467-478 DOI 10.1016/s0140-6736(20)31604-4

58 Ramasamy MN, Minassian AM, Ewer KJ, Flaxman AL, Folegatti PM, Owens DR, Voysey M, Aley PK, Angus B, Babbage G, Belij-Rammerstorfer S, Berry L, Bibi S, Bittaye M, Cathie K, Chappell H, Charlton S, Cicconi P, Clutterbuck EA, Colin-Jones R, Dold C, Emary KRW, Fedosyuk S, Fuskova M, Gbesemete D, Green C, Hallis B, Hou MM, Jenkin D, Joe CCD, Kelly EJ, Kerridge S, Lawrie AM, Lelliott A, Lwin MN, Makinson R, Marchevsky NG, Mujadidi Y, Munro APS, Pacurar M, Plested E, Rand J, Rawlinson T, Rhead S, Robinson H, Ritchie AJ, Ross-Russell AL, Saich S, Singh N, Smith CC, Snape MD, Song R, Tarrant R, Themistocleous Y, Thomas KM, Villafana TL, Warren SC, Watson MEE, Douglas AD, Hill AVS, Lambe T, Gilbert SC, Faust SN, Pollard AJ (2021) Safety and immunogenicity of ChAdOx1 nCoV-19 vaccine administered in a prime-boost regimen in young and old adults (COV002): a single-blind, randomised, controlled, phase 2/3 trial. Lancet 396 (10267): 1979-1993 DOI 10.1016/s0140-6736(20)32466-1

59 Voysey M, Clemens SAC, Madhi SA, Weckx LY, Folegatti PM, Aley PK, Angus B, Baillie VL, Barnabas SL, Bhorat QE, Bibi S, Briner C, Cicconi P, Collins AM, Colin-Jones R, Cutland CL, Darton TC, Dheda K, Duncan CJA, Emary KRW, Ewer KJ, Fairlie L, Faust SN, Feng S, Ferreira DM, Finn A, Goodman AL, Green CM, Green CA, Heath PT, Hill C, Hill H, Hirsch I, Hodgson SHC, Izu A, Jackson S, Jenkin D, Joe CCD, Kerridge S, Koen A, Kwatra G, Lazarus R, Lawrie AM, Lelliott A, Libri V, Lillie PJ, Mallory R, Mendes AVA, Milan EP, Minassian AM, McGregor A, Morrison H, Mujadidi YF, Nana A, O'Reilly PJ, Padayachee SD, Pittella A, Plested E, Pollock KM, Ramasamy MN, Rhead S, Schwarzbold AV, Singh N, Smith A, Song R, Snape MD, Sprinz E, Sutherland RK, Tarrant R, Thomson EC, Török ME, Toshner M, Turner DPJ, Vekemans J, Villafana TL, Watson MEE, Williams CJ, Douglas AD, Hill AVS, Lambe T, Gilbert SC, Pollard AJ (2021) Safety and efficacy of the ChAdOx1 nCoV-19 vaccine (AZD1222) against SARS-CoV-2: an interim analysis of four randomised controlled trials in Brazil, South Africa, and the UK. Lancet 397 (10269): 99-111 DOI 10.1016/s0140-6736(20)32661-1

60 Barrett JR, Belij-Rammerstorfer S, Dold C, Ewer KJ, Folegatti PM, Gilbride C, Halkerston R, Hill J, Jenkin D, Stockdale L, Verheul MK, Aley PK, Angus B, Bellamy D, Berrie E, Bibi S, Bittaye M, Carroll MW, Cavell B, Clutterbuck EA, Edwards N, Flaxman A, Fuskova M, Gorringe A, Hallis B, Kerridge S, Lawrie AM, Linder A, Liu X, Madhavan M, Makinson R, Mellors J, Minassian A, Moore M, Mujadidi Y, Plested E, Poulton I, Ramasamy MN, Robinson H, Rollier CS, Song R, Snape MD, Tarrant R, Taylor S, Thomas KM, Voysey M, Watson MEE, Wright D, Douglas AD, Green CM, Hill AVS, Lambe T, Gilbert S, Pollard AJ (2021) Phase 1/2 trial of SARS-CoV-2 vaccine ChAdOx1 nCoV-19 with a booster dose induces multifunctional antibody responses. Nat Med 27 (2): 279-288 DOI 10.1038/s41591-020-01179-4

61 Sahin U, Muik A, Vogler I, Derhovanessian E, Kranz LM, Vormehr M, Quandt J, Bidmon N, Ulges A, Baum A, Pascal KE, Maurus D, Brachtendorf S, Lörks V, Sikorski J, Koch P, Hilker R, Becker D, Eller AK, Grützner J, Tonigold M, Boesler C, Rosenbaum C, Heesen L, Kühnle MC, Poran A, Dong JZ, Luxemburger U, Kemmer-Brück A, Langer D, Bexon M, Bolte S, Palanche T, Schultz A, Baumann S, Mahiny AJ, Boros G, Reinholz J, Szabó GT, Karikó K, Shi PY, Fontes-Garfias C, Perez JL, Cutler M, Cooper D, Kyratsous CA, Dormitzer PR, Jansen KU, Türeci Ö (2021) BNT162b2 vaccine induces neutralizing antibodies and poly-specific T cells in humans. Nature 595 (7868): 572-577 DOI 10.1038/s41586-021-03653-6

62 Mulligan MJ, Lyke KE, Kitchin N, Absalon J, Gurtman A, Lockhart S, Neuzil K, Raabe V, Bailey R, Swanson KA, Li P, Koury K, Kalina W, Cooper D, Fontes-Garfias C, Shi PY, Türeci Ö, Tompkins KR, Walsh EE, Frenck R, Falsey AR, Dormitzer PR, Gruber WC, Şahin U, Jansen KU (2020) Phase I/II study of COVID-19 RNA vaccine BNT162b1 in adults. Nature 586 (7830): 589-593 DOI 10.1038/s41586-020-2639-4

63 Sahin U, Muik A, Derhovanessian E, Vogler I, Kranz LM, Vormehr M, Baum A, Pascal K, Quandt J, Maurus D, Brachtendorf S, Lörks V, Sikorski J, Hilker R, Becker D, Eller AK, Grützner J, Boesler C, Rosenbaum C, Kühnle MC, Luxemburger U, Kemmer-Brück A, Langer D, Bexon M, Bolte S, Karikó K, Palanche T, Fischer B, Schultz A, Shi PY, Fontes-Garfias C, Perez JL, Swanson KA, Loschko J, Scully IL, Cutler M, Kalina W, Kyratsous CA, Cooper D, Dormitzer PR, Jansen KU, Türeci Ö (2020) COVID-19 vaccine BNT162b1 elicits human antibody and T(H)1 T cell responses. Nature 586 (7830): 594-599 DOI 10.1038/s41586-020-2814-7

64 Karthik K, Senthilkumar TMA, Udhayavel S, Raj GD (2020) Role of antibody-dependent enhancement (ADE) in the virulence of SARS-CoV-2 and its mitigation strategies for the development of vaccines and immunotherapies to counter COVID-19. Human Vaccines & Immunotherapeutics 16 (12): 3055-3060 DOI 10.1080/21645515.2020.1796425

65 McElhaney JE, Xie D, Hager WD, Barry MB, Wang Y, Kleppinger A, Ewen C, Kane KP, Bleackley RC (2006) T cell responses are better correlates of vaccine protection in the elderly. J Immunol 176 (10): 6333-6339 DOI 10.4049/jimmunol.176.10.6333

66 McKinstry KK, Dutton RW, Swain SL, Strutt TM (2013) Memory CD4 T cell-mediated immunity against influenza A virus: more than a little helpful. Arch Immunol Ther Exp (Warsz) 61 (5): 341-353 DOI 10.1007/s00005-013-0236-z

67 Lau EHY, Tsang OTY, Hui DSC, Kwan MYW, Chan W-h, Chiu SS, Ko RLW, Chan KH, Cheng SMS, Perera RAPM, Cowling BJ, Poon LLM, Peiris M (2021) Neutralizing antibody titres in SARS-CoV-2 infections. Nature Communications 12 (1): 63 DOI 10.1038/s41467-020-20247-4

68 Addetia A, Crawford KHD, Dingens A, Zhu H, Roychoudhury P, Huang ML, Jerome KR, Bloom JD, Greninger AL (2020) Neutralizing Antibodies Correlate with Protection from SARS-CoV-2 in Humans during a Fishery Vessel Outbreak with a High Attack Rate. J Clin Microbiol 58 (11) DOI 10.1128/JCM.02107-20

69 Lipsitch M, Dean NE (2020) Understanding COVID-19 vaccine efficacy. Science 370 (6518): 763-765 DOI 10.1126/science.abe5938

70 Lin DY, Zeng D, Mehrotra DV, Corey L, Gilbert PB (2020) Evaluating the Efficacy of COVID-19 Vaccines. Clin Infect Dis DOI 10.1093/cid/ciaa1863

71 Xing K, Tu XY, Liu M, Liang ZW, Chen JN, Li JJ, Jiang LG, Xing FQ, Jiang Y (2021) Efficacy and safety of COVID-19 vaccines: a systematic review. Zhongguo Dang Dai Er Ke Za Zhi 23 (3): 221-228

72 Krause P, Fleming TR, Longini I, Henao-Restrepo AM, Peto R, World Health Organization Solidarity Vaccines Trial Expert G (2020) COVID-19 vaccine trials should seek worthwhile efficacy. Lancet 396 (10253): 741-743 DOI 10.1016/S0140-6736(20)31821-3

73 Kim JH, Marks F, Clemens JD (2021) Looking beyond COVID-19 vaccine phase 3 trials. Nature Medicine 27 (2): 205-211 DOI 10.1038/s41591-021-01230-y

74 Kyriakidis NC, Lopez-Cortes A, Gonzalez EV, Grimaldos AB, Prado EO (2021) SARS-CoV-2 vaccines strategies: a comprehensive review of phase 3 candidates. NPJ Vaccines 6 (1): 28 DOI 10.1038/s41541-021-00292-w

75 Neumann-Podczaska A, Al-Saad SR, Karbowski LM, Chojnicki M, Tobis S, Wieczorowska-Tobis K (2020) COVID 19 - Clinical Picture in the Elderly Population: A Qualitative Systematic Review. Aging Dis 11 (4): 988-1008 DOI 10.14336/AD.2020.0620

76 Rauch S, Jasny E, Schmidt KE, Petsch B (2018) New Vaccine Technologies to Combat Outbreak Situations. Front Immunol 9: 1963 DOI 10.3389/fimmu.2018.01963

77 Omersel J, Karas Kuzelicki N (2020) Vaccinomics and Adversomics in the Era of Precision Medicine: A Review Based on HBV, MMR, HPV, and COVID-19 Vaccines. J Clin Med 9 (11) DOI 10.3390/jcm9113561
