# Supplementary material for: Translatability scoring in prospective and retrospective COVID drug development cases
Source: Eur J Clin Pharmacol. 2023 Jun 6;79(8):1051–71. doi: 10.1007/s00228-023-03517-0 (PMC10243273; doi:10.1007/s00228-023-03517-0)
Supplement: Supplementary file 2 — Supplementary file2 (DOCX 338 kb) [file 228_2023_3517_MOESM2_ESM.docx]

**Supplementary table 2: Translatability Scores for drugs to treat COVID-19:**

|  |  | **prospective** | | | **retrospective** | | | | |
| --- | --- | --- | --- | --- | --- | --- | --- | --- | --- |
| **Data Lock** |  | **7.5.2021** | **25.6.21** | **17.6.2021** | **28.4.2020** | **04.5.2020** |  | **28.7.2020** | **11.4.2021** |
| **Compound** |  | **camostat mesylate** | **molnupiravir** | **fluvoxamine** | **remdesivir** | **hydroxy**  **chloroquine** |  | **tocilizumab** | **dapagliflozin** |
|  | Weight (%) | Score x weight/100 |  |  |  |  | Weight (%) original score |  |  |
| ***Aspect*** |  |  |  |  |  |  |  |  |  |
| **Starting evidence** |  |  |  |  |  |  |  |  |  |
| *In vitro* data including animal genetics | 9 | 0.27 [1-8] | 0.27 [9-15] | 0.18 [16-19] [20, 21] [22] [23-26] | 0.45 [27] [28-33] | 0.45 [34-37] | 2 | 0.04 [38-40] |  |
| *In vivo* data including animal genetics | 1 | 0.04 [41-46] | 0.05 [10, 11, 15, 47-51] | 0.02 [52] [53] [18, 54] | 0.04 [31, 32, 55-57] | 0.03 [58] | 3 | 0.09 [59] |  |
| Animal disease models | 1 | 0.02 [41-46] | 0.03 [10, 15, 48] | 0.01 | 0.03 [60] | 0.01 | 3 | 0.06 [61, 62] | 0.03 |
| Data from multiple species | 1 | 0.02 [41-46] | 0.05 [10, 11, 15, 47-51] | 0.01 | 0.05 [31, 32, 55, 56, 63] | 0.03 [34, 58] | 3 | 0.03 [59] | 0.03 |
| **Human evidence** |  |  |  |  |  |  |  |  |  |
| Genetics | 4 | 0.12 [41, 64-67] | 0.04 | 0.08 [68] | 0.04 | 0.04 | 5 | 0.15 [69] |  |
| Model compounds | 12 | 0.12 [70] | 0.24 [71-78] | 0.12 [79, 80] | 0.12 [81, 82] |  | 13 | 0.39 [83] |  |
| Clinical trials | 12 | 0.12 [84, 85] | 0.36 [72-78] [86, 87] | 0.24 [88] [89] | 0.24 [63, 90, 91] | 0 [92-103] stopper due to hints on increased mortality in the treatment group | 13 | 0.39 [104-129] |  |
| **Biomarkers for efficacy and safety prediction** |  |  |  |  |  |  |  |  |  |
| Biomarker grading | 23 | 0.69 | 0.92 | 0.69 | 0.69 | 0.92 | 24 | 1.2 | 1.20 |
| Biomarker development | 12 | 0.12 [2] | 0.12 [13, 14, 71, 86] | 0.12 [88, 89] | 0.12 [73] | 0.24 [34] | 13 | 0.52 [130] | 0.13 |
| **Proof-of-mechanism, proof-of-principle**  **and proof of concept testing** |  |  |  |  |  |  |  |  |  |
| Biomarker strategy | 5 | 0.1 [131] | 0.15 [71, 86] | 0.05 [88, 89] | 0.05 | 0.05 | 5 | 0.15 [132, 133] | 0.05 |
| Surrogate or endpoint strategy | 7 | 0.14 NCT04652765, NCT04353284, NCT04470544, NCT04435015 NCT04608266, NCT04524663, NCT04455815, NCT04750759, NCT04730206, NCT04625114, NCT04321096, NCT04662073, NCT04530617, NCT04681430, NCT04644705, NCT04657497, NCT04782505, NCT04662086, NCT04374019, NCT04518410, NCT04721535, NCT04521296, NCT04713176 | 0.14 NCT04575597, NCT04405570, NCT04746183, NCT04405739)  [71, 86] | 0.14 NCT04718480, NCT04342663, NCT04668950, NCT04727424, NCT04510194 [88, 89] | 0.28 (clinicaltrials.gov NCT04280705) | 0.21 [100, 101, 103] | 8 | 0.4 [104-129] | 0.32  NCT04350593  NCT04505774  [134] |
| **Personalized medicine aspects** |  |  |  |  |  |  |  |  |  |
| Disease sub-classification and responder concentration | 9 | 0.18 [2, 135-137] | 0.27 [138] | 0.09 | 0.27 [139] | 0.09 | 3 | 0.09 [121, 122, 124] | 0.09 [134] |
| Pharmacogenetics | 4 | 0.04 [67, 131, 140-142] | 0.04 [12] | 0.12 [143] | 0.12 [144] | 0.08 [145, 146] | 5 | 0.1 [147] | 0.1 [148, 149] |
| **Sum** | **100** | **1.98** | **2.68** | **1.87** | **2.50** | **2.15** | **100** | **3.61** | **1.95** |

**References:**

1 Suzuki T, Itoh Y, Sakai Y, Saito A, Okuzaki D, Motooka D, Minami S, Kobayashi T, Yamamoto T, Okamoto T, Takayama K (2020) Generation of human bronchial organoids for SARS-CoV-2 research. bioRxiv: 2020.2005.2025.115600 DOI 10.1101/2020.05.25.115600

2 Hoffmann M, Hofmann-Winkler H, Smith JC, Kruger N, Sorensen LK, Sogaard OS, Hasselstrom JB, Winkler M, Hempel T, Raich L, Olsson S, Yamazoe T, Yamatsuta K, Mizuno H, Ludwig S, Noe F, Sheltzer JM, Kjolby M, Pohlmann S (2020) Camostat mesylate inhibits SARS-CoV-2 activation by TMPRSS2-related proteases and its metabolite GBPA exerts antiviral activity. bioRxiv DOI 10.1101/2020.08.05.237651

3 Hoffmann M, Kleine-Weber H, Schroeder S, Kruger N, Herrler T, Erichsen S, Schiergens TS, Herrler G, Wu NH, Nitsche A, Muller MA, Drosten C, Pohlmann S (2020) SARS-CoV-2 Cell Entry Depends on ACE2 and TMPRSS2 and Is Blocked by a Clinically Proven Protease Inhibitor. Cell 181 (2): 271-280 e278 DOI 10.1016/j.cell.2020.02.052

4 Bestle D, Heindl MR, Limburg H, Van Lam van T, Pilgram O, Moulton H, Stein DA, Hardes K, Eickmann M, Dolnik O, Rohde C, Klenk HD, Garten W, Steinmetzer T, Bottcher-Friebertshauser E (2020) TMPRSS2 and furin are both essential for proteolytic activation of SARS-CoV-2 in human airway cells. Life Sci Alliance 3 (9) DOI 10.26508/lsa.202000786

5 Hempel T, Raich L, Olsson S, Azouz NP, Klingler AM, Rothenberg ME, Noé F (2020) Molecular mechanism of SARS-CoV-2 cell entry inhibition via TMPRSS2 by Camostat and Nafamostat mesylate. bioRxiv: 2020.2007.2021.214098 DOI 10.1101/2020.07.21.214098

6 Breining P, Frolund AL, Hojen JF, Gunst JD, Staerke NB, Saedder E, Cases-Thomas M, Little P, Nielsen LP, Sogaard OS, Kjolby M (2021) Camostat mesylate against SARS-CoV-2 and COVID-19-Rationale, dosing and safety. Basic Clin Pharmacol Toxicol 128 (2): 204-212 DOI 10.1111/bcpt.13533

7 Shang J, Wan Y, Luo C, Ye G, Geng Q, Auerbach A, Li F (2020) Cell entry mechanisms of SARS-CoV-2. Proc Natl Acad Sci U S A 117 (21): 11727-11734 DOI 10.1073/pnas.2003138117

8 Mykytyn AZ, Breugem TI, Riesebosch S, Schipper D, van den Doel PB, Rottier RJ, Lamers MM, Haagmans BL (2020) The SARS-CoV-2 multibasic cleavage site facilitates early serine protease-mediated entry into organoid-derived human airway cells. bioRxiv: 2020.2009.2007.286120 DOI 10.1101/2020.09.07.286120

9 Dan Do TN, Donckers K, Vangeel L, Chatterjee AK, Gallay PA, Bobardt MD, Bilello JP, Cihlar T, De Jonghe S, Neyts J, Jochmans D (2021) A robust SARS-CoV-2 replication model in primary human epithelial cells at the air liquid interface to assess antiviral agents. bioRxiv: 2021.2003.2025.436907 DOI 10.1101/2021.03.25.436907

10 Cox RM, Wolf JD, Plemper RK (2021) Therapeutically administered ribonucleoside analogue MK-4482/EIDD-2801 blocks SARS-CoV-2 transmission in ferrets. Nat Microbiol 6 (1): 11-18 DOI 10.1038/s41564-020-00835-2

11 Rosenke K, Hansen F, Schwarz B, Feldmann F, Haddock E, Rosenke R, Meade-White K, Okumura A, Leventhal S, Hawman DW, Ricotta E, Bosio CM, Saturday G, Feldmann H, Jarvis MA (2020) Orally delivered MK-4482 inhibits SARS-CoV-2 replication in the Syrian hamster model. Res Sq DOI 10.21203/rs.3.rs-86289/v1

12 Zhou S, Hill CS, Sarkar S, Tse LV, Woodburn BMD, Schinazi RF, Sheahan TP, Baric RS, Heise MT, Swanstrom R (2021) beta-D-N 4-hydroxycytidine (NHC) Inhibits SARS-CoV-2 Through Lethal Mutagenesis But Is Also Mutagenic To Mammalian Cells. J Infect Dis DOI 10.1093/infdis/jiab247

13 Gordon CJ, Tchesnokov EP, Schinazi RF, Gotte M (2021) Molnupiravir promotes SARS-CoV-2 mutagenesis via the RNA template. J Biol Chem: 100770 DOI 10.1016/j.jbc.2021.100770

14 Kabinger F, Stiller C, Schmitzová J, Dienemann C, Hillen HS, Höbartner C, Cramer P (2021) Mechanism of molnupiravir-induced SARS-CoV-2 mutagenesis. bioRxiv: 2021.2005.2011.443555 DOI 10.1101/2021.05.11.443555

15 Yoon JJ, Toots M, Lee S, Lee ME, Ludeke B, Luczo JM, Ganti K, Cox RM, Sticher ZM, Edpuganti V, Mitchell DG, Lockwood MA, Kolykhalov AA, Greninger AL, Moore ML, Painter GR, Lowen AC, Tompkins SM, Fearns R, Natchus MG, Plemper RK (2018) Orally Efficacious Broad-Spectrum Ribonucleoside Analog Inhibitor of Influenza and Respiratory Syncytial Viruses. Antimicrob Agents Chemother 62 (8) DOI 10.1128/AAC.00766-18

16 Taler M, Gil-Ad I, Korob I, Weizman A (2011) The immunomodulatory effect of the antidepressant sertraline in an experimental autoimmune encephalomyelitis mouse model of multiple sclerosis. Neuroimmunomodulation 18 (2): 117-122 DOI 10.1159/000321634

17 Tynan RJ, Weidenhofer J, Hinwood M, Cairns MJ, Day TA, Walker FR (2012) A comparative examination of the anti-inflammatory effects of SSRI and SNRI antidepressants on LPS stimulated microglia. Brain Behav Immun 26 (3): 469-479 DOI 10.1016/j.bbi.2011.12.011

18 Rafiee L, Hajhashemi V, Javanmard SH (2016) Fluvoxamine inhibits some inflammatory genes expression in LPS/stimulated human endothelial cells, U937 macrophages, and carrageenan-induced paw edema in rat. Iran J Basic Med Sci 19 (9): 977-984

19 Naji Esfahani H, Rafiee L, Haghjooy Javanmard S (2019) Evaluation of the Effect of Antidepressant Drug, Fluvoxamine, on Cyclooxygenase-2 Protein Expression in Lipopolysaccharide-stimulated Macrophages. Adv Biomed Res 8: 5 DOI 10.4103/abr.abr_141_18

20 Zuo J, Quinn KK, Kye S, Cooper P, Damoiseaux R, Krogstad P (2012) Fluoxetine is a potent inhibitor of coxsackievirus replication. Antimicrob Agents Chemother 56 (9): 4838-4844 DOI 10.1128/AAC.00983-12

21 Bauer L, Manganaro R, Zonsics B, Strating J, El Kazzi P, Lorenzo Lopez M, Ulferts R, van Hoey C, Mate MJ, Langer T, Coutard B, Brancale A, van Kuppeveld FJM (2019) Fluoxetine Inhibits Enterovirus Replication by Targeting the Viral 2C Protein in a Stereospecific Manner. ACS Infect Dis 5 (9): 1609-1623 DOI 10.1021/acsinfecdis.9b00179

22 Fred SM, Kuivanen S, Ugurlu H, Casarotto PC, Levanov L, Saksela K, Vapalahti O, Castrén E (2021) Antidepressant and antipsychotic drugs reduce viral infection by SARS-CoV-2 and fluoxetine show antiviral activity against the novel variants <em>in vitro</em>. bioRxiv: 2021.2003.2022.436379 DOI 10.1101/2021.03.22.436379

23 Pashaei Y (2021) Drug repurposing of selective serotonin reuptake inhibitors: Could these drugs help fight COVID-19 and save lives? J Clin Neurosci 88: 163-172 DOI 10.1016/j.jocn.2021.03.010

24 Glebov OO (2021) Low-dose fluvoxamine modulates endocytic trafficking of SARS-CoV-2 spike protein: a potential mechanism for anti-COVID-19 protection by antidepressants. bioRxiv: 2021.2006.2015.448391 DOI 10.1101/2021.06.15.448391

25 Son J, Huang S, Zeng Q, Bricker TL, Case JB, Zhou J, Zang R, Liu Z, Chang X, Harastani HH, Chen L, Gomez Castro MF, Zhao Y, Kohio HP, Hou G, Fan B, Niu B, Guo R, Rothlauf PW, Bailey AL, Wang X, Shi P-Y, Martinez ED, Whelan SPJ, Diamond MS, Boon ACM, Li B, Ding S (2021) JIB-04 has broad-spectrum antiviral activity and inhibits SARS-CoV-2 replication and coronavirus pathogenesis. bioRxiv: 2020.2009.2024.312165 DOI 10.1101/2020.09.24.312165

26 Schloer S, Brunotte L, Goretzko J, Mecate-Zambrano A, Korthals N, Gerke V, Ludwig S, Rescher U (2020) Targeting the endolysosomal host-SARS-CoV-2 interface by clinically licensed functional inhibitors of acid sphingomyelinase (FIASMA) including the antidepressant fluoxetine. Emerg Microbes Infect 9 (1): 2245-2255 DOI 10.1080/22221751.2020.1829082

27 Agostini ML, Andres EL, Sims AC, Graham RL, Sheahan TP, Lu X, Smith EC, Case JB, Feng JY, Jordan R, Ray AS, Cihlar T, Siegel D, Mackman RL, Clarke MO, Baric RS, Denison MR (2018) Coronavirus Susceptibility to the Antiviral Remdesivir (GS-5734) Is Mediated by the Viral Polymerase and the Proofreading Exoribonuclease. mBio 9 (2) DOI 10.1128/mBio.00221-18

28 Wang M, Cao R, Zhang L, Yang X, Liu J, Xu M, Shi Z, Hu Z, Zhong W, Xiao G (2020) Remdesivir and chloroquine effectively inhibit the recently emerged novel coronavirus (2019-nCoV) in vitro. Cell Res 30 (3): 269-271 DOI 10.1038/s41422-020-0282-0

29 Gordon CJ, Tchesnokov EP, Feng JY, Porter DP, Götte M (2020) The antiviral compound remdesivir potently inhibits RNA-dependent RNA polymerase from Middle East respiratory syndrome coronavirus. J Biol Chem 295 (15): 4773-4779 DOI 10.1074/jbc.AC120.013056

30 Choy KT, Wong AY, Kaewpreedee P, Sia SF, Chen D, Hui KPY, Chu DKW, Chan MCW, Cheung PP, Huang X, Peiris M, Yen HL (2020) Remdesivir, lopinavir, emetine, and homoharringtonine inhibit SARS-CoV-2 replication in vitro. Antiviral Res 178: 104786 DOI 10.1016/j.antiviral.2020.104786

31 Sheahan TP, Sims AC, Graham RL, Menachery VD, Gralinski LE, Case JB, Leist SR, Pyrc K, Feng JY, Trantcheva I, Bannister R, Park Y, Babusis D, Clarke MO, Mackman RL, Spahn JE, Palmiotti CA, Siegel D, Ray AS, Cihlar T, Jordan R, Denison MR, Baric RS (2017) Broad-spectrum antiviral GS-5734 inhibits both epidemic and zoonotic coronaviruses. Sci Transl Med 9 (396) DOI 10.1126/scitranslmed.aal3653

32 Pruijssers AJ, George AS, Schäfer A, Leist SR, Gralinksi LE, Dinnon KH, Yount BL, Agostini ML, Stevens LJ, Chappell JD, Lu X, Hughes TM, Gully K, Martinez DR, Brown AJ, Graham RL, Perry JK, Du Pont V, Pitts J, Ma B, Babusis D, Murakami E, Feng JY, Bilello JP, Porter DP, Cihlar T, Baric RS, Denison MR, Sheahan TP (2020) Remdesivir potently inhibits SARS-CoV-2 in human lung cells and chimeric SARS-CoV expressing the SARS-CoV-2 RNA polymerase in mice. bioRxiv DOI 10.1101/2020.04.27.064279

33 Brown AJ, Won JJ, Graham RL, Dinnon KH, 3rd, Sims AC, Feng JY, Cihlar T, Denison MR, Baric RS, Sheahan TP (2019) Broad spectrum antiviral remdesivir inhibits human endemic and zoonotic deltacoronaviruses with a highly divergent RNA dependent RNA polymerase. Antiviral Res 169: 104541 DOI 10.1016/j.antiviral.2019.104541

34 Weston S, Coleman CM, Haupt R, Logue J, Matthews K, Frieman MB (2020) Broad anti-coronaviral activity of FDA approved drugs against SARS-CoV-2 <em>in vitro</em> and SARS-CoV <em>in vivo</em>. bioRxiv: 2020.2003.2025.008482 DOI 10.1101/2020.03.25.008482

35 Keyaerts E, Vijgen L, Maes P, Neyts J, Van Ranst M (2004) In vitro inhibition of severe acute respiratory syndrome coronavirus by chloroquine. Biochem Biophys Res Commun 323 (1): 264-268 DOI 10.1016/j.bbrc.2004.08.085

36 Vincent MJ, Bergeron E, Benjannet S, Erickson BR, Rollin PE, Ksiazek TG, Seidah NG, Nichol ST (2005) Chloroquine is a potent inhibitor of SARS coronavirus infection and spread. Virol J 2: 69 DOI 10.1186/1743-422x-2-69

37 Liu J, Cao R, Xu M, Wang X, Zhang H, Hu H, Li Y, Hu Z, Zhong W, Wang M (2020) Hydroxychloroquine, a less toxic derivative of chloroquine, is effective in inhibiting SARS-CoV-2 infection in vitro. Cell Discov 6: 16 DOI 10.1038/s41421-020-0156-0

38 Sheng F, Han M, Huang Z, Zhang L (2016) Interleukin 6 receptor inhibitor tocilizumab suppresses cytokine expression, inflammasome activation and phagocytosis in a cell model of sepsis. Pharmazie 71 (11): 636-639 DOI 10.1691/ph.2016.6713

39 Guo C, Li B, Ma H, Wang X, Cai P, Yu Q, Zhu L, Jin L, Jiang C, Fang J, Liu Q, Zong D, Zhang W, Lu Y, Li K, Gao X, Fu B, Liu L, Ma X, Weng J, Wei H, Jin T, Lin J, Qu K (2020) Single-cell analysis of severe COVID-19 patients reveals a monocyte-driven inflammatory storm attenuated by Tocilizumab. bioRxiv: 2020.2004.2008.029769 DOI 10.1101/2020.04.08.029769

40 Dai JM, Zhang XQ, Zhang JJ, Yang WJ, Yang XM, Bian H, Chen ZN (2022) Blockade of mIL-6R alleviated lipopolysaccharide-induced systemic inflammatory response syndrome by suppressing NF-κB-mediated Ccl2 expression and inflammasome activation. MedComm (2020) 3 (2): e132 DOI 10.1002/mco2.132

41 Iwata-Yoshikawa N, Okamura T, Shimizu Y, Hasegawa H, Takeda M, Nagata N (2019) TMPRSS2 Contributes to Virus Spread and Immunopathology in the Airways of Murine Models after Coronavirus Infection. J Virol 93 (6) DOI 10.1128/JVI.01815-18

42 Sakai K, Ami Y, Tahara M, Kubota T, Anraku M, Abe M, Nakajima N, Sekizuka T, Shirato K, Suzaki Y, Ainai A, Nakatsu Y, Kanou K, Nakamura K, Suzuki T, Komase K, Nobusawa E, Maenaka K, Kuroda M, Hasegawa H, Kawaoka Y, Tashiro M, Takeda M (2014) The host protease TMPRSS2 plays a major role in in vivo replication of emerging H7N9 and seasonal influenza viruses. J Virol 88 (10): 5608-5616 DOI 10.1128/JVI.03677-13

43 Tarnow C, Engels G, Arendt A, Schwalm F, Sediri H, Preuss A, Nelson PS, Garten W, Klenk HD, Gabriel G, Bottcher-Friebertshauser E (2014) TMPRSS2 is a host factor that is essential for pneumotropism and pathogenicity of H7N9 influenza A virus in mice. J Virol 88 (9): 4744-4751 DOI 10.1128/JVI.03799-13

44 Kim TS, Heinlein C, Hackman RC, Nelson PS (2006) Phenotypic analysis of mice lacking the Tmprss2-encoded protease. Mol Cell Biol 26 (3): 965-975 DOI 10.1128/MCB.26.3.965-975.2006

45 Hatesuer B, Bertram S, Mehnert N, Bahgat MM, Nelson PS, Pohlmann S, Schughart K (2013) Tmprss2 is essential for influenza H1N1 virus pathogenesis in mice. PLoS Pathog 9 (12): e1003774 DOI 10.1371/journal.ppat.1003774

46 Zhou Y, Vedantham P, Lu K, Agudelo J, Carrion R, Jr., Nunneley JW, Barnard D, Pohlmann S, McKerrow JH, Renslo AR, Simmons G (2015) Protease inhibitors targeting coronavirus and filovirus entry. Antiviral Res 116: 76-84 DOI 10.1016/j.antiviral.2015.01.011

47 Sheahan TP, Sims AC, Zhou S, Graham RL, Pruijssers AJ, Agostini ML, Leist SR, Schafer A, Dinnon KH, 3rd, Stevens LJ, Chappell JD, Lu X, Hughes TM, George AS, Hill CS, Montgomery SA, Brown AJ, Bluemling GR, Natchus MG, Saindane M, Kolykhalov AA, Painter G, Harcourt J, Tamin A, Thornburg NJ, Swanstrom R, Denison MR, Baric RS (2020) An orally bioavailable broad-spectrum antiviral inhibits SARS-CoV-2 in human airway epithelial cell cultures and multiple coronaviruses in mice. Sci Transl Med 12 (541) DOI 10.1126/scitranslmed.abb5883

48 Wahl A, Gralinski LE, Johnson CE, Yao W, Kovarova M, Dinnon KH, Liu H, Madden VJ, Krzystek HM, De C, White KK, Gully K, Schäfer A, Zaman T, Leist SR, Grant PO, Bluemling GR, Kolykhalov AA, Natchus MG, Askin FB, Painter G, Browne EP, Jones CD, Pickles RJ, Baric RS, Garcia JV (2021) SARS-CoV-2 infection is effectively treated and prevented by EIDD-2801. Nature 591 (7850): 451-457 DOI 10.1038/s41586-021-03312-w

49 Toots M, Yoon JJ, Cox RM, Hart M, Sticher ZM, Makhsous N, Plesker R, Barrena AH, Reddy PG, Mitchell DG, Shean RC, Bluemling GR, Kolykhalov AA, Greninger AL, Natchus MG, Painter GR, Plemper RK (2019) Characterization of orally efficacious influenza drug with high resistance barrier in ferrets and human airway epithelia. Sci Transl Med 11 (515) DOI 10.1126/scitranslmed.aax5866

50 Toots M, Yoon JJ, Hart M, Natchus MG, Painter GR, Plemper RK (2020) Quantitative efficacy paradigms of the influenza clinical drug candidate EIDD-2801 in the ferret model. Transl Res 218: 16-28 DOI 10.1016/j.trsl.2019.12.002

51 Vandyck K, Abdelnabi R, Gupta K, Jochmans D, Jekle A, Deval J, Misner D, Bardiot D, Foo CS, Liu C, Ren S, Beigelman L, Blatt LM, Boland S, Vangeel L, Dejonghe S, Chaltin P, Marchand A, Serebryany V, Stoycheva A, Chanda S, Symons JA, Raboisson P, Neyts J (2021) ALG-097111, a potent and selective SARS-CoV-2 3-chymotrypsin-like cysteine protease inhibitor exhibits in vivo efficacy in a Syrian Hamster model. Biochem Biophys Res Commun 555: 134-139 DOI 10.1016/j.bbrc.2021.03.096

52 Rosen DA, Seki SM, Fernandez-Castaneda A, Beiter RM, Eccles JD, Woodfolk JA, Gaultier A (2019) Modulation of the sigma-1 receptor-IRE1 pathway is beneficial in preclinical models of inflammation and sepsis. Sci Transl Med 11 (478) DOI 10.1126/scitranslmed.aau5266

53 Taler M, Gil-Ad I, Lomnitski L, Korov I, Baharav E, Bar M, Zolokov A, Weizman A (2007) Immunomodulatory effect of selective serotonin reuptake inhibitors (SSRIs) on human T lymphocyte function and gene expression. Eur Neuropsychopharmacol 17 (12): 774-780 DOI 10.1016/j.euroneuro.2007.03.010

54 Gordon DE, Hiatt J, Bouhaddou M, Rezelj VV, Ulferts S, Braberg H, Jureka AS, Obernier K, Guo JZ, Batra J, Kaake RM, Weckstein AR, Owens TW, Gupta M, Pourmal S, Titus EW, Cakir M, Soucheray M, McGregor M, Cakir Z, Jang G, O'Meara MJ, Tummino TA, Zhang Z, Foussard H, Rojc A, Zhou Y, Kuchenov D, Huttenhain R, Xu J, Eckhardt M, Swaney DL, Fabius JM, Ummadi M, Tutuncuoglu B, Rathore U, Modak M, Haas P, Haas KM, Naing ZZC, Pulido EH, Shi Y, Barrio-Hernandez I, Memon D, Petsalaki E, Dunham A, Marrero MC, Burke D, Koh C, Vallet T, Silvas JA, Azumaya CM, Billesbolle C, Brilot AF, Campbell MG, Diallo A, Dickinson MS, Diwanji D, Herrera N, Hoppe N, Kratochvil HT, Liu Y, Merz GE, Moritz M, Nguyen HC, Nowotny C, Puchades C, Rizo AN, Schulze-Gahmen U, Smith AM, Sun M, Young ID, Zhao J, Asarnow D, Biel J, Bowen A, Braxton JR, Chen J, Chio CM, Chio US, Deshpande I, Doan L, Faust B, Flores S, Jin M, Kim K, Lam VL, Li F, Li J, Li YL, Li Y, Liu X, Lo M, Lopez KE, Melo AA, Moss FR, 3rd, Nguyen P, Paulino J, Pawar KI, Peters JK, Pospiech TH, Jr., Safari M, Sangwan S, Schaefer K, Thomas PV, Thwin AC, Trenker R, Tse E, Tsui TKM, Wang F, Whitis N, Yu Z, Zhang K, Zhang Y, Zhou F, Saltzberg D, Consortium QSB, Hodder AJ, Shun-Shion AS, Williams DM, White KM, Rosales R, Kehrer T, Miorin L, Moreno E, Patel AH, Rihn S, Khalid MM, Vallejo-Gracia A, Fozouni P, Simoneau CR, Roth TL, Wu D, Karim MA, Ghoussaini M, Dunham I, Berardi F, Weigang S, Chazal M, Park J, Logue J, McGrath M, Weston S, Haupt R, Hastie CJ, Elliott M, Brown F, Burness KA, Reid E, Dorward M, Johnson C, Wilkinson SG, Geyer A, Giesel DM, Baillie C, Raggett S, Leech H, Toth R, Goodman N, Keough KC, Lind AL, Zoonomia C, Klesh RJ, Hemphill KR, Carlson-Stevermer J, Oki J, Holden K, Maures T, Pollard KS, Sali A, Agard DA, Cheng Y, Fraser JS, Frost A, Jura N, Kortemme T, Manglik A, Southworth DR, Stroud RM, Alessi DR, Davies P, Frieman MB, Ideker T, Abate C, Jouvenet N, Kochs G, Shoichet B, Ott M, Palmarini M, Shokat KM, Garcia-Sastre A, Rassen JA, Grosse R, Rosenberg OS, Verba KA, Basler CF, Vignuzzi M, Peden AA, Beltrao P, Krogan NJ (2020) Comparative host-coronavirus protein interaction networks reveal pan-viral disease mechanisms. Science 370 (6521) DOI 10.1126/science.abe9403

55 de Wit E, Feldmann F, Cronin J, Jordan R, Okumura A, Thomas T, Scott D, Cihlar T, Feldmann H (2020) Prophylactic and therapeutic remdesivir (GS-5734) treatment in the rhesus macaque model of MERS-CoV infection. Proc Natl Acad Sci U S A 117 (12): 6771-6776 DOI 10.1073/pnas.1922083117

56 Sheahan TP, Sims AC, Leist SR, Schäfer A, Won J, Brown AJ, Montgomery SA, Hogg A, Babusis D, Clarke MO, Spahn JE, Bauer L, Sellers S, Porter D, Feng JY, Cihlar T, Jordan R, Denison MR, Baric RS (2020) Comparative therapeutic efficacy of remdesivir and combination lopinavir, ritonavir, and interferon beta against MERS-CoV. Nat Commun 11 (1): 222 DOI 10.1038/s41467-019-13940-6

57 Williamson BN, Feldmann F, Schwarz B, Meade-White K, Porter DP, Schulz J, van Doremalen N, Leighton I, Kwe Yinda C, Pérez-Pérez L, Okumura A, Lovaglio J, Hanley PW, Saturday G, Bosio CM, Anzick S, Barbian K, Cihlar T, Martens C, Scott DP, Munster VJ, de Wit E (2020) Clinical benefit of remdesivir in rhesus macaques infected with SARS-CoV-2. bioRxiv DOI 10.1101/2020.04.15.043166

58 Keyaerts E, Li S, Vijgen L, Rysman E, Verbeeck J, Van Ranst M, Maes P (2009) Antiviral activity of chloroquine against human coronavirus OC43 infection in newborn mice. Antimicrob Agents Chemother 53 (8): 3416-3421 DOI 10.1128/aac.01509-08

59 Radigan KA, Nicholson TT, Welch LC, Chi M, Amarelle L, Angulo M, Shigemura M, Shigemura A, Runyan CE, Morales-Nebreda L, Perlman H, Ceco E, Lecuona E, Dada LA, Misharin AV, Mutlu GM, Sznajder JI, Budinger GRS (2019) Influenza A Virus Infection Induces Muscle Wasting via IL-6 Regulation of the E3 Ubiquitin Ligase Atrogin-1. J Immunol 202 (2): 484-493 DOI 10.4049/jimmunol.1701433

60 Callaway E (2020) Labs rush to study coronavirus in transgenic animals - some are in short supply. Nature 579 (7798): 183 DOI 10.1038/d41586-020-00698-x

61 Woolsey C, Borisevich V, Prasad AN, Agans KN, Deer DJ, Dobias NS, Heymann JC, Foster SL, Levine CB, Medina L, Melody K, Geisbert JB, Fenton KA, Geisbert TW, Cross RW (2020) Establishment of an African green monkey model for COVID-19. bioRxiv: 2020.2005.2017.100289 DOI 10.1101/2020.05.17.100289

62 Weinhold B, Rüther U (1997) Interleukin-6-dependent and -independent regulation of the human C-reactive protein gene. Biochem J 327 ( Pt 2) (Pt 2): 425-429 DOI 10.1042/bj3270425

63 Grein J, Ohmagari N, Shin D, Diaz G, Asperges E, Castagna A, Feldt T, Green G, Green ML, Lescure FX, Nicastri E, Oda R, Yo K, Quiros-Roldan E, Studemeister A, Redinski J, Ahmed S, Bernett J, Chelliah D, Chen D, Chihara S, Cohen SH, Cunningham J, D'Arminio Monforte A, Ismail S, Kato H, Lapadula G, L'Her E, Maeno T, Majumder S, Massari M, Mora-Rillo M, Mutoh Y, Nguyen D, Verweij E, Zoufaly A, Osinusi AO, DeZure A, Zhao Y, Zhong L, Chokkalingam A, Elboudwarej E, Telep L, Timbs L, Henne I, Sellers S, Cao H, Tan SK, Winterbourne L, Desai P, Mera R, Gaggar A, Myers RP, Brainard DM, Childs R, Flanigan T (2020) Compassionate Use of Remdesivir for Patients with Severe Covid-19. N Engl J Med 382 (24): 2327-2336 DOI 10.1056/NEJMoa2007016

64 David A, Parkinson N, Peacock TP, Pairo-Castineira E, Khanna T, Cobat A, Tenesa A, Sancho-Shimizu V, Casanova J-L, Abel L, Barclay WS, Baillie JK, Sternberg MJ (2021) A common <em>TMPRSS2</em> variant protects against severe COVID-19. medRxiv: 2021.2003.2004.21252931 DOI 10.1101/2021.03.04.21252931

65 Montopoli M, Zumerle S, Vettor R, Rugge M, Zorzi M, Catapano CV, Carbone GM, Cavalli A, Pagano F, Ragazzi E, Prayer-Galetti T, Alimonti A (2020) Androgen-deprivation therapies for prostate cancer and risk of infection by SARS-CoV-2: a population-based study (N = 4532). Ann Oncol 31 (8): 1040-1045 DOI 10.1016/j.annonc.2020.04.479

66 Clinckemalie L, Spans L, Dubois V, Laurent M, Helsen C, Joniau S, Claessens F (2013) Androgen regulation of the TMPRSS2 gene and the effect of a SNP in an androgen response element. Mol Endocrinol 27 (12): 2028-2040 DOI 10.1210/me.2013-1098

67 Hou Y, Zhao J, Martin W, Kallianpur A, Chung MK, Jehi L, Sharifi N, Erzurum S, Eng C, Cheng F (2020) New insights into genetic susceptibility of COVID-19: an ACE2 and TMPRSS2 polymorphism analysis. BMC Med 18 (1): 216 DOI 10.1186/s12916-020-01673-z

68 Lehrer S, Rheinstein PH (2021) Homozygosity for rs17775810 Minor Allele Associated With Reduced Mortality of COVID-19 in the UK Biobank Cohort. In Vivo 35 (2): 965-968 DOI 10.21873/invivo.12338

69 Bovijn J, Lindgren CM, Holmes MV (2020) Genetic inhibition of interleukin-6 receptor signaling and Covid-19. medRxiv: 2020.2007.2017.20155242 DOI 10.1101/2020.07.17.20155242

70 Hiatt WR (2006) Observational studies of drug safety--aprotinin and the absence of transparency. N Engl J Med 355 (21): 2171-2173 DOI 10.1056/NEJMp068252

71 Painter WP, Holman W, Bush JA, Almazedi F, Malik H, Eraut N, Morin MJ, Szewczyk LJ, Painter GR (2021) Human Safety, Tolerability, and Pharmacokinetics of Molnupiravir, a Novel Broad-Spectrum Oral Antiviral Agent with Activity Against SARS-CoV-2. Antimicrob Agents Chemother DOI 10.1128/AAC.02428-20

72 Beigel JH, Tomashek KM, Dodd LE, Mehta AK, Zingman BS, Kalil AC, Hohmann E, Chu HY, Luetkemeyer A, Kline S, Lopez de Castilla D, Finberg RW, Dierberg K, Tapson V, Hsieh L, Patterson TF, Paredes R, Sweeney DA, Short WR, Touloumi G, Lye DC, Ohmagari N, Oh MD, Ruiz-Palacios GM, Benfield T, Fatkenheuer G, Kortepeter MG, Atmar RL, Creech CB, Lundgren J, Babiker AG, Pett S, Neaton JD, Burgess TH, Bonnett T, Green M, Makowski M, Osinusi A, Nayak S, Lane HC, Members A-SG (2020) Remdesivir for the Treatment of Covid-19 - Final Report. N Engl J Med 383 (19): 1813-1826 DOI 10.1056/NEJMoa2007764

73 Wang Y, Zhang D, Du G, Du R, Zhao J, Jin Y, Fu S, Gao L, Cheng Z, Lu Q, Hu Y, Luo G, Wang K, Lu Y, Li H, Wang S, Ruan S, Yang C, Mei C, Wang Y, Ding D, Wu F, Tang X, Ye X, Ye Y, Liu B, Yang J, Yin W, Wang A, Fan G, Zhou F, Liu Z, Gu X, Xu J, Shang L, Zhang Y, Cao L, Guo T, Wan Y, Qin H, Jiang Y, Jaki T, Hayden FG, Horby PW, Cao B, Wang C (2020) Remdesivir in adults with severe COVID-19: a randomised, double-blind, placebo-controlled, multicentre trial. Lancet 395 (10236): 1569-1578 DOI 10.1016/S0140-6736(20)31022-9

74 Consortium WHOST, Pan H, Peto R, Henao-Restrepo AM, Preziosi MP, Sathiyamoorthy V, Abdool Karim Q, Alejandria MM, Hernandez Garcia C, Kieny MP, Malekzadeh R, Murthy S, Reddy KS, Roses Periago M, Abi Hanna P, Ader F, Al-Bader AM, Alhasawi A, Allum E, Alotaibi A, Alvarez-Moreno CA, Appadoo S, Asiri A, Aukrust P, Barratt-Due A, Bellani S, Branca M, Cappel-Porter HBC, Cerrato N, Chow TS, Como N, Eustace J, Garcia PJ, Godbole S, Gotuzzo E, Griskevicius L, Hamra R, Hassan M, Hassany M, Hutton D, Irmansyah I, Jancoriene L, Kirwan J, Kumar S, Lennon P, Lopardo G, Lydon P, Magrini N, Maguire T, Manevska S, Manuel O, McGinty S, Medina MT, Mesa Rubio ML, Miranda-Montoya MC, Nel J, Nunes EP, Perola M, Portoles A, Rasmin MR, Raza A, Rees H, Reges PPS, Rogers CA, Salami K, Salvadori MI, Sinani N, Sterne JAC, Stevanovikj M, Tacconelli E, Tikkinen KAO, Trelle S, Zaid H, Rottingen JA, Swaminathan S (2021) Repurposed Antiviral Drugs for Covid-19 - Interim WHO Solidarity Trial Results. N Engl J Med 384 (6): 497-511 DOI 10.1056/NEJMoa2023184

75 Dabbous HM, Abd-Elsalam S, El-Sayed MH, Sherief AF, Ebeid FFS, El Ghafar MSA, Soliman S, Elbahnasawy M, Badawi R, Tageldin MA (2021) Efficacy of favipiravir in COVID-19 treatment: a multi-center randomized study. Arch Virol 166 (3): 949-954 DOI 10.1007/s00705-021-04956-9

76 Ghasemnejad-Berenji M, Pashapour S (2021) Favipiravir and COVID-19: A Simplified Summary. Drug Res (Stuttg) 71 (3): 166-170 DOI 10.1055/a-1296-7935

77 Udwadia ZF, Singh P, Barkate H, Patil S, Rangwala S, Pendse A, Kadam J, Wu W, Caracta CF, Tandon M (2021) Efficacy and safety of favipiravir, an oral RNA-dependent RNA polymerase inhibitor, in mild-to-moderate COVID-19: A randomized, comparative, open-label, multicenter, phase 3 clinical trial. Int J Infect Dis 103: 62-71 DOI 10.1016/j.ijid.2020.11.142

78 Elalfy H, Besheer T, El-Mesery A, El-Gilany AH, Soliman MA, Alhawarey A, Alegezy M, Elhadidy T, Hewidy AA, Zaghloul H, Neamatallah MAM, Raafat D, El-Emshaty WM, Abo El Kheir NY, El-Bendary M (2021) Effect of a combination of nitazoxanide, ribavirin, and ivermectin plus zinc supplement (MANS.NRIZ study) on the clearance of mild COVID-19. J Med Virol 93 (5): 3176-3183 DOI 10.1002/jmv.26880

79 Hoertel N, Sanchez-Rico M, Vernet R, Beeker N, Jannot AS, Neuraz A, Salamanca E, Paris N, Daniel C, Gramfort A, Lemaitre G, Bernaux M, Bellamine A, Lemogne C, Airagnes G, Burgun A, Limosin F, Collaboration A-HUIC-R, Initiative A-HCC (2021) Association between antidepressant use and reduced risk of intubation or death in hospitalized patients with COVID-19: results from an observational study. Mol Psychiatry DOI 10.1038/s41380-021-01021-4

80 Hoertel N, Sanchez-Rico M, Gulbins E, Kornhuber J, Carpinteiro A, Lenze EJ, Reiersen AM, Abellan M, De La Muela P, Vernet R, Blanco C, Cougoule C, Beeker N, Neuraz A, Gorwood P, Alvarado JM, Meneton P, Limosin F, Ap-Hp / Universite de Paris / Inserm Covid-19 research collaboration APHPCCDRIEdDdSA (2021) Association between FIASMAs and Reduced Risk of Intubation or Death in Individuals Hospitalized for Severe COVID-19: an observational multicenter study. Clin Pharmacol Ther DOI 10.1002/cpt.2317

81 Cai Q, Yang M, Liu D, Chen J, Shu D, Xia J, Liao X, Gu Y, Cai Q, Yang Y, Shen C, Li X, Peng L, Huang D, Zhang J, Zhang S, Wang F, Liu J, Chen L, Chen S, Wang Z, Zhang Z, Cao R, Zhong W, Liu Y, Liu L (2020) Experimental Treatment with Favipiravir for COVID-19: An Open-Label Control Study. Engineering (Beijing) 6 (10): 1192-1198 DOI 10.1016/j.eng.2020.03.007

82 Khalili JS, Zhu H, Mak NSA, Yan Y, Zhu Y (2020) Novel coronavirus treatment with ribavirin: Groundwork for an evaluation concerning COVID-19. J Med Virol 92 (7): 740-746 DOI 10.1002/jmv.25798

83 Gremese E, Cingolani A, Bosello SL, Alivernini S, Tolusso B, Perniola S, Landi F, Pompili M, Murri R, Santoliquido A, Garcovich M, Sali M, Pascale GD, Gabrielli M, Biscetti F, Montalto M, Tosoni A, Gambassi G, Rapaccini GL, Iaconelli A, Verme LZD, Petricca L, Fedele AL, Lizzio MM, Tamburrini E, Natalello G, Gigante L, Bruno D, Verardi L, Taddei E, Calabrese A, Lombardi F, Bernabei R, Cauda R, Franceschi F, Landolfi R, Richeldi L, Sanguinetti M, Fantoni M, Antonelli M, Gasbarrini A, COVID obotGA (2020) Sarilumab use in severe SARS-CoV-2 pneumonia. medRxiv: 2020.2005.2014.20094144 DOI 10.1101/2020.05.14.20094144

84 Hofmann-Winkler H, Moerer O, Alt-Epping S, Brauer A, Buttner B, Muller M, Fricke T, Grundmann J, Harnisch LO, Heise D, Kernchen A, Pressler M, Stephani C, Tampe B, Kaul A, Gartner S, Kramer S, Pohlmann S, Winkler MS (2020) Camostat Mesylate May Reduce Severity of Coronavirus Disease 2019 Sepsis: A First Observation. Crit Care Explor 2 (11): e0284 DOI 10.1097/CCE.0000000000000284

85 Gunst JD, Staerke NB, Pahus MH, Kristensen LH, Bodilsen J, Lohse N, Dalgaard LS, Bronnum D, Frobert O, Honge B, Johansen IS, Monrad I, Erikstrup C, Rosendal R, Vilstrup E, Mariager T, Bove DG, Offersen R, Shakar S, Cajander S, Jorgensen NP, Sritharan SS, Breining P, Jespersen S, Mortensen KL, Jensen ML, Kolte L, Frattari GS, Larsen CS, Storgaard M, Nielsen LP, Tolstrup M, Saedder EA, Ostergaard LJ, Ngo HTT, Jensen MH, Hojen JF, Kjolby M, Sogaard OS (2021) Efficacy of the TMPRSS2 inhibitor camostat mesilate in patients hospitalized with Covid-19-a double-blind randomized controlled trial. EClinicalMedicine: 100849 DOI 10.1016/j.eclinm.2021.100849

86 Khoo SH, FitzGerald R, Fletcher T, Ewings S, Jaki T, Lyon R, Downs N, Walker L, Tansley-Hancock O, Greenhalf W, Woods C, Reynolds H, Marwood E, Mozgunov P, Adams E, Bullock K, Holman W, Bula MD, Gibney JL, Saunders G, Corkhill A, Hale C, Thorne K, Chiong J, Condie S, Pertinez H, Painter W, Wrixon E, Johnson L, Yeats S, Mallard K, Radford M, Fines K, Shaw V, Owen A, Lalloo DG, Jacobs M, Griffiths G (2021) Optimal dose and safety of molnupiravir in patients with early SARS-CoV-2: a phase 1, dose-escalating, randomised controlled study. medRxiv: 2021.2005.2003.21256309 DOI 10.1101/2021.05.03.21256309

87 Fischer W, Eron JJ, Holman W, Cohen MS, Fang L, Szewczyk LJ, Sheahan TP, Baric R, Mollan KR, Wolfe CR, Duke ER, Azizad MM, Borroto-Esoda K, Wohl DA, Loftis AJ, Alabanza P, Lipansky F, Painter WP (2021) Molnupiravir, an Oral Antiviral Treatment for COVID-19. medRxiv: 2021.2006.2017.21258639 DOI 10.1101/2021.06.17.21258639

88 Seftel D, Boulware DR (2021) Prospective Cohort of Fluvoxamine for Early Treatment of Coronavirus Disease 19. Open Forum Infect Dis 8 (2): ofab050 DOI 10.1093/ofid/ofab050

89 Lenze EJ, Mattar C, Zorumski CF, Stevens A, Schweiger J, Nicol GE, Miller JP, Yang L, Yingling M, Avidan MS, Reiersen AM (2020) Fluvoxamine vs Placebo and Clinical Deterioration in Outpatients With Symptomatic COVID-19: A Randomized Clinical Trial. JAMA 324 (22): 2292-2300 DOI 10.1001/jama.2020.22760

90 Higgs ES, Gayedyu-Dennis D, Fisher W, Nason M, Reilly C, Beavogui AH, Aboulhab J, Nordwall J, Lobbo P, Wachekwa I, Cao H, Cihlar T, Hensley L, Lane HC (2021) PREVAIL IV: A Randomized, Double-Blind, Two-Phase, Phase 2 Trial of Remdesivir versus Placebo for Reduction of Ebola Virus RNA in the Semen of Male Survivors. Clin Infect Dis DOI 10.1093/cid/ciab215

91 Mulangu S, Dodd LE, Davey RT, Jr., Tshiani Mbaya O, Proschan M, Mukadi D, Lusakibanza Manzo M, Nzolo D, Tshomba Oloma A, Ibanda A, Ali R, Coulibaly S, Levine AC, Grais R, Diaz J, Lane HC, Muyembe-Tamfum JJ, Sivahera B, Camara M, Kojan R, Walker R, Dighero-Kemp B, Cao H, Mukumbayi P, Mbala-Kingebeni P, Ahuka S, Albert S, Bonnett T, Crozier I, Duvenhage M, Proffitt C, Teitelbaum M, Moench T, Aboulhab J, Barrett K, Cahill K, Cone K, Eckes R, Hensley L, Herpin B, Higgs E, Ledgerwood J, Pierson J, Smolskis M, Sow Y, Tierney J, Sivapalasingam S, Holman W, Gettinger N, Vallée D, Nordwall J (2019) A Randomized, Controlled Trial of Ebola Virus Disease Therapeutics. N Engl J Med 381 (24): 2293-2303 DOI 10.1056/NEJMoa1910993

92 Gautret P, Lagier J-C, Parola P, Hoang VT, Meddeb L, Mailhe M, Doudier B, Courjon J, Giordanengo V, Vieira VE, Dupont HT, Honoré S, Colson P, Chabrière E, Scola BL, Rolain J-M, Brouqui P, Raoult D (2020) Hydroxychloroquine and azithromycin as a treatment of COVID-19: results of an open-label non-randomized clinical trial. medRxiv: 2020.2003.2016.20037135 DOI 10.1101/2020.03.16.20037135

93 Gao J, Tian Z, Yang X (2020) Breakthrough: Chloroquine phosphate has shown apparent efficacy in treatment of COVID-19 associated pneumonia in clinical studies. Biosci Trends 14 (1): 72-73 DOI 10.5582/bst.2020.01047

94 Singh S, Moore TJ (2020) Efficacy and Safety of Hydroxychloroquine and Chloroquine for COVID-19: A Systematic Review. medRxiv: 2020.2005.2019.20106906 DOI 10.1101/2020.05.19.20106906

95 Takla M, Jeevaratnam K (2020) Chloroquine, hydroxychloroquine, and COVID-19: systematic review and narrative synthesis of efficacy and safety: Systematic review of (hydroxy)chloroquine efficacy and safety. medRxiv: 2020.2005.2028.20115741 DOI 10.1101/2020.05.28.20115741

96 Kapoor KM, Kapoor A (2020) Role of Chloroquine and Hydroxychloroquine in the Treatment of COVID-19 Infection- A Systematic Literature Review. medRxiv: 2020.2003.2024.20042366 DOI 10.1101/2020.03.24.20042366

97 Mallat J, Hamed F, Balkis M, Mohamed MA, Mooty M, Malik A, Nusair A, Bonilla M-F (2020) Hydroxychloroquine is associated with slower viral clearance in clinical COVID-19 patients with mild to moderate disease: A retrospective study. medRxiv: 2020.2004.2027.20082180 DOI 10.1101/2020.04.27.20082180

98 Touret F, de Lamballerie X (2020) Of chloroquine and COVID-19. Antiviral Res 177: 104762 DOI 10.1016/j.antiviral.2020.104762

99 Rawaf S, Al-Saffar MN, Quezada-Yamamoto H, Alshaikh M, Pelly M, Rawaf D, Dubois E, Majeed A (2020) Chloroquine and hydroxychloroquine effectiveness in human subjects during coronavirus: a systematic review. medRxiv: 2020.2005.2007.20094326 DOI 10.1101/2020.05.07.20094326

100 Wang Y, Liang S, Qiu T, Han R, Dabbous M, Nowotarska A, Toumi M (2020) Rapid systematic review on clinical evidence of chloroquine and hydroxychloroquine in COVID-19: critical assessment and recommendation for future clinical trials. medRxiv: 2020.2006.2001.20118901 DOI 10.1101/2020.06.01.20118901

101 Huang M, Li M, Xiao F, Liang J, Pang P, Tang T, Liu S, Chen B, Shu J, You Y, Li Y, Tang M, Zhou J, Jiang G, Xiang J, Hong W, He S, Wang Z, Feng J, Lin C, Ye Y, Wu Z, Li Y, Zhong B, Sun R, Hong Z, Liu J, Chen H, Wang X, Li Z, Pei D, Tian L, Xia J, Jiang S, Zhong N, Shan H (2020) Preliminary evidence from a multicenter prospective observational study of the safety and efficacy of chloroquine for the treatment of COVID-19. medRxiv: 2020.2004.2026.20081059 DOI 10.1101/2020.04.26.20081059

102 Wang J, Yu L, Li K (2020) Benefits and Risks of Chloroquine and Hydroxychloroquine in The Treatment of Viral Diseases: A Meta-Analysis of Placebo Randomized Controlled Trials. medRxiv: 2020.2004.2013.20064295 DOI 10.1101/2020.04.13.20064295

103 Borba MGS, Val FFA, Sampaio VS, Alexandre MAA, Melo GC, Brito M, Mourão MPG, Brito-Sousa JD, Baía-da-Silva D, Guerra MVF, Hajjar LA, Pinto RC, Balieiro AAS, Pacheco AGF, Santos JDO, Jr., Naveca FG, Xavier MS, Siqueira AM, Schwarzbold A, Croda J, Nogueira ML, Romero GAS, Bassat Q, Fontes CJ, Albuquerque BC, Daniel-Ribeiro CT, Monteiro WM, Lacerda MVG (2020) Effect of High vs Low Doses of Chloroquine Diphosphate as Adjunctive Therapy for Patients Hospitalized With Severe Acute Respiratory Syndrome Coronavirus 2 (SARS-CoV-2) Infection: A Randomized Clinical Trial. JAMA Netw Open 3 (4): e208857 DOI 10.1001/jamanetworkopen.2020.8857

104 Xu X, Han M, Li T, Sun W, Wang D, Fu B, Zhou Y, Zheng X, Yang Y, Li X, Zhang X, Pan A, Wei H (2020) Effective treatment of severe COVID-19 patients with tocilizumab. Proc Natl Acad Sci U S A 117 (20): 10970-10975 DOI 10.1073/pnas.2005615117

105 Wang L, Peng X, Wang ZH, Cai J, Zhou FC (2020) Tocilizumab in the treatment of a critical COVID-19 patient: a case report. Eur Rev Med Pharmacol Sci 24 (10): 5783-5787 DOI 10.26355/eurrev_202005_21372

106 Capra R, De Rossi N, Mattioli F, Romanelli G, Scarpazza C, Sormani MP, Cossi S (2020) Impact of low dose tocilizumab on mortality rate in patients with COVID-19 related pneumonia. Eur J Intern Med 76: 31-35 DOI 10.1016/j.ejim.2020.05.009

107 Knorr JP, Colomy V, Mauriello CM, Ha S (2020) Tocilizumab in patients with severe COVID-19: A single-center observational analysis. J Med Virol 92 (11): 2813-2820 DOI 10.1002/jmv.26191

108 Lucio Liberato N, De Monte A, Caravella G (2020) Tocilizumab in severe COVID-19. Arch Med Sci 16 (6): 1457-1458 DOI 10.5114/aoms.2020.97411

109 Carvalho V, Turon R, Gonçalves B, Ceotto VF, Kurtz P, Righy C (2020) Effects of Tocilizumab in Critically Ill Patients With COVID-19: A Quasi-Experimental Study. medRxiv: 2020.2007.2013.20149328 DOI 10.1101/2020.07.13.20149328

110 Strohbehn GW, Heiss BL, Rouhani SJ, Trujillo JA, Yu J, Kacew AJ, Higgs EF, Bloodworth JC, Cabanov A, Wright RC, Koziol AK, Weiss A, Danahey K, Karrison TG, Edens CC, Ventura IB, Pettit NN, Patel BK, Pisano J, Strek ME, Gajewski TF, Ratain MJ, Reid PD (2020) COVIDOSE: Low-dose tocilizumab in the treatment of Covid-19. medRxiv: 2020.2007.2020.20157503 DOI 10.1101/2020.07.20.20157503

111 Somers EC, Eschenauer GA, Troost JP, Golob JL, Gandhi TN, Wang L, Zhou N, Petty LA, Baang JH, Dillman NO, Frame D, Gregg KS, Kaul DR, Nagel J, Patel TS, Zhou S, Lauring AS, Hanauer DA, Martin E, Sharma P, Fung CM, Pogue JM (2020) Tocilizumab for treatment of mechanically ventilated patients with COVID-19. medRxiv: 2020.2005.2029.20117358 DOI 10.1101/2020.05.29.20117358

112 Rossi B, Nguyen LS, Zimmermann P, Boucenna F, Dubret L, Baucher L, Guillot H, Bouldouyre M-A, Allenbach Y, Salem J-E, Barsoum P, Oufella A, Gros H (2020) Effect of tocilizumab in hospitalized patients with severe pneumonia COVID-19: a cohort study. medRxiv: 2020.2006.2006.20122341 DOI 10.1101/2020.06.06.20122341

113 Perrone F, Piccirillo MC, Ascierto PA, Salvarani C, Parrella R, Marata AM, Popoli P, Ferraris L, Marrocco-Trischitta MM, Ripamonti D, Binda F, Bonfanti P, Squillace N, Castelli F, Muiesan ML, Lichtner M, Calzetti C, Salerno ND, Atripaldi L, Cascella M, Costantini M, Dolci G, Facciolongo NC, Fraganza F, Massari M, Montesarchio V, Mussini C, Negri EA, Botti G, Cardone C, Gargiulo P, Gravina A, Schettino C, Arenare L, Chiodini P, Gallo C, on behalf of the TOCIVID-19 investigators I (2020) Tocilizumab for patients with COVID-19 pneumonia. The TOCIVID-19 prospective phase 2 trial. medRxiv: 2020.2006.2001.20119149 DOI 10.1101/2020.06.01.20119149

114 Mikulska M, Nicolini LA, Signori A, Di Biagio A, Sepulcri C, Russo C, Dettori S, Berruti a, Sormani MP, Giacobbe DR, Vena A, De Maria A, Dentone C, Taramasso L, Mirabella M, Magnasco L, Mora S, Delfino E, Toscanini F, Balletto E, Alessandrini AI, Baldi F, Briano F, Camera M, Dodi F, Ferrazin A, Labate L, Mazzarello G, Pincino R, Portunato F, Tutino S, Barisione E, Bruzzone B, Orsi A, Schenone E, Rosseti N, Sasso E, Rin GD, Pelosi P, Beltramini S, Giacomini M, Icardi G, Gratarola A, Bassetti M (2020) Tocilizumab and steroid treatment in patients with COVID-19 pneumonia. medRxiv: 2020.2006.2022.20133413 DOI 10.1101/2020.06.22.20133413

115 Martínez-Sanz J, Muriel A, Ron R, Herrera S, Pérez-Molina JA, Moreno S, Serrano-Villar S (2020) Effects of Tocilizumab on Mortality in Hospitalized Patients with COVID-19: A Multicenter Cohort Study. medRxiv: 2020.2006.2008.20125245 DOI 10.1101/2020.06.08.20125245

116 Ip A, Berry DA, Hansen E, Goy AH, Pecora AL, Sinclaire BA, Bednarz U, Marafelias M, Berry SM, Berry NS, Mathura S, Sawczuk IS, Biran N, Go RC, Sperber S, Piwoz JA, Balani B, Cicogna C, Sebti R, Zuckerman J, Rose KM, Tank L, Jacobs LG, Korcak J, Timmapuri SL, Underwood JP, Sugalski G, Barsky C, Varga DW, Asif A, Landolfi JC, Goldberg SL (2020) Hydroxychloroquine and Tocilizumab Therapy in COVID-19 Patients – An Observational Study. medRxiv: 2020.2005.2021.20109207 DOI 10.1101/2020.05.21.20109207

117 Nasir N, Mahmood SF, Habib K, Khanum I, Jamil B (2020) Treatment of ARDS and hyperinflammation in COVID-19 with IL-6 antagonist Tocilizumab: a tertiary care experience from Pakistan. medRxiv: 2020.2006.2023.20134072 DOI 10.1101/2020.06.23.20134072

118 Rimland CA, Morgan CE, Bell GJ, Kim MK, Hedrick T, Marx A, Bramson B, Swygard H, Napravnik S, Schmitz JL, Carson SS, Fischer WA, Eron JJ, Gay CL, Parr JB (2020) Clinical characteristics and early outcomes in patients with COVID-19 treated with tocilizumab at a United States academic center. medRxiv: 2020.2005.2013.20100404 DOI 10.1101/2020.05.13.20100404

119 Fomina DS, Lysenko MyA, Beloglazova IP, Mutovina ZY, Poteshkina NG, Samsonova IV, Kruglova TyS, Chernov AA, Karaulov AV, Lederman MM (2020) Temporal clinical and laboratory response to interleukin-6 receptor blockade with Tocilizumab in 89 hospitalized patients with COVID-19 pneumonia. medRxiv: 2020.2006.2012.20122374 DOI 10.1101/2020.06.12.20122374

120 Wadud N, Ahmed N, Shergil M, Khan M, Krishna M, Gilani A, Zarif SE, Galaydick J, Linga K, Koor S, Galea J, Stuczynski L, Osundele MB (2020) Improved survival outcome in SARs-CoV-2 (COVID-19) Acute Respiratory Distress Syndrome patients with Tocilizumab administration. medRxiv: 2020.2005.2013.20100081 DOI 10.1101/2020.05.13.20100081

121 Petrak RM, Skorodin NC, Van Hise NW, Fliegelman RM, Pinsky J, Didwania V, Anderson M, Diaz M, Shah K, Chundi VV, Hines DW, Harting BP, Sidwha K, Yu B, Brune P, Owaisi A, Beezhold D, Kent J, Vais D, Han A, Gowda N, Sahgal N, Silverman J, Stake J, Nepomuceno J, Heddurshetti R (2020) Tocilizumab as a Therapeutic Agent for Critically Ill Patients Infected with SARS-CoV-2. medRxiv: 2020.2006.2005.20122622 DOI 10.1101/2020.06.05.20122622

122 Hernández-Mora MG, Úbeda AC, Pérez LP, Álvarez FV, Álvarez BÁ, Rodríguez Nieto MJ, Acosta IC, Fernández Ormaechea I, Mohammed Al-Hayani AW, Carballosa P, Martínez SC, Ezzine F, González MC, Naya A, de las Heras ML, Rodríguez Guzmán MJ, Guijarro AC, Lavado AB, Valcayo AM, García MM, Martínez JB, Roblas RF, Piris Pinilla MÁ, Alen JF, Pernaute OS, Bueno FR, Frades SH, Barba Romero GP, COVID-FJD-TEAM t (2020) Compassionate Use of Tocilizumab in Severe SARS-CoV2 Pneumonia. When late administration is too late. medRxiv: 2020.2006.2013.20130088 DOI 10.1101/2020.06.13.20130088

123 Ramaswamy M, Mannam P, Comer R, Sinclair E, McQuaid DB, Schmidt ML (2020) Off-Label Real World Experience Using Tocilizumab for Patients Hospitalized with COVID-19 Disease in a Regional Community Health System: A Case-Control Study. medRxiv: 2020.2005.2014.20099234 DOI 10.1101/2020.05.14.20099234

124 Quartuccio L, Sonaglia A, McGonagle D, Fabris M, Peghin M, Pecori D, Monte AD, Bove T, Curcio F, Bassi F, De Vita S, Tascini C (2020) Profiling COVID-19 pneumonia progressing into the cytokine storm syndrome: results from a single Italian Centre study on tocilizumab versus standard of care. medRxiv: 2020.2005.2001.20078360 DOI 10.1101/2020.05.01.20078360

125 Moreno-García E, Rico V, Albiach L, Agüero D, Ambrosioni J, Bodro M, Cardozo C, Chumbita M, De la Mora L, García-Pouton N, Garcia-Vidal C, González-Cordón A, Hernández-Meneses M, Inciarte A, Laguno M, Leal L, Linares L, Macaya I, Meira F, Mensa J, Moreno A, Morata L, Puerta-Alcalde P, Rojas J, Solá M, Torres B, Torres M, Tomé A, Castro P, Fernández S, Nicolás JM, Almuedo-Riera A, Muñoz J, Fernandez-Pittol MJ, Marcos MA, Soy D, Martínez JA, García F, Soriano A (2020) Tocilizumab is associated with reduced risk of ICU admission and mortality in patients with SARS-CoV-2 infection. medRxiv: 2020.2006.2005.20113738 DOI 10.1101/2020.06.05.20113738

126 Sánchez-Montalvá A, Sellarés-Nadal J, Espinosa-Pereiro J, Fernández-Hidalgo N, Pérez-Hoyos S, Salvador F, Durà X, Miarons M, Antón A, Eremiev-Eremiev S, Sempere-González A, Monforte-Pallarés A, Bosch-Nicolau P, Augustin S, Sampol J, Guillén-del-Castillo A, Almirante B (2020) Early outcomes of tocilizumab in adults hospitalized with severe COVID-19 - The Vall d’Hebron COVID-19 prospective cohort study. medRxiv: 2020.2005.2007.20094599 DOI 10.1101/2020.05.07.20094599

127 Roumier M, Paule R, Groh M, Vallée A, Ackermann F, Group ftFC-S (2020) Interleukin-6 blockade for severe COVID-19. medRxiv: 2020.2004.2020.20061861 DOI 10.1101/2020.04.20.20061861

128 Narain S, Stefanov DG, Chau AS, Weber AG, Marder G, Kaplan B, Malhotra P, Bloom O, Liu A, Lesser ML, Hajizadeh N, Consortium NC-R (2020) Comparative Survival Analysis of Immunomodulatory Therapy for COVID-19 ‘Cytokine Storm’: A Retrospective Observational Cohort Study. medRxiv: 2020.2006.2016.20126714 DOI 10.1101/2020.06.16.20126714

129 Coomes EA, Haghbayan H (2020) Interleukin-6 in COVID-19: A Systematic Review and Meta-Analysis. medRxiv: 2020.2003.2030.20048058 DOI 10.1101/2020.03.30.20048058

130 Huang I, Pranata R, Lim MA, Oehadian A, Alisjahbana B (2020) C-reactive protein, procalcitonin, D-dimer, and ferritin in severe coronavirus disease-2019: a meta-analysis. Ther Adv Respir Dis 14: 1753466620937175 DOI 10.1177/1753466620937175

131 Hoffmann M, Hofmann-Winkler H, Smith JC, Kruger N, Arora P, Sorensen LK, Sogaard OS, Hasselstrom JB, Winkler M, Hempel T, Raich L, Olsson S, Danov O, Jonigk D, Yamazoe T, Yamatsuta K, Mizuno H, Ludwig S, Noe F, Kjolby M, Braun A, Sheltzer JM, Pohlmann S (2021) Camostat mesylate inhibits SARS-CoV-2 activation by TMPRSS2-related proteases and its metabolite GBPA exerts antiviral activity. EBioMedicine: 103255 DOI 10.1016/j.ebiom.2021.103255

132 Vultaggio A, Vivarelli E, Virgili G, Lucenteforte E, Bartoloni A, Nozzoli C, Morettini A, Berni A, Malandrino D, Rossi O, Nencini F, Pieralli F, Peris A, Lagi F, Scocchera G, Spinicci M, Trotta M, Mazzetti M, Parronchi P, Cosmi L, Liotta F, Fontanari P, Mazzoni A, Salvati L, Maggi E, Annunziato F, Almerigogna F, Matucci A (2020) Prompt Predicting of Early Clinical Deterioration of Moderate-to-Severe COVID-19 Patients: Usefulness of a Combined Score Using IL-6 in a Preliminary Study. J Allergy Clin Immunol Pract 8 (8): 2575-2581.e2572 DOI 10.1016/j.jaip.2020.06.013

133 Liu F, Li L, Xu M, Wu J, Luo D, Zhu Y, Li B, Song X, Zhou X (2020) Prognostic value of interleukin-6, C-reactive protein, and procalcitonin in patients with COVID-19. J Clin Virol 127: 104370 DOI 10.1016/j.jcv.2020.104370

134 Kosiborod M, Berwanger O, Koch GG, Martinez F, Mukhtar O, Verma S, Chopra V, Javaheri A, Ambery P, Gasparyan SB, Buenconsejo J, Sjöström CD, Langkilde AM, Oscarsson J, Esterline R (2021) Effects of dapagliflozin on prevention of major clinical events and recovery in patients with respiratory failure because of COVID-19: Design and rationale for the DARE-19 study. Diabetes Obes Metab 23 (4): 886-896 DOI 10.1111/dom.14296

135 Prussia A, Thepchatri P, Snyder JP, Plemper RK (2011) Systematic approaches towards the development of host-directed antiviral therapeutics. Int J Mol Sci 12 (6): 4027-4052 DOI 10.3390/ijms12064027

136 Hoffmann M, Zhang L, Krüger N, Graichen L, Kleine-Weber H, Hofmann-Winkler H, Kempf A, Nessler S, Riggert J, Winkler MS, Schulz S, Jäck H-M, Pöhlmann S (2021) SARS-CoV-2 mutations acquired in mink reduce antibody-mediated neutralization. bioRxiv: 2021.2002.2012.430998 DOI 10.1101/2021.02.12.430998

137 Schuler BA, Habermann AC, Plosa EJ, Taylor CJ, Jetter C, Negretti NM, Kapp ME, Benjamin JT, Gulleman P, Nichols DS, Braunstein LZ, Hackett A, Koval M, Guttentag SH, Blackwell TS, Webber SA, Banovich NE, Vanderbilt C-CC, Human Cell Atlas Biological N, Kropski JA, Sucre JM (2021) Age-determined expression of priming protease TMPRSS2 and localization of SARS-CoV-2 in lung epithelium. J Clin Invest 131 (1) DOI 10.1172/JCI140766

138 (assessed 20th April, 2021) <https://www.merck.com/news/merck-and-ridgeback-biotherapeutics-provide-update-on-progress-of-clinical-development-program-for-molnupiravir-an-investigational-oral-therapeutic-for-the-treatment-of-mild-to-moderate-covid-19/>. In: ed. Merck.

139 Yu X, Sun S, Shi Y, Wang H, Zhao R, Sheng J (2020) SARS-CoV-2 viral load in sputum correlates with risk of COVID-19 progression. Crit Care 24 (1): 170 DOI 10.1186/s13054-020-02893-8

140 Wang D, Zou L, Jin Q, Hou J, Ge G, Yang L (2018) Human carboxylesterases: a comprehensive review. Acta Pharm Sin B 8 (5): 699-712 DOI 10.1016/j.apsb.2018.05.005

141 Mackness B, Durrington PN, Mackness MI (1998) Human serum paraoxonase. Gen Pharmacol 31 (3): 329-336 DOI 10.1016/s0306-3623(98)00028-7

142 Di L (2019) The Impact of Carboxylesterases in Drug Metabolism and Pharmacokinetics. Curr Drug Metab 20 (2): 91-102 DOI 10.2174/1389200219666180821094502

143 Almarzooq AA (2021) Exploration of interethnic variation and repurposed drug efficacy in the treatment of SARS-CoV-2 Infection (COVID-19). medRxiv: 2021.2003.2007.21253095 DOI 10.1101/2021.03.07.21253095

144 McCreary EK, Pogue JM (2020) Coronavirus Disease 2019 Treatment: A Review of Early and Emerging Options. Open Forum Infect Dis 7 (4): ofaa105 DOI 10.1093/ofid/ofaa105

145 Sortica VA, Lindenau JD, Cunha MG, Ohnishi MD, Ventura AMR, Ribeiro-Dos-Santos Â K, Santos SE, Guimarães LS, Hutz MH (2016) The effect of SNPs in CYP450 in chloroquine/primaquine Plasmodium vivax malaria treatment. Pharmacogenomics 17 (17): 1903-1911 DOI 10.2217/pgs-2016-0131

146 Elewa H, Wilby KJ (2017) A Review of Pharmacogenetics of Antimalarials and Associated Clinical Implications. Eur J Drug Metab Pharmacokinet 42 (5): 745-756 DOI 10.1007/s13318-016-0399-1

147 Takahashi T, Luzum JA, Nicol MR, Jacobson PA (2020) Pharmacogenomics of COVID-19 therapies. NPJ Genom Med 5: 35 DOI 10.1038/s41525-020-00143-y

148 Eriksson JW, Lundkvist P, Jansson PA, Johansson L, Kvarnström M, Moris L, Miliotis T, Forsberg GB, Risérus U, Lind L, Oscarsson J (2018) Effects of dapagliflozin and n-3 carboxylic acids on non-alcoholic fatty liver disease in people with type 2 diabetes: a double-blind randomised placebo-controlled study. Diabetologia 61 (9): 1923-1934 DOI 10.1007/s00125-018-4675-2

149 Pereira MJ, Lundkvist P, Kamble PG, Lau J, Martins JG, Sjöström CD, Schnecke V, Walentinsson A, Johnsson E, Eriksson JW (2018) A Randomized Controlled Trial of Dapagliflozin Plus Once-Weekly Exenatide Versus Placebo in Individuals with Obesity and Without Diabetes: Metabolic Effects and Markers Associated with Bodyweight Loss. Diabetes Ther 9 (4): 1511-1532 DOI 10.1007/s13300-018-0449-6
