# Supplementary material for: Translatability scoring in prospective and retrospective COVID drug development cases
Source: Eur J Clin Pharmacol. 2023 Jun 6;79(8):1051–71. doi: 10.1007/s00228-023-03517-0 (PMC10243273; doi:10.1007/s00228-023-03517-0)
Supplement: Supplementary file 3 — Supplementary file3 (DOCX 152 kb) [file 228_2023_3517_MOESM3_ESM.docx]

**Supplementary table 3: Biomarker scoring for vaccines**

|  | **virus neutralizing titer, VNT** | | | | | **IgG spike protein, ELISA** | | | | |
| --- | --- | --- | --- | --- | --- | --- | --- | --- | --- | --- |
| Points to evaluate | **CVnCoV**  **(Curevac)** | **Covifenz**  **(Medicago)** | **Vidprevtyn**  **(Sanofi Pasteur)** | **Vaxzevria**  **(Astra Zeneca)** | **Comirnaty**  **(Biontech)** | **CVnCoV**  **(Curevac)** | **Covifenz**  **(Medicago)** | **Vidprevtyn**  **(Sanofi Pasteur)** | **Vaxzevria**  **(Astra Zeneca)** | **Comirnaty**  **(Biontech)** |
| 1 (animal, in-vitro data) | 5 [1-3] | 5 [1-3] | 5 [1-3] | 5 [4-6] | 5 [4-6] | 5 [1] [2, 3] | 5 [1-3] | 5 [1-3] | 5 [1, 6, 7] | 5 [1, 6, 7] |
| 2 (how many species) | 5 [2, 3] | 5 [2, 3, 8] | 5 [2, 3, 8] | 5 [4-6] | 5 [4-6] | 5 [2, 3, 8] | 5 [2, 3, 8] | 5 [2, 3, 8] | 5 [4-6] | 5 [4-6] |
| 3 (suitable animal models) | 4 [9] | 4 [9] | 4 [9] | 4 [9] | 4 [9] | 4 [9] | 4 [9] | 4 [9] | 4 [9] | 4 [9] |
| 4 (clinical data) | 5 [5] | 5 [5] | 5 [5] | 5 [1, 6] | 5 [1, 6] | 5 [5] | 5 [5] | 5 [5] | 5 [1, 6] | 5 [1, 6] |
| 5 (human data) | 5 [5, 10] | 5 [5, 11] | 5 [5, 11-13] | 5 [1, 6, 14] | 5 [1, 6, 14] | 5 [5, 10] | 5 [5, 11] | 5 [5, 11-13] | 5 [1, 6] | 5 [1, 6] |
| 6 (human data classification) | 5 [5, 10] | 5 (10) [11] | 5 (10) [11, 13] | 4 (8) [10] | 4 (8) [10] | 5 [5, 10] | 5 (10) [11] | 5 (10) [11, 13] | 4 (8) [10] | 4 (8) [10] |
| 7 (biomarker important for disease) | 4 [15] | 4 [15] | 4 [15] | 3 [16] | 3 [16] | 4 [17] | 4 [17] | 4 [17] | 3 [16] | 3 [16] |
| 8 (statistical predictability) | 3 [18, 19] | 3 [18, 19] | 3 [18, 19] | 3 [18] | 3 [18] | 5 [20][)](https://www.news-medical.net/news/20210122/Accurate-quantitative-COVID-19-antibody-test-kit-by-EKF.aspx) | 5 [21] | 5 [21] | 5 [20] | 5 [20] |
| 9 (accuracy or reproducibility) | 3 [18, 19] | 3 [18, 19] | 3 [18, 19] | 3 [18] | 3 [18] | 5 [20] | 5 [21] | 5 [21] | 5 [20] | 5 [20] |
| 10 (accessibility) | 5 [22] | 5 [22] | 5 [22] | 5 [22] | 5 [22] | 5 [2] | 5 [11] | 5 [11] | 5 [5, 7] | 5 [5, 7] |
| Sum | **49** | **49** | **49** | **46** | **46** | **53** | **53** | **53** | **50** | **50** |

Points to evaluate:

1. Are animal or in vitro data available?
2. How many species have been tested positively?
3. Are the animal models enough to reflect human disease?
4. Is there corresponding clinical data?
5. Are human data available?
6. Human data classification (2x)
7. Does the biomarker represent a pivotal disease constituent?
8. What is the statistical predictability?
9. What is the accuracy or reproducibility of the assay?
10. How accessible is the specimen?

**References:**

1 Corbett KS, Edwards DK, Leist SR, Abiona OM, Boyoglu-Barnum S, Gillespie RA, Himansu S, Schäfer A, Ziwawo CT, DiPiazza AT, Dinnon KH, Elbashir SM, Shaw CA, Woods A, Fritch EJ, Martinez DR, Bock KW, Minai M, Nagata BM, Hutchinson GB, Wu K, Henry C, Bahl K, Garcia-Dominguez D, Ma L, Renzi I, Kong W-P, Schmidt SD, Wang L, Zhang Y, Phung E, Chang LA, Loomis RJ, Altaras NE, Narayanan E, Metkar M, Presnyak V, Liu C, Louder MK, Shi W, Leung K, Yang ES, West A, Gully KL, Stevens LJ, Wang N, Wrapp D, Doria-Rose NA, Stewart-Jones G, Bennett H, Alvarado GS, Nason MC, Ruckwardt TJ, McLellan JS, Denison MR, Chappell JD, Moore IN, Morabito KM, Mascola JR, Baric RS, Carfi A, Graham BS (2020) SARS-CoV-2 mRNA vaccine design enabled by prototype pathogen preparedness. Nature 586 (7830): 567-571 DOI 10.1038/s41586-020-2622-0

2 Rauch S, Gooch K, Hall Y, Salguero FJ, Dennis MJ, Gleeson FV, Harris D, Ho C, Humphries HE, Longet S, Ngabo D, Paterson J, Rayner EL, Ryan KA, Sharpe S, Watson RJ, Mueller SO, Petsch B, Carroll MW (2020) mRNA vaccine CVnCoV protects non-human primates from SARS-CoV-2 challenge infection. bioRxiv: 2020.2012.2023.424138 DOI 10.1101/2020.12.23.424138

3 Rauch S, Roth N, Schwendt K, Fotin-Mleczek M, Mueller SO, Petsch B (2021) mRNA based SARS-CoV-2 vaccine candidate CVnCoV induces high levels of virus neutralizing antibodies and mediates protection in rodents. bioRxiv: 2020.2010.2023.351775 DOI 10.1101/2020.10.23.351775

4 Vogel AB, Kanevsky I, Che Y, Swanson KA, Muik A, Vormehr M, Kranz LM, Walzer KC, Hein S, Güler A, Loschko J, Maddur MS, Ota-Setlik A, Tompkins K, Cole J, Lui BG, Ziegenhals T, Plaschke A, Eisel D, Dany SC, Fesser S, Erbar S, Bates F, Schneider D, Jesionek B, Sänger B, Wallisch AK, Feuchter Y, Junginger H, Krumm SA, Heinen AP, Adams-Quack P, Schlereth J, Schille S, Kröner C, de la Caridad Güimil Garcia R, Hiller T, Fischer L, Sellers RS, Choudhary S, Gonzalez O, Vascotto F, Gutman MR, Fontenot JA, Hall-Ursone S, Brasky K, Griffor MC, Han S, Su AAH, Lees JA, Nedoma NL, Mashalidis EH, Sahasrabudhe PV, Tan CY, Pavliakova D, Singh G, Fontes-Garfias C, Pride M, Scully IL, Ciolino T, Obregon J, Gazi M, Carrion R, Jr., Alfson KJ, Kalina WV, Kaushal D, Shi PY, Klamp T, Rosenbaum C, Kuhn AN, Türeci Ö, Dormitzer PR, Jansen KU, Sahin U (2021) BNT162b vaccines protect rhesus macaques from SARS-CoV-2. Nature 592 (7853): 283-289 DOI 10.1038/s41586-021-03275-y

5 Walsh EE, Frenck RW, Jr., Falsey AR, Kitchin N, Absalon J, Gurtman A, Lockhart S, Neuzil K, Mulligan MJ, Bailey R, Swanson KA, Li P, Koury K, Kalina W, Cooper D, Fontes-Garfias C, Shi PY, Tureci O, Tompkins KR, Lyke KE, Raabe V, Dormitzer PR, Jansen KU, Sahin U, Gruber WC (2020) Safety and Immunogenicity of Two RNA-Based Covid-19 Vaccine Candidates. N Engl J Med 383 (25): 2439-2450 DOI 10.1056/NEJMoa2027906

6 van Doremalen N, Lambe T, Spencer A, Belij-Rammerstorfer S, Purushotham JN, Port JR, Avanzato VA, Bushmaker T, Flaxman A, Ulaszewska M, Feldmann F, Allen ER, Sharpe H, Schulz J, Holbrook M, Okumura A, Meade-White K, Pérez-Pérez L, Edwards NJ, Wright D, Bissett C, Gilbride C, Williamson BN, Rosenke R, Long D, Ishwarbhai A, Kailath R, Rose L, Morris S, Powers C, Lovaglio J, Hanley PW, Scott D, Saturday G, de Wit E, Gilbert SC, Munster VJ (2020) ChAdOx1 nCoV-19 vaccine prevents SARS-CoV-2 pneumonia in rhesus macaques. Nature 586 (7830): 578-582 DOI 10.1038/s41586-020-2608-y

7 Vogel AB, Kanevsky I, Che Y, Swanson KA, Muik A, Vormehr M, Kranz LM, Walzer KC, Hein S, Güler A, Loschko J, Maddur MS, Ota-Setlik A, Tompkins K, Cole J, Lui BG, Ziegenhals T, Plaschke A, Eisel D, Dany SC, Fesser S, Erbar S, Bates F, Schneider D, Jesionek B, Sänger B, Wallisch A-K, Feuchter Y, Junginger H, Krumm SA, Heinen AP, Adams-Quack P, Schlereth J, Schille S, Kröner C, de la Caridad Güimil Garcia R, Hiller T, Fischer L, Sellers RS, Choudhary S, Gonzalez O, Vascotto F, Gutman MR, Fontenot JA, Hall-Ursone S, Brasky K, Griffor MC, Han S, Su AAH, Lees JA, Nedoma NL, Mashalidis EH, Sahasrabudhe PV, Tan CY, Pavliakova D, Singh G, Fontes-Garfias C, Pride M, Scully IL, Ciolino T, Obregon J, Gazi M, Carrion R, Alfson KJ, Kalina WV, Kaushal D, Shi P-Y, Klamp T, Rosenbaum C, Kuhn AN, Türeci Ö, Dormitzer PR, Jansen KU, Sahin U (2021) BNT162b vaccines protect rhesus macaques from SARS-CoV-2. Nature DOI 10.1038/s41586-021-03275-y

8 Kremsner P, Mann P, Bosch J, Fendel R, Gabor JJ, Kreidenweiss A, Kroidl A, Leroux-Roels I, Leroux-Roels G, Schindler C, Schunk M, Velavan TP, Fotin-Mleczek M, Müller S, Quintini G, Schönborn-Kellenberger O, Vahrenhorst D, Verstraeten T, Walz L, Wolz O-O, Oostvogels L (2020) Phase 1 Assessment of the Safety and Immunogenicity of an mRNA- Lipid Nanoparticle Vaccine Candidate Against SARS-CoV-2 in Human Volunteers. medRxiv: 2020.2011.2009.20228551 DOI 10.1101/2020.11.09.20228551

9 Muñoz-Fontela C, Dowling WE, Funnell SGP, Gsell P-S, Riveros-Balta AX, Albrecht RA, Andersen H, Baric RS, Carroll MW, Cavaleri M, Qin C, Crozier I, Dallmeier K, de Waal L, de Wit E, Delang L, Dohm E, Duprex WP, Falzarano D, Finch CL, Frieman MB, Graham BS, Gralinski LE, Guilfoyle K, Haagmans BL, Hamilton GA, Hartman AL, Herfst S, Kaptein SJF, Klimstra WB, Knezevic I, Krause PR, Kuhn JH, Le Grand R, Lewis MG, Liu W-C, Maisonnasse P, McElroy AK, Munster V, Oreshkova N, Rasmussen AL, Rocha-Pereira J, Rockx B, Rodríguez E, Rogers TF, Salguero FJ, Schotsaert M, Stittelaar KJ, Thibaut HJ, Tseng C-T, Vergara-Alert J, Beer M, Brasel T, Chan JFW, García-Sastre A, Neyts J, Perlman S, Reed DS, Richt JA, Roy CJ, Segalés J, Vasan SS, Henao-Restrepo AM, Barouch DH (2020) Animal models for COVID-19. Nature 586 (7830): 509-515 DOI 10.1038/s41586-020-2787-6

10 Jackson LA, Anderson EJ, Rouphael NG, Roberts PC, Makhene M, Coler RN, McCullough MP, Chappell JD, Denison MR, Stevens LJ, Pruijssers AJ, McDermott A, Flach B, Doria-Rose NA, Corbett KS, Morabito KM, O'Dell S, Schmidt SD, Swanson PA, 2nd, Padilla M, Mascola JR, Neuzil KM, Bennett H, Sun W, Peters E, Makowski M, Albert J, Cross K, Buchanan W, Pikaart-Tautges R, Ledgerwood JE, Graham BS, Beigel JH, m RNASG (2020) An mRNA Vaccine against SARS-CoV-2 - Preliminary Report. N Engl J Med 383 (20): 1920-1931 DOI 10.1056/NEJMoa2022483

11 Ward BJ, Gobeil P, Séguin A, Atkins J, Boulay I, Charbonneau P-Y, Couture M, D’Aoust M-A, Dhaliwall J, Finkle C, Hager K, Mahmood A, Makarkov A, Cheng M, Pillet S, Schimke P, St-Martin S, Trépanier S, Landry N (2020) Phase 1 trial of a Candidate Recombinant Virus-Like Particle Vaccine for Covid-19 Disease Produced in Plants. medRxiv: 2020.2011.2004.20226282 DOI 10.1101/2020.11.04.20226282

12 Francica JR, Flynn BJ, Foulds KE, Noe AT, Werner AP, Moore IN, Gagne M, Johnston TS, Tucker C, Davis RL, Flach B, O’Connell S, Andrew SF, Lamb E, Flebbe DR, Nurmukhambetova ST, Donaldson MM, Todd J-PM, Zhu AL, Atyeo C, Fischinger S, Gorman MJ, Shin S, Edara VV, Floyd K, Lai L, Tylor A, McCarthy E, Lecouturier V, Ruiz S, Berry C, Tibbitts T, Andersen H, Cook A, Dodson A, Pessaint L, Ry AV, Koutsoukos M, Gutzeit C, Teng I-T, Zhou T, Li D, Haynes BF, Kwong PD, McDermott A, Lewis MG, Fu TM, Chicz R, van der Most R, Corbett KS, Suthar MS, Alter G, Roederer M, Sullivan NJ, Douek DC, Graham BS, Casimiro D, Seder RA (2021) Vaccination with SARS-CoV-2 Spike Protein and AS03 Adjuvant Induces Rapid Anamnestic Antibodies in the Lung and Protects Against Virus Challenge in Nonhuman Primates. bioRxiv: 2021.2003.2002.433390 DOI 10.1101/2021.03.02.433390

13 Goepfert PA, Fu B, Chabanon AL, Bonaparte MI, Davis MG, Essink BJ, Frank I, Haney O, Janosczyk H, Keefer MC, Koutsoukos M, Kimmel MA, Masotti R, Savarino SJ, Schuerman L, Schwartz H, Sher LD, Smith J, Tavares-Da-Silva F, Gurunathan S, DiazGranados CA, de Bruyn G (2021) Safety and immunogenicity of SARS-CoV-2 recombinant protein vaccine formulations in healthy adults: interim results of a randomised, placebo-controlled, phase 1-2, dose-ranging study. Lancet Infect Dis DOI 10.1016/S1473-3099(21)00147-X

14 Suthar MS, Zimmerman MG, Kauffman RC, Mantus G, Linderman SL, Hudson WH, Vanderheiden A, Nyhoff L, Davis CW, Adekunle O, Affer M, Sherman M, Reynolds S, Verkerke HP, Alter DN, Guarner J, Bryksin J, Horwath MC, Arthur CM, Saakadze N, Smith GH, Edupuganti S, Scherer EM, Hellmeister K, Cheng A, Morales JA, Neish AS, Stowell SR, Frank F, Ortlund E, Anderson EJ, Menachery VD, Rouphael N, Mehta AK, Stephens DS, Ahmed R, Roback JD, Wrammert J (2020) Rapid Generation of Neutralizing Antibody Responses in COVID-19 Patients. Cell Rep Med 1 (3): 100040 DOI 10.1016/j.xcrm.2020.100040

15 Lau EHY, Tsang OTY, Hui DSC, Kwan MYW, Chan W-h, Chiu SS, Ko RLW, Chan KH, Cheng SMS, Perera RAPM, Cowling BJ, Poon LLM, Peiris M (2021) Neutralizing antibody titres in SARS-CoV-2 infections. Nature Communications 12 (1): 63 DOI 10.1038/s41467-020-20247-4

16 Ju B, Zhang Q, Ge J, Wang R, Sun J, Ge X, Yu J, Shan S, Zhou B, Song S, Tang X, Yu J, Lan J, Yuan J, Wang H, Zhao J, Zhang S, Wang Y, Shi X, Liu L, Zhao J, Wang X, Zhang Z, Zhang L (2020) Human neutralizing antibodies elicited by SARS-CoV-2 infection. Nature 584 (7819): 115-119 DOI 10.1038/s41586-020-2380-z

17 Nag DS, Chaudhry R, Mishra M, Rai S, Gupta M (2020) A Prospective Study on Rapidly Declining SARS-CoV-2 IgG Antibodies Within One to Three Months of Testing IgG Positive: Can It Lead to Potential Reinfections? Cureus 12 (12): e11845 DOI 10.7759/cureus.11845

18 Whiteman MC, Bogardus L, Giacone DG, Rubinstein LJ, Antonello JM, Sun D, Daijogo S, Gurney KB (2018) Virus Reduction Neutralization Test: A Single-Cell Imaging High-Throughput Virus Neutralization Assay for Dengue. Am J Trop Med Hyg 99 (6): 1430-1439 DOI 10.4269/ajtmh.17-0948

19 Rathe JA, Hemann EA, Eggenberger J, Li Z, Knoll ML, Stokes C, Hsiang TY, Netland J, Takehara KK, Pepper M, Gale M (2020) SARS-CoV-2 Serologic Assays in Control and Unknown Populations Demonstrate the Necessity of Virus Neutralization Testing. J Infect Dis DOI 10.1093/infdis/jiaa797

20 Klumpp-Thomas C, Kalish H, Drew M, Hunsberger S, Snead K, Fay MP, Mehalko J, Shunmugavel A, Wall V, Frank P, Denson JP, Hong M, Gulten G, Messing S, Hicks J, Michael S, Gillette W, Hall MD, Memoli M, Esposito D, Sadtler K (2020) Standardization of enzyme-linked immunosorbent assays for serosurveys of the SARS-CoV-2 pandemic using clinical and at-home blood sampling. medRxiv DOI 10.1101/2020.05.21.20109280

21 Klumpp-Thomas C, Kalish H, Drew M, Hunsberger S, Snead K, Fay MP, Mehalko J, Shunmugavel A, Wall V, Frank P, Denson J-P, Hong M, Gulten G, Messing S, Hicks J, Michael S, Gillette W, Hall MD, Memoli MJ, Esposito D, Sadtler K (2021) Standardization of ELISA protocols for serosurveys of the SARS-CoV-2 pandemic using clinical and at-home blood sampling. Nature Communications 12 (1): 113 DOI 10.1038/s41467-020-20383-x

22 Amanat F, White KM, Miorin L, Strohmeier S, McMahon M, Meade P, Liu W-C, Albrecht RA, Simon V, Martinez-Sobrido L, Moran T, García-Sastre A, Krammer F (2020) An In Vitro Microneutralization Assay for SARS-CoV-2 Serology and Drug Screening. Current Protocols in Microbiology 58 (1): e108 DOI <https://doi.org/10.1002/cpmc.108>
