# Supplementary material for: Translatability scoring in prospective and retrospective COVID drug development cases
Source: Eur J Clin Pharmacol. 2023 Jun 6;79(8):1051–71. doi: 10.1007/s00228-023-03517-0 (PMC10243273; doi:10.1007/s00228-023-03517-0)
Supplement: Supplementary file 4 — Supplementary file4 (DOCX 126 kb) [file 228_2023_3517_MOESM4_ESM.docx]

**Supplementary table 4: Biomarker scoring for drugs to treat COVID**

|  | **viral load dynamics in respiratory samples, determined by real time RT-PCR** | | | | | **CRP** | **IL-6** |
| --- | --- | --- | --- | --- | --- | --- | --- |
| Points to evaluate | **camostat mesylate** | **molnupiravir** | **fluvoxamine** | **remdesivir** | **hydroxy-**  **chloroquine** | **tocilizumab** | **dapagliflozin** |
| 1 (animal, in-vitro data) | 5 [1] [2-4] | 5 [1] [2-4] | 5 [1] [2-4] | 5 [5-7] | 4 [8, 9] | 5 [8] | 5 [10-12] |
| 2 (how many species) | 5 [1-4] | 5 [1-4] | 5 [1-4] | 5 [7, 13] | 5 [3, 8, 9] | 5 [14-16] | 3 [11] |
| 3 (suitable animal models) | 3 [2, 3] | 3 [2, 3] | 3 [2, 3] | 3 [17, 18] | 2 [3] | 4 [15, 19] | 4 [11] |
| 4 (clinical data) | 2 [20] | 3 [20] | 2 [20] | 2 [13, 21] | 4 [3] | 5 [16] | 5 [10, 12] |
| 5 (human data) | 1 | 5 [22] | 1 | 1 | 5 [23] | 5 [16, 24, 25] | 5 [26] |
| 6 (human data classification) | 2 (4) [20] | 3 (6) [20, 22, 27, 28] | 3 (6) [20, 27, 28] | 1 (2) [29] | 6 [23] | 12 [24, 25, 30, 31] | 10 [10] |
| 7 (biomarker important for disease) | 2 [32] | 2 [32] | 2 [32] | 2 [21, 33, 34] | 2 | 4 [30, 35] | 3 [10, 11] |
| 8 (statistical predictability) | 3 [36, 37] | 3 [36, 37] | 3 [36, 37] | 3 [38] | 2 [39] | 5 [40] | 5 [41] |
| 9 (accuracy or reproducibility) | 3 [36, 37] | 3 [36, 37] | 3 [36, 37] | 3 [38] | 2 | 5 [42] | 5 [41] |
| 10 (accessibility) | 3 [1, 3] | 3 [1, 3] | 3 [1, 3] | 3 [7, 43] | 3 | 5 [44] | 5 |
| Sum | **31** | **38** | **33** | **29** | **35** | **55** | **50** |

Points to evaluate:

1. Are animal or in vitro data available?
2. How many species have been tested positively?
3. Are the animal models enough to reflect human disease?
4. Is there corresponding clinical data?
5. Are human data available?
6. Human data classification (2x)
7. Does the biomarker represent a pivotal disease constituent?
8. What is the statistical predictability?
9. What is the accuracy or reproducibility of the assay?
10. How accessible is the specimen?

**References:**

1 Maisonnasse P, Guedj J, Contreras V, Behillil S, Solas C, Marlin R, Naninck T, Pizzorno A, Lemaitre J, Gonçalves A, Kahlaoui N, Terrier O, Fang RHT, Enouf V, Dereuddre-Bosquet N, Brisebarre A, Touret F, Chapon C, Hoen B, Lina B, Calatrava MR, van der Werf S, de Lamballerie X, Le Grand R (2020) Hydroxychloroquine use against SARS-CoV-2 infection in non-human primates. Nature 585 (7826): 584-587 DOI 10.1038/s41586-020-2558-4

2 Wang J, Shuai L, Wang C, Liu R, He X, Zhang X, Sun Z, Shan D, Ge J, Wang X, Hua R, Zhong G, Wen Z, Bu Z (2020) Mouse-adapted SARS-CoV-2 replicates efficiently in the upper and lower respiratory tract of BALB/c and C57BL/6J mice. Protein Cell 11 (10): 776-782 DOI 10.1007/s13238-020-00767-x

3 Kleiboeker S, Cowden S, Grantham J, Nutt J, Tyler A, Berg A, Altrich M (2020) SARS-CoV-2 viral load assessment in respiratory samples. J Clin Virol 129: 104439 DOI 10.1016/j.jcv.2020.104439

4 Hassan AO, Case JB, Winkler ES, Thackray LB, Kafai NM, Bailey AL, McCune BT, Fox JM, Chen RE, Alsoussi WB, Turner JS, Schmitz AJ, Lei T, Shrihari S, Keeler SP, Fremont DH, Greco S, McCray PB, Jr., Perlman S, Holtzman MJ, Ellebedy AH, Diamond MS (2020) A SARS-CoV-2 Infection Model in Mice Demonstrates Protection by Neutralizing Antibodies. Cell 182 (3): 744-753 e744 DOI 10.1016/j.cell.2020.06.011

5 Pruijssers AJ, George AS, Schäfer A, Leist SR, Gralinksi LE, Dinnon KH, Yount BL, Agostini ML, Stevens LJ, Chappell JD, Lu X, Hughes TM, Gully K, Martinez DR, Brown AJ, Graham RL, Perry JK, Du Pont V, Pitts J, Ma B, Babusis D, Murakami E, Feng JY, Bilello JP, Porter DP, Cihlar T, Baric RS, Denison MR, Sheahan TP (2020) Remdesivir potently inhibits SARS-CoV-2 in human lung cells and chimeric SARS-CoV expressing the SARS-CoV-2 RNA polymerase in mice. bioRxiv DOI 10.1101/2020.04.27.064279

6 Brown AJ, Won JJ, Graham RL, Dinnon KH, 3rd, Sims AC, Feng JY, Cihlar T, Denison MR, Baric RS, Sheahan TP (2019) Broad spectrum antiviral remdesivir inhibits human endemic and zoonotic deltacoronaviruses with a highly divergent RNA dependent RNA polymerase. Antiviral Res 169: 104541 DOI 10.1016/j.antiviral.2019.104541

7 Williamson BN, Feldmann F, Schwarz B, Meade-White K, Porter DP, Schulz J, van Doremalen N, Leighton I, Kwe Yinda C, Pérez-Pérez L, Okumura A, Lovaglio J, Hanley PW, Saturday G, Bosio CM, Anzick S, Barbian K, Cihlar T, Martens C, Scott DP, Munster VJ, de Wit E (2020) Clinical benefit of remdesivir in rhesus macaques infected with SARS-CoV-2. bioRxiv DOI 10.1101/2020.04.15.043166

8 Woolsey C, Borisevich V, Prasad AN, Agans KN, Deer DJ, Dobias NS, Heymann JC, Foster SL, Levine CB, Medina L, Melody K, Geisbert JB, Fenton KA, Geisbert TW, Cross RW (2020) Establishment of an African green monkey model for COVID-19. bioRxiv: 2020.2005.2017.100289 DOI 10.1101/2020.05.17.100289

9 Munster VJ, Feldmann F, Williamson BN, van Doremalen N, Pérez-Pérez L, Schulz J, Meade-White K, Okumura A, Callison J, Brumbaugh B, Avanzato VA, Rosenke R, Hanley PW, Saturday G, Scott D, Fischer ER, de Wit E (2020) Respiratory disease and virus shedding in rhesus macaques inoculated with SARS-CoV-2. bioRxiv: 2020.2003.2021.001628 DOI 10.1101/2020.03.21.001628

10 Tang Y, Liu J, Zhang D, Xu Z, Ji J, Wen C (2020) Cytokine Storm in COVID-19: The Current Evidence and Treatment Strategies. Front Immunol 11: 1708 DOI 10.3389/fimmu.2020.01708

11 Magro G (2020) SARS-CoV-2 and COVID-19: Is interleukin-6 (IL-6) the 'culprit lesion' of ARDS onset? What is there besides Tocilizumab? SGP130Fc. Cytokine X 2 (2): 100029 DOI 10.1016/j.cytox.2020.100029

12 Gubernatorova EO, Gorshkova EA, Polinova AI, Drutskaya MS (2020) IL-6: Relevance for immunopathology of SARS-CoV-2. Cytokine Growth Factor Rev 53: 13-24 DOI 10.1016/j.cytogfr.2020.05.009

13 Pan Y, Zhang D, Yang P, Poon LLM, Wang Q (2020) Viral load of SARS-CoV-2 in clinical samples. Lancet Infect Dis 20 (4): 411-412 DOI 10.1016/s1473-3099(20)30113-4

14 Weinhold B, Rüther U (1997) Interleukin-6-dependent and -independent regulation of the human C-reactive protein gene. Biochem J 327 ( Pt 2) (Pt 2): 425-429 DOI 10.1042/bj3270425

15 Torzewski M, Waqar AB, Fan J (2014) Animal models of C-reactive protein. Mediators Inflamm 2014: 683598 DOI 10.1155/2014/683598

16 Perrone F, Piccirillo MC, Ascierto PA, Salvarani C, Parrella R, Marata AM, Popoli P, Ferraris L, Marrocco-Trischitta MM, Ripamonti D, Binda F, Bonfanti P, Squillace N, Castelli F, Muiesan ML, Lichtner M, Calzetti C, Salerno ND, Atripaldi L, Cascella M, Costantini M, Dolci G, Facciolongo NC, Fraganza F, Massari M, Montesarchio V, Mussini C, Negri EA, Botti G, Cardone C, Gargiulo P, Gravina A, Schettino C, Arenare L, Chiodini P, Gallo C, on behalf of the TOCIVID-19 investigators I (2020) Tocilizumab for patients with COVID-19 pneumonia. The TOCIVID-19 prospective phase 2 trial. medRxiv: 2020.2006.2001.20119149 DOI 10.1101/2020.06.01.20119149

17 Yu P, Qi F, Xu Y, Li F, Liu P, Liu J, Bao L, Deng W, Gao H, Xiang Z, Xiao C, Lv Q, Gong S, Liu J, Song Z, Qu Y, Xue J, Wei Q, Liu M, Wang G, Wang S, Yu H, Liu X, Huang B, Wang W, Zhao L, Wang H, Ye F, Zhou W, Zhen W, Han J, Wu G, Jin Q, Wang J, Tan W, Qin C (2020) Age-related rhesus macaque models of COVID-19. Animal Model Exp Med 3 (1): 93-97 DOI 10.1002/ame2.12108

18 Kim YI, Kim SG, Kim SM, Kim EH, Park SJ, Yu KM, Chang JH, Kim EJ, Lee S, Casel MAB, Um J, Song MS, Jeong HW, Lai VD, Kim Y, Chin BS, Park JS, Chung KH, Foo SS, Poo H, Mo IP, Lee OJ, Webby RJ, Jung JU, Choi YK (2020) Infection and Rapid Transmission of SARS-CoV-2 in Ferrets. Cell Host Microbe 27 (5): 704-709.e702 DOI 10.1016/j.chom.2020.03.023

19 Szalai AJ, McCrory MA (2002) Varied biologic functions of C-reactive protein: lessons learned from transgenic mice. Immunol Res 26 (1-3): 279-287 DOI 10.1385/ir:26:1-3:279

20 Walsh KA, Jordan K, Clyne B, Rohde D, Drummond L, Byrne P, Ahern S, Carty PG, O'Brien KK, O'Murchu E, O'Neill M, Smith SM, Ryan M, Harrington P (2020) SARS-CoV-2 detection, viral load and infectivity over the course of an infection. J Infect 81 (3): 357-371 DOI 10.1016/j.jinf.2020.06.067

21 Liu Y, Yan LM, Wan L, Xiang TX, Le A, Liu JM, Peiris M, Poon LLM, Zhang W (2020) Viral dynamics in mild and severe cases of COVID-19. Lancet Infect Dis 20 (6): 656-657 DOI 10.1016/s1473-3099(20)30232-2

22 Fischer W, Eron JJ, Holman W, Cohen MS, Fang L, Szewczyk LJ, Sheahan TP, Baric R, Mollan KR, Wolfe CR, Duke ER, Azizad MM, Borroto-Esoda K, Wohl DA, Loftis AJ, Alabanza P, Lipansky F, Painter WP (2021) Molnupiravir, an Oral Antiviral Treatment for COVID-19. medRxiv: 2021.2006.2017.21258639 DOI 10.1101/2021.06.17.21258639

23 Gautret P, Lagier J-C, Parola P, Hoang VT, Meddeb L, Mailhe M, Doudier B, Courjon J, Giordanengo V, Vieira VE, Dupont HT, Honoré S, Colson P, Chabrière E, Scola BL, Rolain J-M, Brouqui P, Raoult D (2020) Hydroxychloroquine and azithromycin as a treatment of COVID-19: results of an open-label non-randomized clinical trial. medRxiv: 2020.2003.2016.20037135 DOI 10.1101/2020.03.16.20037135

24 Schultz DR, Arnold PI (1990) Properties of four acute phase proteins: C-reactive protein, serum amyloid A protein, alpha 1-acid glycoprotein, and fibrinogen. Semin Arthritis Rheum 20 (3): 129-147 DOI 10.1016/0049-0172(90)90055-k

25 Pepys MB, Hirschfield GM (2003) C-reactive protein: a critical update. J Clin Invest 111 (12): 1805-1812 DOI 10.1172/jci18921

26 Liu T, Zhang J, Yang Y, Ma H, Li Z, Zhang J, Cheng J, Zhang X, Zhao Y, Xia Z, Zhang L, Wu G, Yi J (2020) The role of interleukin-6 in monitoring severe case of coronavirus disease 2019. EMBO Mol Med 12 (7): e12421 DOI 10.15252/emmm.202012421

27 Udwadia ZF, Singh P, Barkate H, Patil S, Rangwala S, Pendse A, Kadam J, Wu W, Caracta CF, Tandon M (2021) Efficacy and safety of favipiravir, an oral RNA-dependent RNA polymerase inhibitor, in mild-to-moderate COVID-19: A randomized, comparative, open-label, multicenter, phase 3 clinical trial. Int J Infect Dis 103: 62-71 DOI 10.1016/j.ijid.2020.11.142

28 Gottlieb RL, Nirula A, Chen P, Boscia J, Heller B, Morris J, Huhn G, Cardona J, Mocherla B, Stosor V, Shawa I, Kumar P, Adams AC, Van Naarden J, Custer KL, Durante M, Oakley G, Schade AE, Holzer TR, Ebert PJ, Higgs RE, Kallewaard NL, Sabo J, Patel DR, Klekotka P, Shen L, Skovronsky DM (2021) Effect of Bamlanivimab as Monotherapy or in Combination With Etesevimab on Viral Load in Patients With Mild to Moderate COVID-19: A Randomized Clinical Trial. Jama 325 (7): 632-644 DOI 10.1001/jama.2021.0202

29 Grein J, Ohmagari N, Shin D, Diaz G, Asperges E, Castagna A, Feldt T, Green G, Green ML, Lescure FX, Nicastri E, Oda R, Yo K, Quiros-Roldan E, Studemeister A, Redinski J, Ahmed S, Bernett J, Chelliah D, Chen D, Chihara S, Cohen SH, Cunningham J, D'Arminio Monforte A, Ismail S, Kato H, Lapadula G, L'Her E, Maeno T, Majumder S, Massari M, Mora-Rillo M, Mutoh Y, Nguyen D, Verweij E, Zoufaly A, Osinusi AO, DeZure A, Zhao Y, Zhong L, Chokkalingam A, Elboudwarej E, Telep L, Timbs L, Henne I, Sellers S, Cao H, Tan SK, Winterbourne L, Desai P, Mera R, Gaggar A, Myers RP, Brainard DM, Childs R, Flanigan T (2020) Compassionate Use of Remdesivir for Patients with Severe Covid-19. N Engl J Med 382 (24): 2327-2336 DOI 10.1056/NEJMoa2007016

30 Terpos E, Ntanasis-Stathopoulos I, Elalamy I, Kastritis E, Sergentanis TN, Politou M, Psaltopoulou T, Gerotziafas G, Dimopoulos MA (2020) Hematological findings and complications of COVID-19. Am J Hematol 95 (7): 834-847 DOI 10.1002/ajh.25829

31 Rossi JF, Lu ZY, Jourdan M, Klein B (2015) Interleukin-6 as a therapeutic target. Clin Cancer Res 21 (6): 1248-1257 DOI 10.1158/1078-0432.Ccr-14-2291

32 Cevik M, Tate M, Lloyd O, Maraolo AE, Schafers J, Ho A (2021) SARS-CoV-2, SARS-CoV, and MERS-CoV viral load dynamics, duration of viral shedding, and infectiousness: a systematic review and meta-analysis. Lancet Microbe 2 (1): e13-e22 DOI 10.1016/S2666-5247(20)30172-5

33 Zheng S, Fan J, Yu F, Feng B, Lou B, Zou Q, Xie G, Lin S, Wang R, Yang X, Chen W, Wang Q, Zhang D, Liu Y, Gong R, Ma Z, Lu S, Xiao Y, Gu Y, Zhang J, Yao H, Xu K, Lu X, Wei G, Zhou J, Fang Q, Cai H, Qiu Y, Sheng J, Chen Y, Liang T (2020) Viral load dynamics and disease severity in patients infected with SARS-CoV-2 in Zhejiang province, China, January-March 2020: retrospective cohort study. Bmj 369: m1443 DOI 10.1136/bmj.m1443

34 Yu X, Sun S, Shi Y, Wang H, Zhao R, Sheng J (2020) SARS-CoV-2 viral load in sputum correlates with risk of COVID-19 progression. Crit Care 24 (1): 170 DOI 10.1186/s13054-020-02893-8

35 Herold T, Jurinovic V, Arnreich C, Lipworth BJ, Hellmuth JC, von Bergwelt-Baildon M, Klein M, Weinberger T (2020) Elevated levels of IL-6 and CRP predict the need for mechanical ventilation in COVID-19. J Allergy Clin Immunol 146 (1): 128-136.e124 DOI 10.1016/j.jaci.2020.05.008

36 Boger B, Fachi MM, Vilhena RO, Cobre AF, Tonin FS, Pontarolo R (2021) Systematic review with meta-analysis of the accuracy of diagnostic tests for COVID-19. Am J Infect Control 49 (1): 21-29 DOI 10.1016/j.ajic.2020.07.011

37 Basso D, Aita A, Navaglia F, Franchin E, Fioretto P, Moz S, Bozzato D, Zambon CF, Martin B, Dal Pra C, Crisanti A, Plebani M (2020) SARS-CoV-2 RNA identification in nasopharyngeal swabs: issues in pre-analytics. Clin Chem Lab Med 58 (9): 1579-1586 DOI 10.1515/cclm-2020-0749

38 (Assessed ) <https://www.who.int/diagnostics_laboratory/eul_0504-046-00_cobas_sars_cov2_qualitative_assay_ifu.pdf>. In: ed.

39 Berenger BM, Fonseca K, Schneider AR, Hu J, Zelyas N (2020) Sensitivity of Nasopharyngeal, Nasal and Throat Swab for the Detection of SARS-CoV-2. medRxiv: 2020.2005.2005.20084889 DOI 10.1101/2020.05.05.20084889

40 Accubiotech (Assessed 5th febuary 2022) <https://www.accubiotech.com/product-high-sensitivity-crp-semi-quantitative-rapid-test-cassette.html>. In: ed.

41 Eaglebio (Assessed 24th April 2022) <https://eaglebio.com/wp-content/uploads/2015/03/BI-IL6-Human-IL6-High-Sensitive-ELISA-Assay-Kit-Data-Validation.pdf>. In: ed.

42 FDA (Accessed 13th April 2022) <https://www.accessdata.fda.gov/cdrh_docs/reviews/K171498.pdf>. In: ed.

43 Cheng MP, Papenburg J, Desjardins M, Kanjilal S, Quach C, Libman M, Dittrich S, Yansouni CP (2020) Diagnostic Testing for Severe Acute Respiratory Syndrome-Related Coronavirus 2: A Narrative Review. Ann Intern Med 172 (11): 726-734 DOI 10.7326/m20-1301

44 FDA (assessed 25th June 2020) <https://www.accessdata.fda.gov/cdrh_docs/reviews/K171498.pdf>. In: ed.
